# Chronic Alcohol Exposure Among People Living with HIV Is Associated with Innate Immune Activation and Alterations in Monocyte Phenotype and Plasma Cytokine Profile

**DOI:** 10.3389/fimmu.2022.867937

**Published:** 2022-03-18

**Authors:** Michelle L. Underwood, Byung Park, Luke S. Uebelhoer, Geoffrey Gu, Lynn E. Kunkel, Philip T. Korthuis, Ryan R. Cook, Rafick Pierre Sekaly, Susan Pereira Ribeiro, Christina L. Lancioni

**Affiliations:** ^1^ Department of Pediatrics, Oregon Health & Science University, Portland, OR, United States; ^2^ Knight-Cancer Institute, Department of Biostatistics, Oregon Health & Science University, Portland, OR, United States; ^3^ Undergraduate Studies, University of Southern California, Los Angeles, CA, United States; ^4^ Department of Medicine, Oregon Health & Science University, Portland, OR, United States; ^5^ Department of Public Health, Oregon Health & Science University, Portland, OR, United States; ^6^ Department of Pathology & Translational Medicine, Emory University School of Medicine, Atlanta, GA, United States

**Keywords:** alcohol-use-disorder, innate immunity, inflammation, hepatitis C, human immuno deficiency virus (HIV)

## Abstract

Despite advances in antiretroviral therapy, chronic immune activation continues to be observed among individuals with well-controlled HIV viral loads, and is associated with non-AIDS defining morbidities among people living with HIV. Alcohol use disorder impacts a significant proportion of individuals living with HIV, and alcohol exposure is known to damage the intestinal epithelium which may increase translocation of pathogens and their molecular products, driving systemic immune activation and dysregulation. The aim of this study was to determine if adults living with HIV with well-controlled viral loads, who also suffer from alcohol use disorder with and without hepatitis C virus co-infection (n=23), exhibit evidence of advanced systemic immune activation, intestinal damage, and microbial translocation, as compared to adults living with HIV who are not exposed to chronic alcohol or other substances of abuse (n=29). The impact of a 1-month intervention to treat alcohol-use disorder was also examined. Alcohol-use disorder was associated with evidence of advanced innate immune activation, alterations in monocyte phenotype including increased expression of Toll-like receptor 4, increased burden of stimulatory ligands for Toll-like receptor 4, and alterations in plasma cytokine signature, most notably elevations in soluble CD40 ligand and transforming growth factor beta. Alcohol-associated immune activation was more pronounced among individuals with hepatitis C virus co-infection. Although the 1-month intervention to treat alcohol use disorder did not result in significant reductions in the interrogated indicators of immune activation, our findings suggest that chronic alcohol exposure is a major modifiable risk factor for chronic immune activation and dysregulation among people-living with HIV.

## Introduction

Chronic alcohol use resulting in alcohol-use disorder (AUD) is estimated to impact 14.5 million American adults and adolescents (1) who are at risk for adverse alcohol-related health effects. People living with HIV (PLWH) are particularly vulnerable to negative impacts of alcohol use, as heavy drinking is associated with reductions in HIV-treatment compliance and poor virologic control (2–4). A survey of over 7000 adult PLWH in the United States identified at-risk alcohol use among 16% of the population (5), emphasizing the importance of identifying AUD among PLWH and ensuring access to effective treatment to reduce alcohol-associated morbidities (6, 7).

Acute and chronic alcohol exposure compromises host defenses through mechanisms that place PLWH at increased risk for both acute infection and chronic inflammation (8). Both alcohol and HIV have long-term effects on the intestinal mucosa that impact systemic immunity and inflammation. Alcohol and acute HIV-infection independently damage the intestinal epithelium, increasing the translocation of bacterial products such as lipopolysaccharide (LPS) and other pathogen-associated molecular products (PAMPs) from the intestinal lumen into systemic circulation (9). PAMP translocation is a major driver of chronic immune activation, and is associated with non-AIDS co-morbidities and death among PLWH (10). Chronic systemic inflammation is further escalated among HIV/hepatitis C virus (HCV) co-infected individuals suffering from AUD (11–13).

Innate immune cells, such as monocytes, sense LPS and other microbial products using pattern-recognition receptors (PRRs), including Toll-like receptors (TLRs). Chronic alcohol exposure sensitizes the innate immune system to LPS and has been shown to alter phenotypic and functional characteristics of human monocytes (14, 15), resulting in a dysregulated pro-inflammatory immune response that may contribute to long term morbidities such as alcoholic liver disease (ALD), cardiovascular disease, neurocognitive decline, and malignancies (16). Alcohol also drives release of sterile danger signals from host cells in various tissues resulting in activation of immune cells *via* PRRs (17). Chronic alcohol exposure and HIV both independently alter the gut microbiome leading to an intestinal dysbiosis that may impact host immune homeostasis, increase CD8^+^ T-cell exhaustion, and drive chronic inflammation (18–20).

Chronic alcohol consumption may be a modifiable risk factor for immune dysregulation and chronic inflammation among PLWH with well controlled viral loads. We sought to investigate the impact of AUD and a 1-month pharmacologic intervention to treat AUD (21), on innate and adaptive immune phenotypes, plasma cytokine signature, systemic immune activation, intestinal damage, and microbial translocation among PLWH with and without HCV co-infection, as compared to PLWH without AUD or other substance-use disorder (SUD).

## Methods

### Participant Recruitment and Ethics Statement

PLWH with AUD (AUD+) were recruited through CTN-0055 CHOICES study from 2014 to 2015 (clinicaltrials.gov NCT01908062); an open-label, randomized, pilot trial of extended-release naltrexone (XR-NTX) versus treatment-as-usual (TAU) for treatment of opioid-use disorder (OUD), AUD, and mixed OUD/AUD in PLWH. Pre-intervention (week zero/W0) and 1-month post-intervention (week four/W4) blood samples from CTN-0055 participants with AUD were used for this analysis. TAU therapies for AUD included: oral naltrexone, gabapentin, acamprosate, or disulfiram. Of the 23 PLWH with AUD included in this analysis, all met DSM-V criteria for untreated, moderate-to-severe AUD. The mean pre-intervention addiction severity (ASI) alcohol score for individuals randomized to TAU was 0.33 (SD=0.32), versus ASI alcohol score of 0.41 (SD=0.32) among those randomized to XR-NTX (21). Median time since HIV-diagnosis among CTN-055 participants included in this analysis was 13 years (range 1-23 years; IQR 13 years). CTN-0055 was conducted by the National Institute on Drug Abuse (NIDA) Clinical Trials Network (CTN) and approved by Institutional Review Boards (IRB) at Oregon Health & Science University (OHSU) and pilot sites. PLWH without AUD or HCV co-infection (AUD-/HCV-; PLWH ref.) were recruited from OHSU HIV primary care clinic (2014–2016) through an independent, OHSU-approved IRB protocol. To be eligible for PLWH ref. cohort, individuals had confirmed HIV-infection, were 18–65 years of age, not pregnant, and denied current or recent (past 12 months) use of: opioids (including opioid-containing medications), cocaine, methamphetamines, daily cannabis, and daily alcohol. Relevant demographic and medical information including age, sex, race, tobacco use, CD4^+^ T-cell count, HIV viral load, hepatitis B virus and HCV serostatus, HCV PCR testing, use of ART, and diagnosis of OUD, SUD, or AUD in past 12 months, was obtained from participant medical records. Final HCV status was determined by HCV PCR test result. All study participants provided written, informed consent.

### PBMC and Plasma Processing and Storage

Up to 32 ml of peripheral blood was collected into CPT Vacutainer tubes (BD Biosciences, Franklin Lakes, New Jersey, USA). Samples were centrifuged within 2 h of collection, and PBMC and plasma mixed by inversion. Samples from CTN-0055 CHOICES participants were shipped at room temperature to a single laboratory, and plasma and PBMC processing completed within 24 h of collection. PBMC were cryopreserved at -150°C in 10% DMSO (Sigma-Aldrich, St Louis, Missouri, USA) in fetal bovine serum with 0.1% Gentamicin; undiluted plasma was stored at -80°C. Plasma and PBMC from PLWH ref. participants were collected, processed, and stored following an identical protocol, including a 24 h delay following centrifugation to replicate conditions for CTN-0055 CHOICES samples. The number of participants included in each assay are shown in figure legends and **Supplemental Tables 2, 3, 5, 7**.

### Flow Cytometry

The following reagents were utilized for T-cell phenotyping: cell viability (Live/Dead Fixable Blue, Invitrogen, Thermo-Fisher Scientific, Waltham, Massachusetts, USA), anti-CD14 (PE-Cy5, Invitrogen, Thermo-Fisher Scientific, clone M5E2), anti-CD19 (PE-Cy5, BD Biosciences, San Jose, California, USA, clone HIB19), anti-CD56 (PE-Cy5, BioLegend, San Diego, California, USA, clone HCD56), anti-CD3 (BUV737, BD Biosciences, clone UCHT2), anti-CD4 (APC-R700, BD Biosciences, clone SK3), anti-CD8 (BUV496, BD Biosciences, clone RPA-T8), anti-CD27 (BB700, BD Biosciences, clone M-T271), anti-CD45RA (APC, BD Biosciences, clone HI100), anti-CCR7 (BV650, BD Biosciences, clone 2L1A), anti-CD28 (BV786, BD Biosciences, clone 28.2), anti-CD38 (BV480, BD Biosciences, clone HIT2), anti-HLA-DR (BV421, BioLegend, clone L243), anti-CD57 (BB515, BD Biosciences, clone HNK1), anti-CCR5 (PE-CF594, BD Biosciences, clone 2D7), anti-PD-1 (PE-Cy7, BD Biosciences, clone EH12.1), anti-Lag-3 (PE, BD Biosciences, clone T47-530). For monocyte phenotyping only, to prevent non-specific binding, PBMC were incubated with Human Fc Block (BD Biosciences, clone Fc1.3216) following manufacturer’s instructions prior to antibody stain. The following reagents were utilized for monocyte phenotyping: cell viability (Live/Dead Fixable Blue, Invitrogen, Thermo-Fisher Scientific), anti-CD19 (PerCP, BioLegend, clone SJ25C1), anti-CD3 (PerCP, BioLegend, clone UCHT1), anti-CD56 (PerCP, BioLegend, clone HCD56), anti-CD14 (BUV737, BD Biosciences, clone M5E2), anti-CD16 (APC, BioLegend, clone 3G8), anti-CD15 (PE-CF594, BD Biosciences, clone W6D3), anti-CD11b (BV480, BD Biosciences, clone ICRF44), anti-HLA-DR (BUV395, BD Biosciences, clone G46-6), anti-TLR4 (BV421, BioLegend, clone HTA125), anti-PD-1 (BV650, BD Biosciences, clone EH12.1), anti-CD274 [(PD-L1) BB515, BD Biosciences, clone MIH1], anti-CCR2 (BV711, BD Biosciences, clone LS132.1D9), anti-CD163 (PE, IQ Products, Rozenburglaan, Groningen, Netherlands, clone MAC2-153). For all cellular phenotyping by flow cytometry, isotypes assessed for nonspecific binding; fluorescence minus one (FMO) controls were used to set gates for positive and negative staining. Cells were acquired using a BD Symphony, cytometer settings were checked using rainbow calibration particles (BD Biosciences) and a compensation matrix was prepared by using anti-mouse compensation particles that were stained with each corresponding antibody (BD Biosciences). Data was analyzed using FlowJo (v 10). Boolean gating calculated co-expression of markers. Cell-surface markers utilized to define monocyte and T-cell phenotypes are shown in **Supplemental Tables 3, 5**.

### Plasma ELISA

Plasma protein quantifications were performed using commercial kits according to the manufacturers’ instructions: soluble CD14 (R&D Systems, Minneapolis, Minnesota, USA), soluble CD163 (Invitrogen, Thermo-Fisher Scientific), Intestinal-fatty acid binding protein (I-FABP, R&D Systems), LPS-binding protein (LBP, R&D Systems), and soluble CD40L (Invitrogen, Thermo-Fisher Scientific). Experimental duplicates were performed for each sample.

### Plasma TLR-4 Ligands

HEK-Blue™ hTLR4 reporter cells with an inducible secreted alkaline phosphatase (SEAP) under the control of an IL-12 p40 minimal promoter fused to five NF-kB and AP-1 binding sites (InvivoGen, San Diego, California, USA, cat # hkb-htlr4) were used to determine concentration of stimulatory TLR-4 ligands in participant plasma according to manufacturer’s instructions. Briefly, early passage reporter cells were grown and maintained in DMEM, 4.5 g/l glucose, 10% (v/v) fetal bovine serum, 100 U/ml penicillin, 100 ug/ml streptomycin, 100 ug/ml Normocin™, and 2 mM L-glutamine, supplemented with 1X HEK-Blue™ Selection. Cells were grown to ~70-80% confluency, detached from flasks using a cell scraper and counted using a hemacytometer with trypan blue to discriminate dead cells. Cell suspensions were prepared at ~280,000 cells/ml in HEK-Blue™ Detection medium, and 50,000 cells added per well to a 96-well plate containing 20 μl of undiluted plasma samples or maintenance media (background) in duplicate. In addition, a standard curve was developed using known concentrations of lipopolysaccharide (LPS-EK ultrapure, *E. coli K12*; InvivoGen cat # tlrl-peklps) prepared in triplicate with sterile ultrapure Ambion™ DEPC-treated water (Invitrogen) using a top concentration of 20,000 pg/ml and diluted five-fold. Plates were incubated for 18 hours at 37°C + 5% CO2 and SEAP determined using a spectrophotometer at 630nm. This assay is subsequently referred to as HEK-Blue hTLR4 assay.

### Plasma Cytokine/Chemokine Assay

Plasma cytokines were quantified using the commercially available U-PLEX assay (Meso Scale MULTI-ARRAY technology; Meso Scale Discovery; Rockville, MD, USA). Using this platform, plasma IFN-γ, IL-8, IP-10, Fractalkine, GM-CSF, IL-12p70, IL-17A, IL-18, IL-1β, IL-2, TGF-β1, TGF-β2, TGF-β3, IFN-α2a, IFN-β, TNF-α, IL-10, IL-6, IL-9, IL-15, IL-21, IL-22, IL-23, IL-27, IL-33, IL-4, IL-7, ITAC, MCP-3, MIP1-α, MIP3-α, SDF1-α, and IL-29, were quantified. Duplicate samples using 25 μL of plasma from each donor were assessed following the manufacturer’s instructions. Electrochemiluminescence was detected using MESO QuickPlex SQ 120 (Meso Scale Discovery Rockville, MD, United States). The results were extrapolated from the standard curve from each specific analyte and plotted in pg/mL using the DISCOVERY WORKBENCH v4.0 software (Meso Scale Discovery, Rockville, MD, United States).

### Statistical Analysis

Participant demographic and clinical characteristics were presented as medians with interquartile range (IQR) for continuous variables and compared using t-tests; chi-squared or Fisher’s exact testing was applied to compare dichotomous outcomes (**Table 1**). Prior to application of normal distribution-based statistical models, distribution of data for all immunologic variables was examined for normality; log2 transformation was performed for outcomes of IFABP, LBP, plasma TLR-4 ligands as quantified by HEK-Blue hTLR4 assay, sCD14, and sCD163 to achieve normal distribution. Factorial Analysis of Variance (ANOVA) was used to compare cohorts on immunologic and flow cytometry outcomes detailing monocyte and T-cell phenotypes. Tukey-Cramer adjustment was employed to control family wise error rate. For plasma cytokines, non-parametric approaches were applied (Kruskal Wallis) due to the skewed nature of distribution and occurrence of undetectable (zero) values for some analytes. The Dwass, Steel, Critchlow-Fligner (DSCF) multiple comparison analysis, which is based on pairwise two-sample Wilcoxon comparisons (22–24), was used to control overall type I error rate at 0.05. To determine if HCV co-infection had a significant interaction with each immunologic outcome, we first compared each immunologic outcome between individuals with and without HCV co-infection using either Kruskal-Wallis or factorial ANOVA model. If a significant interaction (p< 0.05) with HCV was observed for an outcome, subsequent comparisons between AUD+ CTN-055 and PLWH ref. cohort was performed with AUD+/HCV- and AUD+/HCV+ considered as separate populations. If no significant interactions with HCV status were detected, comparisons between AUD+ CTN-055 and PLWH ref. cohort was performed without further consideration for HCV status. Comparisons among study cohorts that remained significant following correction for multiple comparisons are shown in **Tables 3**, **5** when no significant interactions with HCV status were identified, and **Tables 2**, **4** if an interaction with HCV was found. Spearman correlation analysis was performed to investigate relationships between selected immunologic variables (see **Supplemental Tables 4, 6**). All descriptive statistics, and unadjusted and adjusted cohort comparisons, can be found in **Supplemental Tables 1, 2, 3, 5, 7**.

**Table 1:** Participant demographic and clinical characteristics.

	CTN-055 CHOICES HIV+AUD+ n=23	Reference population HIV+AUD- n=29	p-value
Age	47.7 (IQR: 14)^1^	46 (IQR: 19.5)^1^	>0.05^2^
Sex (female)	8 (37.8%)	1 (3.5%)	0.0068^3^
Hepatitis C	8 (37.8%)	0 (0%)	0.0007^3^
On ART	23 (100%)	29 (100%)	>0.05
CD4 Count	680 (IQR: 291)^1^	582 (IQR: 480.5)^1,4^	>0.05^2^
Undetectable viral load^5^	22 (95.7%)	23 (79.3%)^4^	>0.05^3^
Tobacco use reported	16 (69.5%)	7 (24.1%)	0.001^6^

^1^Median with IQR reported.

^2^T-test.

^3^Fisher’s exact.

^4^Data not collected for 1 donor.

^5^Among CTN-055 participants, 1 individual had a HIV viral load reported as weak positive. Among reference participants, 1 individual had a HIV viral load of 67 copies/ml and 5 had weak positive HIV viral load reported.

^6^Chi-square.

**Table 2:** Significant differences in soluble mediators of inflammation between PLWH with and without AUD by HCV co-infection.

Soluble mediators	Comparison Cohorts	Lower 95% CI (adjusted)	Upper 95% CI (adjusted)	p-value (adjusted)
Log2 sCD163	W0: AUD+/HCV+ vs PLWH Ref.	0.3404	1.3601	**0.0001**
	W0: AUD+/HCV- vs PLWH Ref.	0.0151	0.8272	**0.0379**
	W4: AUD+/HCV+ vs PLWH Ref.	0.6300	1.6497	**<0.0001**
	W4: AUD+/HCV- vs PLWH Ref.	0.0911	0.9032	**0.0081**
	W0-W4: AUD+/HCV+	-0.9279	0.3488	0.7202
	W0-W4: AUD+/HCV-	-0.5422	0.3902	0.9914
Log2 LBP	W0: AUD+/HCV+ vs PLWH Ref.	0.0470	1.0401	**0.0243**
	W0: AUD+/HCV- vs PLWH Ref.	-0.0189	0.7720	0.0702
	W4: AUD+/HCV+ vs PLWH Ref.	-0.0327	0.9605	0.0794
	W4: AUD+/HCV- vs PLWH Ref.	0.0455	0.8364	**0.0206**
	W0-W4: AUD+/HCV+	-0.5421	0.7014	0.9966
	W0-W4: AUD+/HCV-	-0.5185	0.3896	0.9950
Log2 HEK Blue	W0: AUD+/HCV+ vs PLWH Ref.	0.6052	1.9415	**<0.0001**
	W0: AUD+/HCV- vs PLWH Ref.	0.1718	1.2629	**0.0036**
	W4: AUD+/HCV+ vs PLWH Ref.	0.1035	1.4398	**0.0149**
	W4: AUD+/HCV- vs PLWH Ref.	-0.0434	1.0477	0.0868
	W0-W4: AUD+/HCV+	-0.2969	1.3003	0.4144
	W0-W4: AUD+/HCV-	-0.3681	0.7984	0.8453

Bold values identify statistically significant comparisons.

## Results

### Participant Characteristics

Twenty-three PLWH with AUD enrolled in CTN-055 CHOICES (21), and a reference population of 29 PLWH without AUD or other self-reported SUD (AUD-/HCV-; PLWH ref.), were included. HCV co-infection was common among CTN-055 participants, whom were also more likely to be female and report tobacco use, as compared to PLWH ref. cohort (**Table 1**). Data regarding racial composition also identified differences between cohorts. Within CTN-055, 39.1% of participants identified as Black or African American, 30.4% as white, and 30.4% as American Indian, Alaska Native, Native Hawaiian or Pacific Islander. Within the PLWH ref. cohort, 86.2% of individuals identified as white; 4 individuals within this group declined to provide self-identified racial information. HIV viral load, age, and CD4 count were similar between populations, and all individuals were prescribed ART. CTN-055 participants were randomized to receive AUD treatment with either XR-NTX versus TAU (n=10 and n=13, respectively); given limitations in sample size, treatment arms were combined for further analysis. Using a 30-day Timeline Follow-back method capturing median number of self-reported days of drinking, we identified a significant decrease in drinking days from study entry (W0; 17 days) to week 4 (W4; 4 days; p<0.001, Wilcoxon signed-rank) among CTN-055 participants.

### Elevated Cell-Surface and Soluble CD163 Among PLWH with Alcohol-Use Disorder

**Figure 1:**
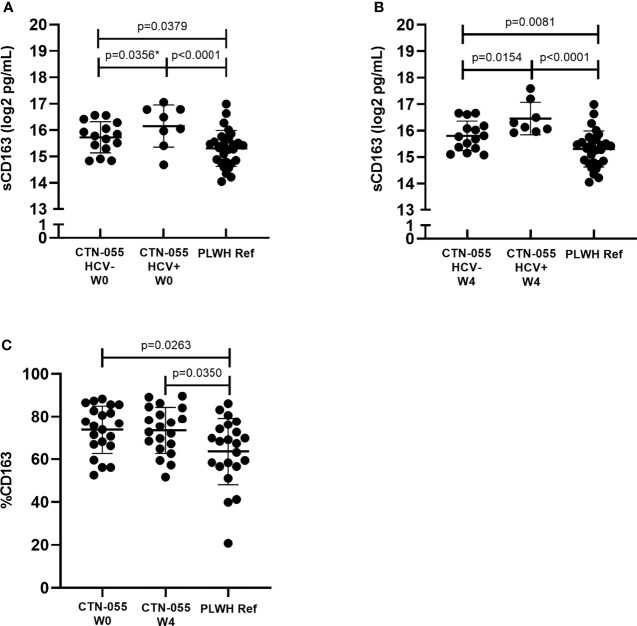
Alcohol-use disorder is associated with alterations in cell-surface and soluble CD163 Among PLWH. Soluble CD163 was quantified by ELISA in plasma collected from CTN-055 participants at W0 (**A**; n = 23) and W4 (**B**; n = 23) timepoints, and PLWH ref. population (n = 29) at study enrollment. Shown are log2 transformed data, with means and SD. CD163 expression was analyzed on CD14^+^ monocytes using flow cytometry among CTN-055 participants at W0 (n = 21) and W4 (n = 20), and PLWH ref. population (n = 22) at study enrollment; shown are means with SD of total CD14^+^CD163^+^ monocytes **(C)**. *indicates unadjusted p-value.

CD163 is a cell-surface receptor shed from the surface of activated monocytes and macrophages, that is reflective of chronic innate immune activation and disease activity among PLWH (25). Soluble CD163 (sCD163) values were significantly elevated between AUD+/HCV- and AUD+/HCV+ CTN-055 as compared to PLWH ref. participants, at both W0 (Adj. p=0.0379 and Adj. p<0.001, respectively) and W4 timepoints (Adj. p=0.0081 and Adj. p<0.001, respectively; **Figure 1** and **Table 2**). Soluble CD163 values were higher among AUD+/HCV+ as compared to AUD+/HCV- CTN-055 participants at both timepoints (W0 Unadj. p=0.0356; W4 Adj. p=0.0154; **Figure 1** and **Table 2**). Monocyte surface CD163 expression was quantified by flow cytometry, and found to be significantly elevated on the total CD14^+^ monocyte population among CTN-055 participants regardless of HCV co-infection, as compared to PLWH ref. at both W0 (Adj. p=0.0263) and W4 (Adj. p=0.0350; **Figure 1** and **Table 3**). Additional details available in **Supplemental Tables 1–3**.

**Table 3:** Significant differences in cellular phenotypes between PLWH with and without AUD.

Cellular Phenotypes	Comparison Cohorts	Lower 95% CI (adjusted)	Upper 95% CI (adjusted)	p-value (adjusted)
Total CD14+ CD163+	W0: AUD+ vs PLWH Ref.	1.0028	19.4129	**0.0263**
	W4: AUD+ vs PLWH Ref.	0.5728	19.2220	**0.0350**
	W0-W4: AUD+ only	-9.2184	9.8393	0.9966
Classical Monocytes (CD14++CD16-)	W0: AUD+ vs PLWH Ref.	-15.4882	1.4521	0.1232
	W4: AUD+ vs PLWH Ref.	-23.1160	-5.9558	**0.0004**
	W0-W4: AUD+ only	-1.2502	16.2860	0.1069
Intermediate Monocytes (CD14++CD16+)	W0: AUD+ vs PLWH Ref.	-1.5102	15.5388	0.1267
	W4: AUD+ vs PLWH Ref.	5.7075	22.9779	**0.0005**
	W0-W4: AUD+ only	-16.1528	1.4959	0.1221
Non-classical Monocytes (CD14^dim^CD16+) CCR2 mid	W0: AUD+ vs PLWH Ref.	2.6843	23.4259	**0.0101**
	W4: AUD+ vs PLWH Ref.	5.0113	26.0222	**0.0021**
	W0-W4: AUD+ only	-13.1973	8.2740	0.8465
Non-classical Monocytes (CD14^dim^CD16+) CD163+	W0: AUD+ vs PLWH Ref.	-1.8403	15.8466	0.1467
	W4: AUD+ vs PLWH Ref.	1.8791	19.7957	**0.0139**
	W0-W4: AUD+ only	-12.9888	5.3202	0.5759
Non-classical Monocytes (CD14^dim^CD16+) PD-1L+	W0: AUD+ vs PLWH Ref.	1.1227	12.4916	**0.0151**
	W4: AUD+ vs PLWH Ref.	-1.7357	9.7807	0.2218
	W0-W4: AUD+ only	-3.0997	8.6690	0.4954
CD3+ CD4+ Naive (CD45RA+ CCR7+) CD27+ Geo Mean	W0: AUD+ vs PLWH Ref.	141.490	2280.720	**0.0227**
	W4: AUD+ vs PLWH Ref.	73.857	2213.080	**0.0335**
	W0-W4: AUD+ only	-1013.800	1149.070	0.9877
CD3+ CD4+ Central memory (CD45RA- CCR7+) CD27+ Geo Mean	W0: AUD+ vs PLWH Ref.	243.180	2275.220	**0.0114**
	W4: AUD+ vs PLWH Ref.	131.180	2163.220	**0.0232**
	W0-W4: AUD+ only	-915.250	1139.250	0.9630
CD3+ CD8+ Naive (CD45RA+ CCR7+) CD27+ Geo Mean	W0: AUD+ vs PLWH Ref.	683.570	2831.890	**0.0006**
	W4: AUD+ vs PLWH Ref.	737.160	2885.480	**0.0004**
	W0-W4: AUD+ only	-1139.620	1032.440	0.9923
CD3+ CD8+ Central memory (CD45RA- CCR7+) CD27+ Geo Mean	W0: AUD+ vs PLWH Ref.	31.418	1807.760	**0.0408**
	W4: AUD+ vs PLWH Ref.	-139.760	1636.580	0.1152
	W0-W4: AUD+ only	-726.810	1069.170	0.8913

Bold values identify statistically significant comparisons.

### AUD is Associated with Increased Monocyte Expression of TLR-4

**Figure 2:**
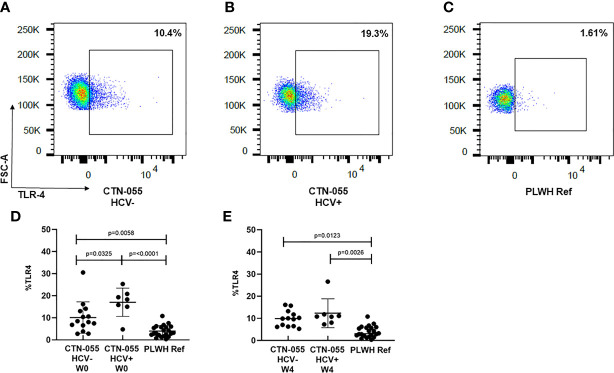
Alcohol-use disorder Among PLWH is associated with increased monocyte TLR-4 cell-surface expression. TLR-4 expression was analyzed on the total CD14^+^ monocyte population using flow cytometry. Representative data are shown in CTN-055 HCV- **(A)**, HCV+ **(B)** and PLWH reference participants **(C)**. Means with SD of total CD14+TLR-4+ monocytes from all participants are shown at W0 (**D**; n = 21) and W4 (**E**; n = 20), and PLWH ref. population (n = 22) at study enrollment.

TLR-4 is a PRR utilized by monocytes and other innate immune cells to detect LPS present on gram-negative bacteria, as well as endogenous danger signals produced by injured tissues (26). TLR-4 activation triggers two pro-inflammatory signaling cascades using distinct adaptor proteins; a plasma membrane cascade involving TIRAP and MyD88, and an endosomal cascade involving TRAM and TRIF. TLR-4-initiated inflammation is regulated through multiple mechanisms; however, the rate of TLR-4 endocytosis is reported to be a major regulator of LPS-induced inflammation, and exaggerated activation of TLR-4 is associated with multiple human diseases (27). Here we noted marked increase in monocyte TLR-4 surface expression among both AUD+/HCV- and AUD+/HCV+ CTN-055 participants as compared to PLWH ref. participants at W0 (Adj. p=0.0058 and Adj. p ≤0.0001, respectively) and W4 (Adj. p=0.0123 and Adj. p=0.0026, respectively; **Figure 2** and **Table 4**). Surface expression of TLR-4 was also significantly higher among AUD+/HCV+ as compared to AUD+/HCV- individuals at W0 (Adj. p=0.0325; **Figure 2**; **Table 4**; **Supplemental Figure 1**). Notably, expression of TLR-4 was correlated with sCD163 plasma levels (r_s_=0.37; p=0.0028). Additional details available in **Supplemental Tables 1, 3, 4**.

**Table 4:** Significant differences in cellular phenotypes between PLWH with and without AUD by HCV co-infection.

Cellular Phenotypes	Comparison Cohorts	Lower 95% CI (adjusted)	Upper 95% CI (adjusted)	p-value (adjusted)
Total CD14+ TLR4+	W0: AUD+/HCV+ vs PLWH Ref.	6.9563	19.1960	**0.0000**
	W0: AUD+/HCV- vs PLWH Ref.	1.3333	10.9762	**0.0058**
	W4: AUD+/HCV+ vs PLWH Ref.	2.2563	14.4960	**0.0026**
	W4: AUD+/HCV- vs PLWH Ref.	0.9193	10.7862	**0.0123**
	W0-W4: AUD+/HCV+	-2.8382	12.2382	0.4091
	W0-W4: AUD+/HCV-	-5.1299	5.7338	0.9999
Total CD14+ CCR2 bright	W0: AUD+/HCV+ vs PLWH Ref.	-7.9606	4.8318	0.9582
	W0: AUD+/HCV- vs PLWH Ref.	-5.5064	4.5719	0.9989
	W4: AUD+/HCV+ vs PLWH Ref.	-6.7792	6.0132	0.9998
	W4: AUD+/HCV- vs PLWH Ref.	-10.5174	-0.2049	**0.0377**
	W0-W4: AUD+/HCV+	-9.0600	6.6972	0.9932
	W0-W4: AUD+/HCV-	-0.7833	10.5710	0.1227
Classical Monocytes (CD14++CD16-) TLR4+	W0: AUD+/HCV+ vs PLWH Ref.	7.4367	23.2936	**<0.0001**
	W0: AUD+/HCV- vs PLWH Ref.	0.2881	12.7808	**0.0360**
	W4: AUD+/HCV+ vs PLWH Ref.	2.2038	18.0607	**0.0058**
	W4: AUD+/HCV- vs PLWH Ref.	0.1154	12.8984	**0.0440**
	W0-W4: AUD+/HCV+	-4.5331	14.9988	0.5612
	W0-W4: AUD+/HCV-	-7.0096	7.0647	1.0000
Non-classical Monocytes (CD14^dim^CD16+)	W0: AUD+/HCV+ vs PLWH Ref.	-0.6499	3.0902	0.3628
	W0: AUD+/HCV- vs PLWH Ref.	-0.1925	2.7542	0.1173
	W4: AUD+/HCV+ vs PLWH Ref.	-0.6842	3.0559	0.3918
	W4: AUD+/HCV- vs PLWH Ref.	1.3582	4.3733	**<0.0001**
	W0-W4: AUD+/HCV+	-2.2692	2.3378	1.0000
	W0-W4: AUD+/HCV-	-3.2447	0.0749	0.0680
Non-classical Monocytes (CD14^dim^CD16+) HLA-DR Geo Mean	W0: AUD+/HCV+ vs PLWH Ref.	-581.3600	8416.4300	0.1163
	W0: AUD+/HCV- vs PLWH Ref.	-1134.3000	5954.5100	0.3214
	W4: AUD+/HCV+ vs PLWH Ref.	1395.5000	10393.0000	**0.0044**
	W4: AUD+/HCV- vs PLWH Ref.	-708.2400	6545.2600	0.1710
	W0-W4: AUD+/HCV+	-7518.4300	3564.7200	0.8523
	W0-W4: AUD+/HCV-	-4501.5300	3484.7200	0.9964

Bold values identify statistically significant comparisons.

### Evidence Supporting Increased Systemic Burden of LPS Among Individuals with Alcohol-Use Disorder

**Figure 3:**
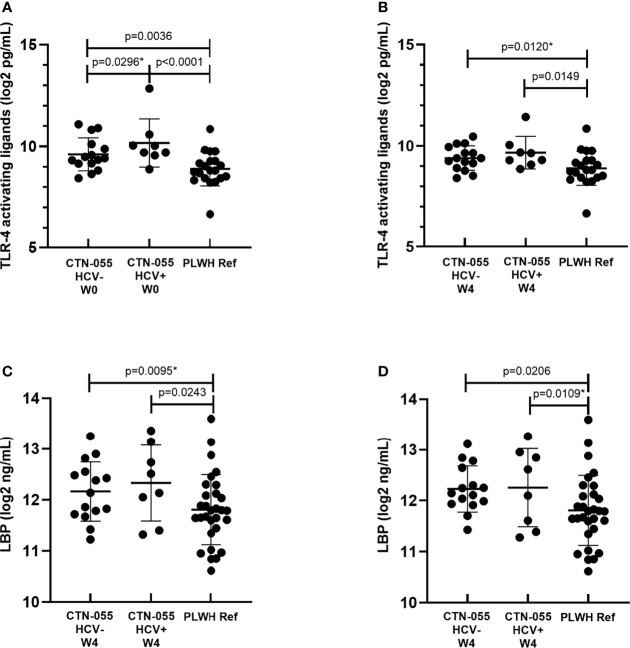
Alcohol-use disorder Among PLWH is associated with increased levels of plasma TLR-4-activating ligands and LPS-binding protein. Plasma concentrations of stimulatory TLR-4 ligands were estimated using commercially available human TLR-4 transfected-HEK reporter cells (HEK-Blue hTLR4) and a standard curve generated using known quantities of ultrapure LPS, at W0 (**A**; n = 23) and W4 (**B**; n = 23) timepoints, and PLWH ref. population (n = 20) at study enrollment. LPS-binding protein (LBP) was quantified by ELISA in plasma collected from CTN-055 participants at W0 (**C**; n = 23), W4 (**D**; n = 23) and PLWH ref. population (n = 29). Shown are means with SD. *indicates unadjusted p-value.

The capacity of LPS to elicit TLR-4-mediated immune activation is dependent on several factors, including the biochemical composition of its lipid A moiety that varies among gram negative bacteria (28). To estimate the burden of stimulatory TLR-4 ligands in participant plasma, two methods were applied: the HEK-Blue hTLR4 assay, and quantification of LBP, which serves as an indirect reflection of heightened plasma LPS. Here we noted increased plasma TLR-4 stimulatory activity using the HEK-Blue hTLR4 assay among AUD+/HCV+ and AUD+/HCV-versus PLWH ref. participants at W0 (Adj. p<0.0001 and Adj. p=0.0036, respectively; **Figure 3** and **Table 2**). At the W0 timepoint, there was also a trend towards higher HEK-Blue hTLR4 results between AUD+/HCV+ and AUD+/HCV- participants (Unadj. p=0.029). At W4, the burden of TLR-4 ligands remained significantly elevated in AUD+/HCV+ as compared to PLWH ref. participants (Adj. p=0.0149), with a trend towards a higher burden observed between AUD+/HCV- and PLWH ref. participants (Unadj. p=0.012). LBP values were higher among AUD+/HCV- and AUD+/HCV+ CTN-055 versus PLWH ref. participants at both W0 (Unadj. p=0.0095 and Adj. p=0.0243, respectively) and W4 (Adj. p=0.0206 and Unadj. p=0.0109 respectively; **Figure 3** and **Table 2**). Among all participants, the burden of plasma TLR-4-activating ligands was correlated with plasma LBP (r_s_=0.43; p=0.0003) and sCD163 (r_s_=0.37; p=0.0028). A trend towards increased plasma I-FABP values, reflective of damage to the intestinal epithelium, was noted among AUD+/HCV+ CTN-055 versus PLWH ref. participants at W0 only (Unadj. p=0.019). Additional details available in **Supplemental Tables 1, 2, 4**.

### Expansion of CD16+ Monocytes Among PLWH with Alcohol-Use Disorder

**Figure 4:**
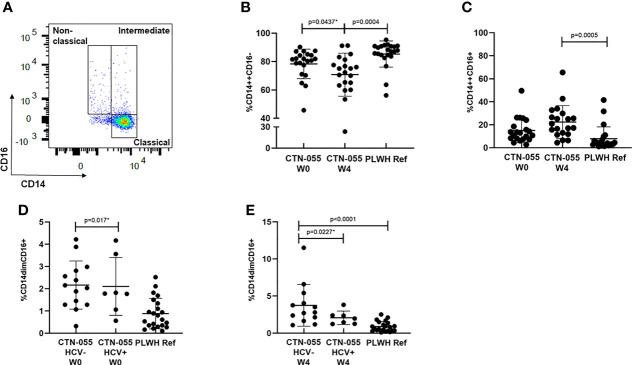
Alcohol-use disorder Among PLWH is associated with altered monocyte phenotype. Boolean gating was applied to total live, CD3-/CD56-/CD19-/CD14+ cells to calculate frequencies of classical, intermediate, and non-classical monocytes **(A)** among CTN-055 participants at W0 (n = 21) and W4 (n = 20), and PLWH ref. population (n = 22). Means with SD are shown for each monocyte phenotype from all participants **(B–E)**. See **Supplemental Figure 1** for complete gating strategy. *indicates unadjusted p-value.

CD16 is a cell-surface Fcγ receptor that facilitates antibody-dependent cellular cytotoxicity among non-classical and intermediate monocytes. Monocytes that express CD16 are recognized to have heightened pro-inflammatory responses, to have increased susceptibility to HIV-infection, and to promote viral replication *in vitro* (29–31). To determine if AUD was associated with altered monocyte phenotype among PLWH, monocytes were identified in PBMC by flow cytometry based on light scatter properties and expression of CD14^+^ and CD16^+^, and classified as exhibiting classical (CD14^++^CD16^-^), intermediate (CD14^++^CD16^+^), and nonclassical (CD14^dim^CD16^+^) phenotypes as previously reported (**Figure 4** and **Supplemental Figure 1**) (31). Here we found a trend towards a declining frequency of classical monocytes among CTN-055 participants from W0 to W4 (Unadj. p=0.0437), with the frequency of classical monocytes significantly lower among CTN-055 at W4 as compared to PLWH ref. participants (Adj. p=0.0004; **Figure 4** and **Table 3**). There was a significant increase in CD16^+^ intermediate monocytes at W4 among CTN-055 as compared to PLWH ref. participants (Adj. p=0.0005; **Figure 4** and **Table 3**). These findings did not vary by HCV co-infection. The frequency of CD16^+^ non-classical monocytes did vary by HCV co-infection, with expansion of non-classical monocytes among AUD+/HCV- CTN-055 compared to PLWH ref. participants at both time points (W0 Unadj. p=0.017; W4 Adj. p < 0.0001), and expansion of non-classical monocytes among HCV- CTN-055 as compared to HCV+ participants at W4 (Unadj. P=0.0227; **Figure 4** and **Table 4**). Variable expression of several other surface receptors, specifically CCR2, PD-1L, and HLA-DR, was noted across different monocyte subtypes based on both AUD and HCV exposure (**Tables 3**, **4**). Additional details available in **Supplemental Tables 1, 3** and **Supplemental Figure 1**.

### Increased CD4^+^ and CD8^+^ T Cell Expression of CD27 Among PLWH with Alcohol-Use Disorder

CD4^+^ and CD8^+^ T-cell phenotypes indicating maturation stage, activation and exhaustion status, and immunosenescence, were examined in detail by flow cytometry. Overall, T-cell phenotypes (including overall percentages of total CD3^+^CD4^+^ and CD3^+^CD8^+^ T cells, see **Supplemental Tables 1, 5**) were similar between CTN-055 and PLWH ref. participants, with the exception of CD27 expression. As shown in **Table 2**, we identified increased expression of CD27 (as quantified by geometric mean fluorescence) on CD4^+^ and CD8^+^ naïve (CD45RA^+^CCR7^+^) T-cells from CTN-055 as compared to PLWH ref. participants at both W0 (CD4^+^, Adj. p=0.0227; CD8^+^, Adj. p=0.0006) and W4 (CD4^+^, Adj. p=0.0335; CD8^+^, Adj. p=0.0004). Increased expression of CD27 was also noted on central memory CD4^+^ and CD8^+^ (CD45RA^-^CCR7^+^) T-cells from CTN-055 as compared to PLWH ref. participants at both W0 (CD4^+^, Adj. p=0.0114; CD8^+^, Adj. p=0.0408) and W4 (CD4^+^, Adj. p=0.0232; CD8^+^, Unadj. p=0.0474).

### Alcohol-Use Disorder is Associated with Increased Plasma sCD40L and TGF-β

**Figure 5:**
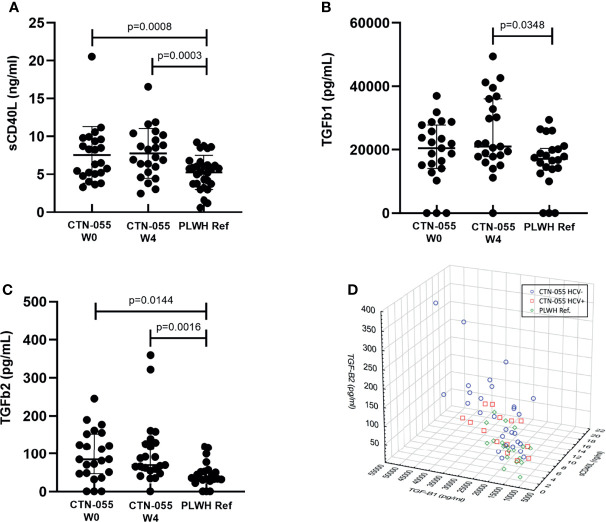
Alcohol-use disorder Among PLWH is associated with an increased level of soluble CD40L, TGF-β1 and TGF-β2. Plasma levels of soluble CD40L **(A)**, and TGF-β1 **(B)** and TGF-β2 **(C)** were quantified by standard ELISA and cytokine U-PLEX assay, respectively, among CTN-055 participants at W0 (n = 23) and W4 (n = 23), and PLWH ref. population (n = 22) at study enrollment. Shown are means with SD **(A)** or medians with IQR **(B, C)**. When soluble CD40L, TGF-β1, and TGF-β2 were examined simultaneously, distinct clustering of study participants based on exposure to AUD and HCV was noted **(D)**.

CD40L, or CD154, is a membrane glycoprotein expressed by a variety of cell types, including activated T- and B-cells, monocytes, macrophages, endothelial cells, and platelets (32). Soluble CD40L (sCD40L) is shed from the surface of activated platelets and T-cells. Activated platelets are the primary source of sCD40L in human plasma (33), and sCD40L levels are associated with sequelae of chronic HIV-infection (34–36). TGF-beta (TGF-β) is a large family of pleiotropic cytokines involved in development, wound healing, cellular differentiation and proliferation, as well as immune regulation; three isoforms of TGF-β have been well-studied in humans (37). Notably, TGF-β has been linked to the pathogenesis of a broad range of liver disorders, including ALD (38). Soluble CD40L, TGF-β1 and TGF-β2 were quantified by ELISA and plasma cytokine U-PLEX assay, respectively. Soluble CD40L and TGF-β2 levels were significantly higher among CTN-055 as compared to PLWH ref. participants at both W0 (Adj. p=0.0008 and Adj. p=0.0144, respectively) and W4 (Adj. p=0.0003 and Adj. p=0.0016, respectively; **Figure 5** and **Table 5**). Significant elevations in TGF-β1 were noted at W4 among CTN-055 participants (Adj. p=0.0348; **Figure 5** and **Table 5**); these findings did not vary by HCV co-infection. Notably, plasma levels of TGF-β1 were highly correlated with the number of self-reported drinking days at both week 0 (r_s_=0.555; p-value 0.006) and week 4 (r_s_= 0.698; p-value 0.0002) among CTN-055 participants. Additional details available in **Supplemental Tables 1, 2, 7**.

**Table 5:** Significant differences in plasma cytokines between PLWH with and without AUD.

Soluble Mediators	Comparison Cohorts	Lower 95% CI (adjusted)	Upper 95% CI (adjusted)	p-value (adjusted)
sCD40L	W0: AUD+ vs PLWH Ref.	0.8403	3.7789	**0.0008**
	W4: AUD+ vs PLWH Ref.	0.9974	3.9542	**0.0003**
	W0-W4: AUD+ only	-1.7266	1.3941	0.9655
Fraktaline	W0: AUD+ vs PLWH Ref.	N/A*	N/A*	0.1324
	W4: AUD+ vs PLWH Ref.	N/A*	N/A*	**0.0115**
	W0-W4: AUD+ only	N/A*	N/A*	0.9711
IL-12p70	W0: AUD+ vs PLWH Ref.	N/A*	N/A*	0.8476
	W4: AUD+ vs PLWH Ref.	N/A*	N/A*	**0.0194**
	W0-W4: AUD+ only	N/A*	N/A*	0.1686
IL-1β	W0: AUD+ vs PLWH Ref.	N/A*	N/A*	**0.0244**
	W4: AUD+ vs PLWH Ref.	N/A*	N/A*	0.2837
	W0-W4: AUD+ only	N/A*	N/A*	0.3490
MCP-3	W0: AUD+ vs PLWH Ref.	N/A*	N/A*	**0.0171**
	W4: AUD+ vs PLWH Ref.	N/A*	N/A*	0.1044
	W0-W4: AUD+ only	N/A*	N/A*	0.7409
MIP-3α	W0: AUD+ vs PLWH Ref.	N/A*	N/A*	0.5840
	W4: AUD+ vs PLWH Ref.	N/A*	N/A*	**0.0291**
	W0-W4: AUD+ only	N/A*	N/A*	0.3377
TFG-β1	W0: AUD+ vs PLWH Ref.	N/A*	N/A*	0.4474
	W4: AUD+ vs PLWH Ref.	N/A*	N/A*	**0.0348**
	W0-W4: AUD+ only	N/A*	N/A*	0.4096
TGF-β2	W0: AUD+ vs PLWH Ref.	N/A*	N/A*	**0.0144**
	W4: AUD+ vs PLWH Ref.	N/A*	N/A*	**0.0016**
	W0-W4: AUD+ only	N/A*	N/A*	1.0000

*Not applicable (N/A): confidence intervals not reported for nonparametric analyses.

Bold values identify statistically significant comparisons.

Spearman correlation analysis between plasma sCD40L, TGF-β1, and TGF-β2 identified significant direct correlations between sCD40L and TGF-β1 among all cohorts, and a significant correlation between sCD40L and TGF-β2 among AUD+/HCV- participants specifically. When plasma values of sCD40L, TGF-β1 and TGF-β2 were examined simultaneously, clustering based on AUD and HCV exposure was observed (**Figure 5**). Further correlation analysis between TGF-β1, TGF-β2 and all quantified plasma cytokines revealed additional significant, direct relationships. Additional details available in **Supplemental Tables 6, 7**.

Regarding additional plasma cytokine values (see **Table 5**), IL-1β was significantly elevated among CTN-055 as compared to PLWH ref. participants at W0 (Adj. p=0.0244), and fractalkine and IL-12p70 were noted to be significantly elevated at W4 (Adj. p=0.0115 and Adj. p=0.0194, respectively). Plasma IL-29 was significantly higher among AUD+/HCV+ compared to AUD+/HCV- participants at both timepoints (W0 Adj. p=0.0117 and W4 Adj. p=0.0426). Additional details available in **Supplemental Tables 1, 7**.

## Discussion

Chronic alcohol exposure is recognized to impair host defenses. Both animal model and human studies have demonstrated that alcohol-induced damage to the intestinal epithelium leads to increases in gut permeability, with systemic translocation of microbes and their by-products that drive intestinal dysbiosis and systemic innate immune activation (14, 17, 19, 39–45). Here, we performed a comprehensive evaluation of the impact of chronic alcohol exposure on innate and adaptive immune activation and exhaustion among PLWH specifically, as these individuals are especially vulnerable to chronic immune dysregulation mediated through intestinal translocation and dysbiosis even when HIV viral loads are well-controlled (9, 18, 46). Utilizing samples obtained from virally-suppressed PLWH with AUD (21), and a reference population of virally-suppressed PLWH without AUD or other SUD, we found a strong association between chronic alcohol exposure and systemic indicators of immune activation. Our significant findings included alcohol-associated elevations in soluble and cell-surface CD163, monocyte TLR-4 expression, sCD40L, and plasma TLR-4 stimulatory ligands and LBP. Additional alterations in monocyte phenotype, including expansion of CD16^+^ subsets, were noted among PLWH with AUD, as was increased expression of CD27 among naïve and memory CD4^+^ and CD8^+^ T-cells. Chronic alcohol exposure among PLWH was also associated with alterations in plasma cytokines, most notably increases in TGF-β1 and TGF-β2. These data suggest that chronic alcohol exposure is a major modifiable risk factor for chronic immune activation and dysregulation among PLWH.

Given the damaging impacts of HIV-infection and chronic alcohol exposure on intestinal permeability, our investigations focused on identifying indicators of chronic immune activation and dysregulation among CTN-055 participants with AUD. We hypothesized that monocytes among PLWH with AUD would be impacted by LPS-induced immune activation, a finding supported by our investigations of increased soluble and monocyte cell-surface CD163 expression among CTN-055 participants. CD163 is a cell-surface receptor for hemoglobin-haptoglobin complexes that is highly expressed on monocytes and tissue macrophages; upon detection of LPS through cell-surface TLR-4, monocytes and macrophages shed CD163 into plasma (47). Among PLWH, plasma sCD163 levels is considered an indicator of monocyte/macrophage activation, and to reflect underlying intestinal damage (25). Soluble CD163 is also considered a biomarker for liver inflammation and fibrosis among patients with acute and chronic liver disease, particularly individuals with ALD or HCV (48–50). Our study demonstrated increased cell-surface and sCD163 among individuals with AUD both with and without HCV co-infection, and supports findings reported by Monning et al., where alcohol use was also associated with elevated plasma sCD163 among PLWH (51).

Chronic alcohol exposure alters inflammatory responses mediated by TLR-4 through several mechanisms, including alteration in the structure of membrane lipid rafts that support signal transduction through the receptor complex, and in regulatory miRNAs that normally suppress LPS-induced pro-inflammatory immune responses (15, 16, 52). Functionally, chronic alcohol exposure leads to exaggerated pro-inflammatory cytokine responses from monocytes exposed to LPS by altering expression of a key regulatory molecule in the TLR-4 signaling pathway (15). In the current study, although functional responses to LPS were not investigated, cell-surface expression of TLR-4, the receptor essential for innate immune cells to sense LPS from gram-negative bacteria (26), was quantified on host monocytes and found to be more highly expressed among PLWH with AUD. The burden of plasma stimulatory TLR-4 ligands, and LBP, were also significantly increased among individuals with AUD. Paired with our observation of increased soluble and cell-surface CD163 among participants with AUD, and a significant correlation between TLR-4 expression and plasma sCD163 values, our findings suggest that chronic alcohol exposure impacts innate immune activation through TLR-4-mediated pathways.

Our study population allowed insights into interactions between chronic alcohol exposure and HIV/HCV co-infection. Participants with AUD exhibited significantly elevated levels of LBP as compared to PLWH ref. population, confirming a prior report of a significant correlation between liquor consumption and plasma LBP values among PLWH with well controlled viral load (53). HIV/HCV co-infected individuals were noted to have the highest plasma levels of LBP at both study timepoints, however. LBP is acute phase reactant secreted by enterocytes in response to inflammatory stimuli that stimulates the innate inflammatory response by delivering LPS to the TLR-4 receptor complex when present at low concentrations. At high concentrations, however, LBP acts to neutralize LPS and limit potentially harmful inflammatory responses (54, 55). The observed high concentrations of LBP among HIV/HCV co-infected individuals, and significant correlations between plasma LBP and TLR-4 stimulatory activity, offers additional evidence to support a higher LPS burden in this population. Individuals with HIV/HCV co-infection also had higher sCD163 and monocyte TLR-4 expression as compared to HCV- PLWH with and without AUD, as well as a trend towards increased I-FABP values at study enrollment reflecting damaged intestinal epithelium at that timepoint (56). Our findings suggestive of increased gut translocation driving innate immune activation among HIV+/HCV+ as compared to HIV+/HCV- individuals who drink heavily is similar to those reported by Monning et al. Although the plasma metabolome was not assessed as part of the current investigations, an increase in gut translocation among HIV/HCV participants may lead to changes in plasma metabolome, such as altered bile acid profiles, that have subsequent long-term negative impacts on immune homeostasis (57). Our findings of increased immune activation among HIV/HCV co-infected individuals are concerning given that biomarkers of innate immune activation have been linked to progression of liver fibrosis (11–13, 48–50, 58–61). Although overall there was a reduction in the number of self-reported drinking days during the 1-month study intervention, we did not observe any significant changes in the measured indicators of intestinal damage, microbial translocation, or systemic immune activation among CTN-055 participants. Long term studies investigating the impact of alcohol-withdraw among PLWH with and without HCV co-infection are needed to understand if reductions in gut intestinal damage translate to sustained reductions in innate immune activation.

Soluble CD40L is a marker of platelet and immune activation, and has several immunomodulatory effects. CD40L binds to CD40 on the surface of antigen presenting cells such as dendritic cells, to drive activation and subsequent enhancement of the adaptive immune response including T-cell expansion and B-cell immunoglobulin class switching (62). Although elevation in plasma sCD40L during HIV infection has been well documented (34–36, 63), this is the first report of an association between chronic alcohol exposure and elevations in sCD40L among PLWH. We also found that chronic alcohol exposure among PLWH was associated with increases in some plasma cytokines, most notably increased TGF-β1 and TGF-β2, and that there were significant correlations between sCD40L and TGF-β levels. It has been established within the context of HIV-infection that sCD40L can drive differentiation of regulatory T-cells (Tregs) by altering the response of dendritic cells to TLR-mediated priming (35, 36). Although we did not quantify Tregs in this current study, the increase in plasma TGF-β suggests expansion of TGF-β-producing Tregs among PLWH with AUD. Expansion of Tregs could contribute to an imbalance in the ratio between T helper type 17 (Th17) and Tregs, and if this imbalance is maintained within the intestinal lymphoid population, could serve as a mechanism driving microbial translocation and subsequent systemic immune activation (64). It is notable that our analysis illustrated numerous positive correlations between plasma levels of TGF-β1 and TGF-β2, with pro-inflammatory mediators including IL18, IL6, IL9, MCP3, and TNF-α, suggesting a regulatory response to systemic inflammation. Elevation of TGF-β has negative implications for liver health, however, as it plays a role as a profibrogenic mediator known to drive liver fibrosis through activation of hepatic stellate cells (HSCs). TGF-β1 has been shown to modify the extracellular matrix remodeling effects of HSCs through alterations in miRNAs, and also interacts with other cells within the liver, including hepatocytes and hepatic progenitor cells, to drive liver fibrosis (38, 65). Future studies that examine peripheral and mucosal T-cell differentiation, intestinal damage and microbial translocation, and quantify the burden of liver disease within the context of plasma sCD40L and TGF-β, among PLWH with AUD should be prioritized.

Prior publications have suggested that chronic alcohol exposure is associated with expansion of T-cells exhibiting makers of immunosenescence, as well as CD16^+^ non-classical and intermediate populations of monocytes (14, 20, 43). Thus, monocyte and CD4^+^ and CD8^+^ T-cell cellular phenotypes were intensively examined in the current study. Although significant differences in monocyte populations were not noted at study entry, we observed expansion of CD16^+^ non-classical and intermediate monocyte populations at the week 4 timepoint among CTN-055 participants. Classical, CD14^++^CD16^-^ monocytes are critical to the initial immune response to infection, as they are recruited to sites of infection and inflammation to recognize and phagocytose pathogens, secrete pro-inflammatory mediators, and recruit other immune cells (66). CD16^+^ monocytes can be separated into intermediate and non-classical phenotypes based on high versus dim expression of CD14, respectively (31). Although the role of intermediate monocytes remains controversial, non-classical monocytes have been consistently found to provide anti-inflammatory and tissue repair functions due to their capacity to patrol the vascular endothelium and remove microparticles and apoptotic cells. Animal models have suggested that non-classical monocytes are critical to limit infection-induced inflammation (67). Non-classical monocytes have lower level expression of the chemokine receptor CCR2, and are also distinguished by their high expression levels of CX3CR1, the receptor for fractalkine, an essential chemokine that both recruits monocytes and promotes survival (68). It is notable that among participants with AUD, plasma levels of fractalkine were also significantly increased at the week 4 timepoint as compared to our reference population. These findings suggest that the 1-month AUD-intervention was associated with expansion of non-classical monocytes. Given the role of non-classical monocytes in promoting anti-inflammatory responses and tissue repair, expansion of this subset could reduce systemic inflammatory responses among PLWH.

We found that T-cell phenotypes were remarkably similar among all populations and timepoints, likely reflective of well controlled HIV-viremia across all participants. No significant changes in cell-surface markers reflective of CD8^+^ or CD4^+^ T-cell immunosenescence (defined by CD28^-^CD57^+^ T cells), or activation (defined by CD38 and/or HLA-DR expression) were identified. Our findings differ from those of Maffei et al., who reported that alcohol use was associated with expansion of activated-senescent, exhausted, and terminal effector memory CD8^+^ cells among a large cohort of PLWH (20). We did consistently observe increased expression of CD27 among naïve (CD45RA^+^CCR7^+^) and central memory (CD45RA^-^CCR7^+^) CD4^+^ and CD8^+^ T-cells among PLWH with AUD. To our knowledge, there are no prior reports associating chronic alcohol exposure with T-cell CD27 expression. CD27 is a member of the TNF-receptor family expressed by naïve T-cells that serves as a co-stimulatory receptor, and is required to support antigen-specific T-cell expansion as well as long term T-cell memory. CD27 mediated co-stimulation increases both primary, secondary, and memory T-cell responses, and loss of CD27 expression normally occurs after prolonged stimulation as T-cells develop into fully differentiated effector cells. CD27 is believed to be required for efficient survival of virus-specific CD8^+^ T-cells, and to optimize antigen specific CD8^+^ T-cell memory responses by promoting T-cell expression of IL-7Rα (69). In the context of HIV-infection, it has been recognized that HIV-specific CD8^+^ effector T-cells continue to express CD27, with prior reports attributing this finding to a failure of normal T-cell differentiation in the context of HIV-infection (70, 71). However, HIV-specific CD8^+^CD27^+^ T-cells have subsequently been shown to be less prone to apoptosis, generate more IL-2, and to exhibit enhanced proliferative capacity as compared to CD27^-^ HIV-specific CD8^+^ T-cells (72). In the context of CD4^+^ T-cells, data from murine models suggests that CD27 signaling inhibits the induction of Th17 cells and their functional capacity, specifically (73). Although functional studies were not performed here, our findings suggest that there may be underlying differences in antigen-specific CD8^+^ T-cell responses among PLWH with AUD who have well controlled viral loads that should be a priority for future investigations.

Our study has several strengths including inclusion of PLWH with and without HCV co-infection, low-to-undetectable HIV viral loads among study participants that permitted the impact of chronic alcohol exposure to be studied without the potentially confounding effects of HIV-viremia, and the comprehensive nature of the investigations performed. Although the sample size was limited, our statistical approach identified many differences between study cohorts that remained statistically robust after correcting for multiple comparisons. We noted several significant differences in the demographic characteristics between our study cohorts that could potential confound the reported immunologic and inflammatory profiles. Tobacco use, and cigarette smoking specifically, has been associated with HIV progression and non-AIDS morbidities among PLWH (74). Although the impact of nicotine and other components of cigarette smoke on tissue (specifically lung) inflammatory responses has been extensively reported, the impact of cigarette smoking and other methods of nicotine consumption on systemic inflammation and immune responses are more controversial with studies demonstrating both pro- and anti-inflammatory immune effects (75). A recent meta-analysis not specific to PLWH did confirm, however, that tobacco use is independently associated with a small but significance increase in C-reactive protein levels, suggesting that tobacco use is a risk factor for systemic inflammation (76). Tobacco smoke has also been shown to increase systemic levels of LPS (75). Immune cells from PLWH who smoke cigarettes have been shown to exhibit increased evidence for immune senescence as compared to those who do not smoke (77); and among individuals with underlying liver disease, smoking cigarettes has been shown to be an independent risk factor for liver fibrosis (78).

Regarding technical limitations of our investigations, functional responses to LPS were not examined, and we are unable to conclude if AUD is associated with a change in innate responses to TLR-4 stimulation, as we recently reported among PLWH with opioid-use disorder (61). Indirect methods of LPS quantification were utilized, given well-established limitations in accurate direct LPS measurement in plasma (28). Moreover, the reporter cells utilized in the HEK-Blue hTLR4 assay express low levels of endogenous innate receptors (specifically TLR-3 and -5, as well as nucleotide binding oligomerization domain-1 receptor), and therefore some of the observed activation of NF-kB and AP-1 may have been due to plasma ligands specific to these alternative receptors. Assays that more specifically classify microbial translocation (such as plasma 16S rRNA PCR), and examination of the gut microbiome, were not performed. Despite extensive cell-surface phenotyping of participant T-cells, we did not examine T-cell functional responses or expression of transcription factors associated with T-cell differentiation pathways. Therefore, we cannot conclude if the high levels of sCD40L and TGF-β observed among participants with AUD were associated with alterations in the balance of regulatory and Th-17 CD4^+^ T cells. Although longitudinal samples were collected among PLWH with AUD participating in CTN-055 trial, only a 1-month AUD treatment intervention was studied. Studies of prolonged alcohol-detoxification may be necessary to determine if the immunologic changes observed among PLWH and AUD are reversible. Moreover, our investigations should be extended to HIV-uninfected individuals suffering from AUD to determine if our findings are specific to PLWH or generalizable.

In summary, among PLWH, chronic alcohol exposure presents a major modifiable risk factor for chronic immune activation that is well-recognized to predispose individuals to morbidities such as coronary heart disease, stroke, and dementia (79, 80). Long-term studies examining the impact of AUD-treatment on systemic and cellular markers of immune activation, intestinal damage and translocation, and comprehensive immune function should be prioritized among PLWH including those with HCV co-infection.

## Data Availability Statement

The original contributions presented in the study are included in the article/**Supplementary Material**. Further inquiries can be directed to the corresponding author.

## Ethics Statement

The studies involving human participants were reviewed and approved by Oregon Health and Science University IRB. The patients/participants provided their written informed consent to participate in this study.

## Author Contributions

CL and PK contributed to conception and design of the study. CL, MU, LU, GG, SR, LK, PK, RC contributed to assay development and validation, performed and/or processed material for experiments, or were directly involved with participant enrollment and/or collection of essential materials or data. BP performed all statistical analyses. CL, MU, LU, GG, SR, and RPS contributed to writing the manuscript. All authors reviewed the manuscript and approved the submitted version.

## Funding

We would like to thank all study participants for their valuable contribution to this work. The study was funded by the U.S. National Institutes of Health, National Institute on Drug Abuse (NIDA: UG1DA015815, UG1DA013732, R03DA039731 and R01DA046229), the Oregon Clinical & Translational Research Institute, and the Collins Medical Trust.

## Conflict of Interest

The authors declare that the research was conducted in the absence of any commercial or financial relationships that could be construed as a potential conflict of interest.

The reviewer TW declared a shared affiliation with the authors RPS and SR to the handling editor at the time of review.

## Publisher’s Note

All claims expressed in this article are solely those of the authors and do not necessarily represent those of their affiliated organizations, or those of the publisher, the editors and the reviewers. Any product that may be evaluated in this article, or claim that may be made by its manufacturer, is not guaranteed or endorsed by the publisher.

## References

[1] Alcoholism NIoAAa. Available at: https://www.niaaa.nih.gov/publications/brochures-and-fact-sheets/alcohol-facts-and-statistics.

[2] Korthuis PT Fiellin DA McGinnis KA Skanderson M Justice AC Gordon AJ. Unhealthy Alcohol and Illicit Drug Use are Associated With Decreased Quality of HIV Care. J Acquir Immune Defic Syndr (2012) 61(2):171–8. doi: 10.1097/QAI.0b013e31826741aa PMC3460799 22820808

[3] Rehm J Probst C Shield KD Shuper PA. Does Alcohol Use Have a Causal Effect on HIV Incidence and Disease Progression? A Review of the Literature and a Modeling Strategy for Quantifying the Effect. Popul Health Metr (2017) 15(1):4. doi: 10.1186/s12963-017-0121-9 28183309 PMC5301358

[4] Williams EC McGinnis KA Edelman EJ Matson TE Gordon AJ Marshall BDL. Level of Alcohol Use Associated With HIV Care Continuum Targets in a National U.S. Sample of Persons Living With HIV Receiving Healthcare. AIDS Behav (2019) 23(1):140–51. doi: 10.1007/s10461-018-2210-6 PMC6344313 29995206

[5] Crane HM Nance RM Whitney BM Ruderman S Tsui JI Chander G. Drug and Alcohol Use Among People Living With HIV in Care in the United States by Geographic Region. AIDS Care (2021) 33(12):1569–76. doi: 10.1080/09540121.2021.1874274 PMC9104760 33486978

[6] Kahler CW Liu T Cioe PA Bryant V Pinkston MM Kojic EM. Direct and Indirect Effects of Heavy Alcohol Use on Clinical Outcomes in a Longitudinal Study of HIV Patients on ART. AIDS Behav (2017) 21(7):1825–35. doi: 10.1007/s10461-016-1474-y PMC5219952 27392417

[7] Oldfield BJ McGinnis KA Edelman EJ Williams EC Gordon AJ Akgun K. Predictors of Initiation of and Retention on Medications for Alcohol Use Disorder Among People Living With and Without HIV. J Subst Abuse Treat (2020) 109:14–22. doi: 10.1016/j.jsat.2019.11.002 31856946 PMC6982467

[8] Bagby GJ Amedee AM Siggins RW Molina PE Nelson S Veazey RS. Alcohol and HIV Effects on the Immune System. Alcohol Res (2015) 37(2):287–97. 10.35946/arcr.v37.2.12 PMC4590624 26695751

[9] Sandler NG Douek DC. Microbial Translocation in HIV Infection: Causes, Consequences and Treatment Opportunities. Nat Rev Microbiol (2012) 10(9):655–66. doi: 10.1038/nrmicro2848 22886237

[10] Hunt PW Sinclair E Rodriguez B Shive C Clagett B Funderburg N. Gut Epithelial Barrier Dysfunction and Innate Immune Activation Predict Mortality in Treated HIV Infection. J Infect Dis (2014) 210(8):1228–38. doi: 10.1093/infdis/jiu238 PMC4192038 24755434

[11] French AL Evans CT Agniel DM Cohen MH Peters M Landay AL. Microbial Translocation and Liver Disease Progression in Women Coinfected With HIV and Hepatitis C Virus. J Infect Dis (2013) 208(4):679–89. doi: 10.1093/infdis/jit225 PMC3719907 23687224

[12] Osna NA Ganesan M Kharbanda KK. Hepatitis C, Innate Immunity and Alcohol: Friends or Foes? Biomolecules (2015) 5(1):76–94. doi: 10.3390/biom5010076 25664450 PMC4384112

[13] Shmagel KV Saidakova EV Shmagel NG Korolevskaya LB Chereshnev VA Robinson J. Systemic Inflammation and Liver Damage in HIV/hepatitis C Virus Coinfection. HIV Med (2016) 17(8):581–9. doi: 10.1111/hiv.12357 PMC4987156 27187749

[14] Donnadieu-Rigole H Mura T Portales P Duroux-Richard I Bouthier M Eliaou JF. Effects of Alcohol Withdrawal on Monocyte Subset Defects in Chronic Alcohol Users. J Leukoc Biol (2016) 100(5):1191–9. doi: 10.1189/jlb.5A0216-060RR 27256567

[15] Mandrekar P Bala S Catalano D Kodys K Szabo G. The Opposite Effects of Acute and Chronic Alcohol on Lipopolysaccharide-Induced Inflammation Are Linked to IRAK-M in Human Monocytes. J Immunol (2009) 183(2):1320–7. doi: 10.4049/jimmunol.0803206 PMC3845821 19561104

[16] Szabo G Mandrekar P Petrasek J Catalano D. The Unfolding Web of Innate Immune Dysregulation in Alcoholic Liver Injury. Alcohol Clin Exp Res (2011) 35(5):782–6. doi: 10.1111/j.1530-0277.2010.01398.x PMC3742381 21284666

[17] Szabo G Saha B. Alcohol's Effect on Host Defense. Alcohol Res (2015) 37(2):159–70. 10.35946/arcr.v37.2.01 PMC4590613 26695755

[18] Alzahrani J Hussain T Simar D Palchaudhuri R Abdel-Mohsen M Crowe SM. Inflammatory and Immunometabolic Consequences of Gut Dysfunction in HIV: Parallels With IBD and Implications for Reservoir Persistence and non-AIDS Comorbidities. EBioMedicine (2019) 46:522–31. doi: 10.1016/j.ebiom.2019.07.027 PMC6710907 31327693

[19] Engen PA Green SJ Voigt RM Forsyth CB Keshavarzian A. The Gastrointestinal Microbiome: Alcohol Effects on the Composition of Intestinal Microbiota. Alcohol Res (2015) 37(2):223–36. 10.35946/arcr.v37.2.07 PMC4590619 26695747

[20] Maffei VJ Siggins RW Luo M Brashear MM Mercante DE Taylor CM. Alcohol Use Is Associated With Intestinal Dysbiosis and Dysfunctional CD8+ T-Cell Phenotypes in Persons With Human Immunodeficiency Virus. J Infect Dis (2021) 223(6):1029–39. doi: 10.1093/infdis/jiaa461 PMC8006423 32725203

[21] Korthuis PT Lum PJ Vergara-Rodriguez P Ahamad K Wood E Kunkel LE. Feasibility and Safety of Extended-Release Naltrexone Treatment of Opioid and Alcohol Use Disorder in HIV Clinics: A Pilot/Feasibility Randomized Trial. Addiction (2017) 112(6):1036–44. doi: 10.1111/add.13753 PMC5408318 28061017

[22] Olkin I. Contributions to Probability and Statistics: Essays in Honor of Harold Hotelling. Stanford, CA: Stanford University Press (1960).

[23] Steel RGD. A Rank SUm Test for Comparing All Pairs O Treatments. Technometrics (1960) 2:197–207. doi: 10.1080/00401706.1960.10489894

[24] Critchlow DE Fligner MA. On Distribution-Free Multiple Comparisons in the One-Way Analysis of Variance. Commun Statistics: Theory Methods (1991) 20):127–39. doi: 10.1080/03610929108830487

[25] Burdo TH Lentz MR Autissier P Krishnan A Halpern E Letendre S. Soluble CD163 Made by Monocyte/Macrophages is a Novel Marker of HIV Activity in Early and Chronic Infection Prior to and After Anti-Retroviral Therapy. J Infect Dis (2011) 204(1):154–63. doi: 10.1093/infdis/jir214 PMC3105035 21628670

[26] Molteni M Gemma S Rossetti C. The Role of Toll-Like Receptor 4 in Infectious and Noninfectious Inflammation. Mediators Inflammation (2016) 2016:6978936. doi: 10.1155/2016/6978936 PMC4887650 27293318

[27] Ciesielska A Matyjek M Kwiatkowska K. TLR4 and CD14 Trafficking and its Influence on LPS-Induced Pro-Inflammatory Signaling. Cell Mol Life Sci (2021) 78(4):1233–61. doi: 10.1007/s00018-020-03656-y PMC7904555 33057840

[28] Munford RS. Endotoxemia-Menace, Marker, or Mistake? J Leukoc Biol (2016) 100(4):687–98. doi: 10.1189/jlb.3RU0316-151R PMC5014740 27418356

[29] Ellery PJ Tippett E Chiu YL Paukovics G Cameron PU Solomon A. The CD16+ Monocyte Subset is More Permissive to Infection and Preferentially Harbors HIV-1 *In Vivo* . J Immunol (2007) 178(10):6581–9. doi: 10.4049/jimmunol.178.10.6581 17475889

[30] Ancuta P Kunstman KJ Autissier P Zaman T Stone D Wolinsky SM. CD16+ Monocytes Exposed to HIV Promote Highly Efficient Viral Replication Upon Differentiation Into Macrophages and Interaction With T Cells. Virology (2006) 344(2):267–76. doi: 10.1016/j.virol.2005.10.027 16305804

[31] Ziegler-Heitbrock L Ancuta P Crowe S Dalod M Grau V Hart DN. Nomenclature of Monocytes and Dendritic Cells in Blood. Blood (2010) 116(16):e74–80. doi: 10.1182/blood-2010-02-258558 20628149

[32] van Kooten C Banchereau J. CD40-CD40 Ligand. J Leukoc Biol (2000) 67(1):2–17. doi: 10.1002/jlb.67.1.2 10647992

[33] Aloui C Prigent A Sut C Tariket S Hamzeh-Cognasse H Pozzetto B. The Signaling Role of CD40 Ligand in Platelet Biology and in Platelet Component Transfusion. Int J Mol Sci (2014) 15(12):22342–64. doi: 10.3390/ijms151222342 PMC4284712 25479079

[34] Davidson DC Jackson JW Maggirwar SB. Targeting Platelet-Derived Soluble CD40 Ligand: A New Treatment Strategy for HIV-Associated Neuroinflammation? J Neuroinflamm (2013) 10:144. doi: 10.1186/1742-2094-10-144 PMC3906985 24289660

[35] Jenabian MA Patel M Kema I Vyboh K Kanagaratham C Radzioch D. Soluble CD40-Ligand (Scd40l, Scd154) Plays an Immunosuppressive Role *via* Regulatory T Cell Expansion in HIV Infection. Clin Exp Immunol (2014) 178(1):102–11. doi: 10.1111/cei.12396 PMC4360200 24924152

[36] Miller EA Gopal R Valdes V Berger JS Bhardwaj N O'Brien MP. Soluble CD40 Ligand Contributes to Dendritic Cell-Mediated T-Cell Dysfunction in HIV-1 Infection. AIDS (2015) 29(11):1287–96. doi: 10.1097/QAD.0000000000000698 PMC4478195 26091297

[37] Li MO Flavell RA. TGF-Beta: A Master of All T Cell Trades. Cell (2008) 134(3):392–404. doi: 10.1016/j.cell.2008.07.025 18692464 PMC3677783

[38] Gaitantzi H Meyer C Rakoczy P Thomas M Wahl K Wandrer F. Ethanol Sensitizes Hepatocytes for TGF-Beta-Triggered Apoptosis. Cell Death Dis (2018) 9(2):51. doi: 10.1038/s41419-017-0071-y 29352207 PMC5833779

[39] Adams C Conigrave JH Lewohl J Haber P Morley KC. Alcohol Use Disorder and Circulating Cytokines: A Systematic Review and Meta-Analysis. Brain Behav Immun (2020) 89:501–12. doi: 10.1016/j.bbi.2020.08.002 32805393

[40] Asquith M Pasala S Engelmann F Haberthur K Meyer C Park B. Chronic Ethanol Consumption Modulates Growth Factor Release, Mucosal Cytokine Production, and microRNA Expression in Nonhuman Primates. Alcohol Clin Exp Res (2014) 38(4):980–93. doi: 10.1111/acer.12325 PMC3984381 24329418

[41] Bishehsari F Magno E Swanson G Desai V Voigt RM Forsyth CB. Alcohol and Gut-Derived Inflammation. Alcohol Res (2017) 38(2):163–71. 10.35946/arcr.v38.2.02 PMC5513683 28988571

[42] Donnadieu-Rigole H Pansu N Mura T Pelletier S Alarcon R Gamon L. Beneficial Effect of Alcohol Withdrawal on Gut Permeability and Microbial Translocation in Patients With Alcohol Use Disorder. Alcohol Clin Exp Res (2018) 42(1):32–40. doi: 10.1111/acer.13527 29030980

[43] Katz PS Siggins RW Porretta C Armstrong ML Zea AH Mercante DE. Chronic Alcohol Increases CD8+ T-Cell Immunosenescence in Simian Immunodeficiency Virus-Infected Rhesus Macaques. Alcohol (2015) 49(8):759–65. doi: 10.1016/j.alcohol.2015.09.003 PMC4691397 26603633

[44] Sturm R Haag F Janicova A Xu B Vollrath JT Bundkirchen K. Acute Alcohol Consumption Increases Systemic Endotoxin Bioactivity for Days in Healthy Volunteers-With Reduced Intestinal Barrier Loss in Female. Eur J Trauma Emerg Surg (2021). doi: 10.1007/s00068-021-01666-4 PMC9192383 33839799

[45] Sureshchandra S Raus A Jankeel A Ligh BJK Walter NAR Newman N. Dose-Dependent Effects of Chronic Alcohol Drinking on Peripheral Immune Responses. Sci Rep (2019) 9(1):7847. doi: 10.1038/s41598-019-44302-3 31127176 PMC6534547

[46] Ancona G Merlini E Tincati C Barassi A Calcagno A Augello M. Long-Term Suppressive cART Is Not Sufficient to Restore Intestinal Permeability and Gut Microbiota Compositional Changes. Front Immunol (2021) 12:639291. doi: 10.3389/fimmu.2021.639291 33717191 PMC7952451

[47] Weaver LK Pioli PA Wardwell K Vogel SN Guyre PM. Up-Regulation of Human Monocyte CD163 Upon Activation of Cell-Surface Toll-Like Receptors. J Leukoc Biol (2007) 81(3):663–71. doi: 10.1189/jlb.0706428 17164428

[48] Gronbaek H Sandahl TD Mortensen C Vilstrup H Moller HJ Moller S. Soluble CD163, a Marker of Kupffer Cell Activation, is Related to Portal Hypertension in Patients With Liver Cirrhosis. Aliment Pharmacol Ther (2012) 36(2):173–80. doi: 10.1111/j.1365-2036.2012.05134.x 22591184

[49] Kazankov K Barrera F Moller HJ Bibby BM Vilstrup H George J. Soluble CD163, a Macrophage Activation Marker, Is Independently Associated With Fibrosis in Patients With Chronic Viral Hepatitis B and C. Hepatology (2014) 60(2):521–30. doi: 10.1002/hep.27129 24623375

[50] Nielsen MC Hvidbjerg Gantzel R Claria J Trebicka J Moller HJ Gronbaek H. Macrophage Activation Markers, CD163 and CD206, in Acute-On-Chronic Liver Failure. Cells (2020) 9(5):1175. doi: 10.3390/cells9051175 PMC7290463 32397365

[51] Monnig MA Cohen R Ramratnam B McAdams M Tashima K Monti PM. HIV Infection, HCV Coinfection, and Alcohol Use: Associations With Microbial Translocation and Immune Activation. Alcohol Clin Exp Res (2019) 43(6):1126–34. doi: 10.1111/acer.14032 PMC6551270 30908642

[52] Szabo G Dolganiuc A Dai Q Pruett SB. TLR4, Ethanol, and Lipid Rafts: A New Mechanism of Ethanol Action With Implications for Other Receptor-Mediated Effects. J Immunol (2007) 178(3):1243–9. doi: 10.4049/jimmunol.178.3.1243 17237368

[53] Webel AR Sattar A Funderburg NT Kinley B Longenecker CT Labbato D. Alcohol and Dietary Factors Associate With Gut Integrity and Inflammation in HIV-Infected Adults. HIV Med (2017) 18(6):402–11. doi: 10.1111/hiv.12442 PMC5584535 27860212

[54] Zweigner J Gramm HJ Singer OC Wegscheider K Schumann RR. High Concentrations of Lipopolysaccharide-Binding Protein in Serum of Patients With Severe Sepsis or Septic Shock Inhibit the Lipopolysaccharide Response in Human Monocytes. Blood (2001) 98(13):3800–8. doi: 10.1182/blood.v98.13.3800 11739189

[55] Zweigner J Schumann RR Weber JR. The Role of Lipopolysaccharide-Binding Protein in Modulating the Innate Immune Response. Microbes Infect (2006) 8(3):946–52. doi: 10.1016/j.micinf.2005.10.006 16483818

[56] Xu H Diolintzi A Storch J. Fatty Acid-Binding Proteins: Functional Understanding and Diagnostic Implications. Curr Opin Clin Nutr Metab Care (2019) 22(6):407–12. doi: 10.1097/MCO.0000000000000600 PMC9940447 31503024

[57] Shukla S Kumari S Bal SK Monaco DC Ribeiro SP Sekaly RP. "Go", "No Go," or "Where to Go"; Does Microbiota Dictate T Cell Exhaustion, Programming, and HIV Persistence? Curr Opin HIV AIDS (2021) 16(4):215–22. doi: 10.1097/COH.0000000000000692 PMC11309580 34039845

[58] Liaskou E Zimmermann HW Li KK Oo YH Suresh S Stamataki Z. Monocyte Subsets in Human Liver Disease Show Distinct Phenotypic and Functional Characteristics. Hepatology (2013) 57(1):385–98. doi: 10.1002/hep.26016 PMC4194426 22911542

[59] Reid M Ma Y Scherzer R Price JC French AL Huhn GD. Contribution of Liver Fibrosis and Microbial Translocation to Immune Activation in Persons Infected With HIV and/or Hepatitis C Virus. J Infect Dis (2018) 217(8):1289–97. doi: 10.1093/infdis/jix688 PMC6019002 29304196

[60] Underwood ML Nguyen T Merrifield M Korthuis T Lancioni C. Immune Dysregulation Among HIV-Infected Individuals With Opioid-Use Disorders. Coll Problems Drug Depend (2018). San Diego, USA.

[61] Underwood ML Nguyen T Uebelhoer LS Kunkel LE Korthuis PT Lancioni CL. Altered Monocyte Phenotype and Dysregulated Innate Cytokine Responses Among People Living With HIV and Opioid-Use Disorder. AIDS (2020) 34(2):177–88. doi: 10.1097/QAD.0000000000002416 PMC6948804 31687981

[62] Klaus SJ Berberich I Shu G Clark EA. CD40 and its Ligand in the Regulation of Humoral Immunity. Semin Immunol (1994) 6(5):279–86. doi: 10.1006/smim.1994.1036 7532458

[63] Donhauser N Pritschet K Helm M Harrer T Schuster P Ries M. Chronic Immune Activation in HIV-1 Infection Contributes to Reduced Interferon Alpha Production *via* Enhanced CD40:CD40 Ligand Interaction. PloS One (2012) 7(3):e33925. doi: 10.1371/journal.pone.0033925 22470494 PMC3309969

[64] Brenchley JM Douek DC. Microbial Translocation Across the GI Tract. Annu Rev Immunol (2012) 30:149–73. doi: 10.1146/annurev-immunol-020711-075001 PMC3513328 22224779

[65] Fabregat I Moreno-Caceres J Sanchez A Dooley S Dewidar B Giannelli G. TGF-Beta Signalling and Liver Disease. FEBS J (2016) 283(12):2219–32. doi: 10.1111/febs.13665 26807763

[66] Narasimhan PB Marcovecchio P Hamers AAJ Hedrick CC. Nonclassical Monocytes in Health and Disease. Annu Rev Immunol (2019) 37:439–56. doi: 10.1146/annurev-immunol-042617-053119 31026415

[67] Alkhani A Levy CS Tsui M Rosenberg KA Polovina K Mattis AN. Ly6c(Lo) non-Classical Monocytes Promote Resolution of Rhesus Rotavirus-Mediated Perinatal Hepatic Inflammation. Sci Rep (2020) 10(1):7165. doi: 10.1038/s41598-020-64158-2 32346042 PMC7188847

[68] Thomas G Tacke R Hedrick CC Hanna RN. Nonclassical Patrolling Monocyte Function in the Vasculature. Arterioscler Thromb Vasc Biol (2015) 35(6):1306–16. doi: 10.1161/ATVBAHA.114.304650 PMC4441550 25838429

[69] Grant EJ Nussing S Sant S Clemens EB Kedzierska K. The Role of CD27 in Anti-Viral T-Cell Immunity. Curr Opin Virol (2017) 22:77–88. doi: 10.1016/j.coviro.2016.12.001 28086150

[70] Appay V Dunbar PR Callan M Klenerman P Gillespie GM Papagno L. Memory CD8+ T Cells Vary in Differentiation Phenotype in Different Persistent Virus Infections. Nat Med (2002) 8(4):379–85. doi: 10.1038/nm0402-379 11927944

[71] van Baarle D Kostense S Hovenkamp E Ogg G Nanlohy N Callan MF. Lack of Epstein-Barr Virus- and HIV-Specific CD27- CD8+ T Cells Is Associated With Progression to Viral Disease in HIV-Infection. AIDS (2002) 16(15):2001–11. doi: 10.1097/00002030-200210180-00004 12370498

[72] Ochsenbein AF Riddell SR Brown M Corey L Baerlocher GM Lansdorp PM. CD27 Expression Promotes Long-Term Survival of Functional Effector-Memory CD8+ Cytotoxic T Lymphocytes in HIV-Infected Patients. J Exp Med (2004) 200(11):1407–17. doi: 10.1084/jem.20040717 PMC2211945 15583014

[73] Coquet JM Ribot JC Babala N Middendorp S van der Horst G Xiao Y. Epithelial and Dendritic Cells in the Thymic Medulla Promote CD4+Foxp3+ Regulatory T Cell Development *via* the CD27-CD70 Pathway. J Exp Med (2013) 210(4):715–28. doi: 10.1084/jem.20112061 PMC3620350 23547099

[74] Shirley DK Kaner RJ Glesby MJ. Effects of Smoking on non-AIDS-Related Morbidity in HIV-Infected Patients. Clin Infect Dis (2013) 57(2):275–82. doi: 10.1093/cid/cit207 PMC3689343 23572487

[75] Arnson Y Shoenfeld Y Amital H. Effects of Tobacco Smoke on Immunity, Inflammation and Autoimmunity. J Autoimmun (2010) 34(3):J258–65. doi: 10.1016/j.jaut.2009.12.003 20042314

[76] Doggui R Elsawy W Conti AA Baldacchino A. Association Between Chronic Psychoactive Substances Use and Systemic Inflammation: A Systematic Review and Meta-Analysis. Neurosci Biobehav Rev (2021) 125:208–20. doi: 10.1016/j.neubiorev.2021.02.031 33639179

[77] Calvo M Laguno M Martinez M Martinez E. Effects of Tobacco Smoking on HIV-Infected Individuals. AIDS Rev (2015) 17(1):47–55. 25427101

[78] Premkumar M Anand AC. Tobacco, Cigarettes, and the Liver: The Smoking Gun. J Clin Exp Hepatol (2021) 11(6):700–12. doi: 10.1016/j.jceh.2021.07.016 PMC8617531 34866849

[79] Anzinger JJ Butterfield TR Angelovich TA Crowe SM Palmer CS. Monocytes as Regulators of Inflammation and HIV-Related Comorbidities During cART. J Immunol Res (2014) 2014:569819. doi: 10.1155/2014/569819 25025081 PMC4082935

[80] Wilson EM Singh A Hullsiek KH Gibson D Henry WK Lichtenstein K. Monocyte-Activation Phenotypes Are Associated With Biomarkers of Inflammation and Coagulation in Chronic HIV Infection. J Infect Dis (2014) 210(9):1396–406. doi: 10.1093/infdis/jiu275 PMC4207864 24813472

