# Supplementary material for: Chronic Alcohol Exposure Among People Living with HIV Is Associated with Innate Immune Activation and Alterations in Monocyte Phenotype and Plasma Cytokine Profile
Source: Front Immunol. 2022 Mar 18;13:867937. doi: 10.3389/fimmu.2022.867937 (PMC8971672; doi:10.3389/fimmu.2022.867937)
Supplement: Supplementary file 1 [file DataSheet_1.pdf]

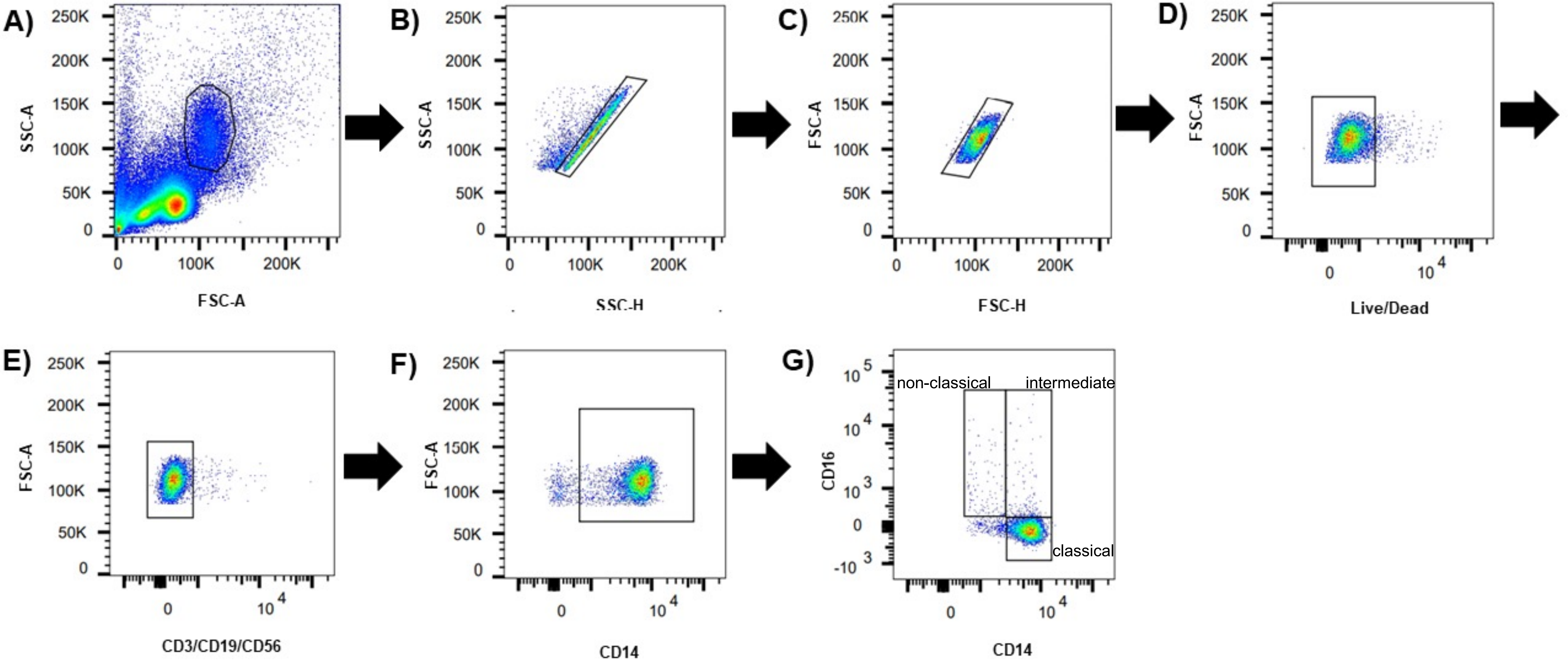

Supplemental Figure 1: Flow cytometry gating strategy to identify classical (CD14++CD16-), intermediate (CD14++CD16+) and non-classical ($CD14^{dim}CD16+$) monocytes in PBMC. A wide gate was drawn around large cells with monocyte characteristics using forward-scatter and side-scatter parameters (A). Doublet discrimination was performed using SSC-A and SSC-H (B), followed by FSC-A and FSC-H (C). Live cells were selected (D), and then cells expressing CD3, CD19 or CD56 were excluded to prevent T cells, B cells and NK cell contamination in the analysis (E). CD14+ monocytes were selected (F) and further analyzed based on expression of CD14 and CD16 (G). Fluorescence minus one controls were used to draw gates on positive or negative populations (not shown).

Supplemental Table 1. Comparison of immune phenotypes and cytokine responses among CTN-055 CHOICES participants with and without hepatitis C virus (HCV), and PLWH ref. group

Cell Phenotype				Comparisons between Cohorts			
		**Lower 95% CI (unadjusted)**	**Upper 95% CI (unadjusted)**	**p-value (unadjusted)**	**Lower 95% CI (adjusted)**	**Upper 95% CI (adjusted)**	**p-value (adjusted)**
Total CD14+ CCR2 mid	W0: AUD+ vs PLWH Ref.	-2.1992	3.3958	0.6704	-2.7624	3.9589	0.9043
	W4: AUD+ vs PLWH Ref.	0.1757	5.8434	**0.0378**	-0.3948	6.4138	0.0934
	W0-W4: AUD+ only	-5.3072	0.4847	0.1010	-5.8901	1.0676	0.2269
Total CD14+ CCR2 bright	W0: AUD+/HCV+ vs PLWH Ref.	-6.1120	2.9831	0.4938	-7.9606	4.8318	0.9582
	W0: AUD+/HCV- vs PLWH Ref.	-4.0500	3.1155	0.7950	-5.5064	4.5719	0.9989
	W4: AUD+/HCV+ vs PLWH Ref.	-4.9305	4.1646	0.8667	-6.7792	6.0132	0.9998
	W4: AUD+/HCV- vs PLWH Ref.	-9.0271	-1.6951	**0.0049**	-10.5174	-0.2049	**0.0377**
	W0-W4: AUD+/HCV+	-6.7829	4.4201	0.6744	-9.0600	6.6972	0.9932
	W0-W4: AUD+/HCV-	0.8575	8.9302	**0.0184**	-0.7833	10.5710	0.1227
Total CD14+ CD163+	W0: AUD+ vs PLWH Ref.	2.5453	17.8704	**0.0099**	1.0028	19.4129	**0.0263**
	W4: AUD+ vs PLWH Ref.	2.1353	17.6594	**0.0133**	0.5728	19.2220	**0.0350**
	W0-W4: AUD+ only	-7.6216	8.2426	0.9379	-9.2184	9.8393	0.9966
Total CD14+ PD-1+	W0: AUD+ vs PLWH Ref.	-0.0060	0.3896	0.0571	-0.0458	0.4294	0.1365
	W4: AUD+ vs PLWH Ref.	-0.0667	0.3340	0.1872	-0.1070	0.3743	0.3821
	W0-W4: AUD+ only	-0.1466	0.2629	0.5722	-0.1878	0.3041	0.8376
Total CD14+ PD-1L+	W0: AUD+ vs PLWH Ref.	-1.8548	1.7798	0.9672	-2.2207	2.1456	0.9991
	W4: AUD+ vs PLWH Ref.	-2.0645	1.6173	0.8089	-2.4351	1.9879	0.9680

	W0-W4: AUD+ only	-1.6952	2.0673	0.8439	-2.0739	2.4460	0.9787
Total CD14+ TLR4+	W0: AUD+/HCV+ vs PLWH Ref.	8.7251	17.4272	**0.0000**	6.9563	19.1960	**0.0000**
	W0: AUD+/HCV- vs PLWH Ref.	2.7268	9.5827	**0.0007**	1.3333	10.9762	**0.0058**
	W4: AUD+/HCV+ vs PLWH Ref.	4.0251	12.7272	**0.0003**	2.2563	14.4960	**0.0026**
	W4: AUD+/HCV- vs PLWH Ref.	2.3452	9.3604	**0.0015**	0.9193	10.7862	**0.0123**
	W0-W4: AUD+/HCV+	-0.6595	10.0595	0.0845	-2.8382	12.2382	0.4091
	W0-W4: AUD+/HCV-	-3.5600	4.1639	0.8762	-5.1299	5.7338	0.9999
Total CD14+ HLA-DR (Geo Mean)	W0: AUD+ vs PLWH Ref.	-88.7112	2215.9500	0.0698	-320.6800	2447.9100	0.1635
	W4: AUD+ vs PLWH Ref.	-368.1200	1966.4600	0.1760	-603.1000	2201.4400	0.3634
	W0-W4: AUD+ only	-928.4200	1457.3100	0.6591	-1168.5500	1697.4400	0.8975
Intermediate Monocytes (CD14++CD16+)	W0: AUD+ vs PLWH Ref.	-0.0818	14.1103	**0.0526**	-1.5102	15.5388	0.1267
	W4: AUD+ vs PLWH Ref.	7.1545	21.5309	**0.0002**	5.7075	22.9779	**0.0005**
	W0-W4: AUD+ only	-14.6741	0.0172	**0.0505**	-16.1528	1.4959	0.1221
Intermediate monocytes (CD14++CD16+) CCR2+	W0: AUD+ vs PLWH Ref.	0.0384	1.4851	**0.0394**	-0.1073	1.6307	0.0971
	W4: AUD+ vs PLWH Ref.	0.0144	1.4799	**0.0458**	-0.1331	1.6274	0.1116
	W0-W4: AUD+ only	-0.7343	0.7633	0.9692	-0.8850	0.9141	0.9992
Intermediate monocytes (CD14++CD16+) CCR2 mid	W0: AUD+ vs PLWH Ref.	-7.5917	3.3659	0.4436	-8.6946	4.4689	0.7220
	W4: AUD+ vs PLWH Ref.	-4.4812	6.6187	0.7015	-5.5985	7.7359	0.9216
	W0-W4: AUD+ only	-8.8531	2.4900	0.2664	-9.9948	3.6317	0.5045
Intermediate monocytes (CD14++CD16+) CCR2 bright	W0: AUD+ vs PLWH Ref.	-3.0884	8.8375	0.3389	-4.2888	10.0379	0.6023
	W4: AUD+ vs PLWH Ref.	-6.3619	5.7189	0.9156	-7.5779	6.9348	0.9938

	W0-W4: AUD+ only	-2.9766	9.3688	0.3046	-4.2192	10.6114	0.5576
Intermediate monocytes (CD14++CD16+) CD163+	W0: AUD+ vs PLWH Ref.	0.4258	13.2024	**0.0370**	-0.8602	14.4884	0.0916
	W4: AUD+ vs PLWH Ref.	-0.3191	12.6234	0.0620	-1.6218	13.9261	0.1471
	W0-W4: AUD+ only	-5.9511	7.2749	0.8420	-7.2824	8.6062	0.9782
Intermediate monocytes (CD14++CD16+) PD-1+	W0: AUD+ vs PLWH Ref.	0.0043	1.9331	**0.0490**	-0.1898	2.1272	0.1187
	W4: AUD+ vs PLWH Ref.	-0.6849	1.2689	0.5523	-0.8816	1.4655	0.8219
	W0-W4: AUD+ only	-0.3216	1.6750	0.1802	-0.5225	1.8760	0.3705
Intermediate monocytes (CD14++CD16+) PD-1L+	W0: AUD+ vs PLWH Ref.	-5.9464	3.3829	0.5847	-6.8854	4.3219	0.8471
	W4: AUD+ vs PLWH Ref.	-8.3107	1.1398	0.1344	-9.2619	2.0910	0.2900
	W0-W4: AUD+ only	-2.5250	7.1325	0.3438	-3.4970	8.1045	0.6085
Intermediate monocytes (CD14++CD16+) TLR4+	W0: AUD+/HCV+ vs PLWH Ref.	1.5226	22.6471	**0.0257**	-2.7711	26.9408	0.1628
	W0: AUD+/HCV- vs PLWH Ref.	-6.2915	10.3512	0.6272	-9.6742	13.7340	0.9882
	W4: AUD+/HCV+ vs PLWH Ref.	-5.5488	15.5757	0.3460	-9.8425	19.8694	0.8759
	W4: AUD+/HCV- vs PLWH Ref.	-6.1254	10.9040	0.5765	-9.5868	14.3653	0.9800
	W0-W4: AUD+/HCV+	-5.9388	20.0816	0.2811	-11.2276	25.3704	0.8120
	W0-W4: AUD+/HCV-	-9.7342	9.0155	0.9391	-13.5452	12.8264	1.0000
Intermediate monocytes (CD14++CD16+) HLA-DR Geo Mean	W0: AUD+/HCV+ vs PLWH Ref.	36.4319	4563.5900	**0.0465**	-883.7400	5483.7700	0.2632
	W0: AUD+/HCV- vs PLWH Ref.	-1581.6800	1984.9900	0.8217	-2306.6300	2709.9400	0.9994

	W4: AUD+/HCV+ vs PLWH Ref.	-1329.7100	3197.4500	0.4123	-2249.8900	4117.6300	0.9216
	W4: AUD+/HCV- vs PLWH Ref.	-895.1200	2754.4200	0.3121	-1636.9100	3496.2100	0.8452
	W0-W4: AUD+/HCV+	-1422.0500	4154.3400	0.3308	-2555.4900	5287.7800	0.8628
	W0-W4: AUD+/HCV-	-2737.1000	1281.1200	0.4712	-3553.8300	2097.8400	0.9498
Classical Monocytes (CD14++CD16-)	W0: AUD+ vs PLWH Ref.	-14.0688	0.0328	**0.0510**	-15.4882	1.4521	0.1232
	W4: AUD+ vs PLWH Ref.	-21.6782	-7.3935	**0.0001**	-23.1160	-5.9558	**0.0004**
	W0-W4: AUD+ only	0.2190	14.8167	**0.0437**	-1.2502	16.2860	0.1069
Classical Monocytes (CD14++CD16-) CCR2+	W0: AUD+ vs PLWH Ref.	-0.0429	0.1779	0.2263	-0.0652	0.2001	0.4448
	W4: AUD+ vs PLWH Ref.	-0.1355	0.0882	0.6733	-0.1581	0.1107	0.9060
	W0-W4: AUD+ only	-0.0231	0.2055	0.1158	-0.0461	0.2285	0.2554
Classical Monocytes (CD14++CD16-) CCR2 mids	W0: AUD+ vs PLWH Ref.	-2.2636	1.0869	0.4852	-2.6009	1.4241	0.7630
	W4: AUD+ vs PLWH Ref.	-1.2435	2.1505	0.5950	-1.5851	2.4922	0.8547
	W0-W4: AUD+ only	-2.7761	0.6923	0.2342	-3.1252	1.0414	0.4569
Classical Monocytes (CD14++CD16-) CCR2 bright	W0: AUD+ vs PLWH Ref.	-1.0988	2.4105	0.4577	-1.4520	2.7638	0.7363
	W4: AUD+ vs PLWH Ref.	-2.2547	1.3002	0.5933	-2.6125	1.6580	0.8535
	W0-W4: AUD+ only	-0.6833	2.9495	0.2170	-1.0489	3.3152	0.4303
Classical Monocytes (CD14++CD16-) CD163+	W0: AUD+ vs PLWH Ref.	1.0977	14.6237	**0.0235**	-0.2638	15.9851	0.0599
	W4: AUD+ vs PLWH Ref.	-1.2678	12.4339	0.1083	-2.6469	13.8130	0.2411
	W0-W4: AUD+ only	-4.7233	9.2785	0.5178	-6.1326	10.6878	0.7928
Classical Monocytes (CD14++CD16-) PD-1+	W0: AUD+ vs PLWH Ref.	-0.1320	0.3238	0.4032	-0.1778	0.3697	0.6787
	W4: AUD+ vs PLWH Ref.	-0.1081	0.3535	0.2920	-0.1546	0.4000	0.5405
	W0-W4: AUD+ only	-0.2627	0.2091	0.8212	-0.3102	0.2566	0.9720
	W0: AUD+ vs PLWH Ref.	-1.7520	1.6743	0.9640	-2.0969	2.0192	0.9989

Classical Monocytes (CD14++CD16-) PD-1L+	W4: AUD+ vs PLWH Ref.	-1.8436	1.6272	0.9012	-2.1929	1.9766	0.9915
	W0-W4: AUD+ only	-1.7041	1.8428	0.9379	-2.0611	2.1998	0.9966
Classical Monocytes (CD14++CD16-) TLR4+	W0: AUD+/HCV+ vs PLWH Ref.	9.7282	21.0021	**0.0000**	7.4367	23.2936	**0.0000**
	W0: AUD+/HCV- vs PLWH Ref.	2.0934	10.9754	**0.0046**	0.2881	12.7808	**0.0360**
	W4: AUD+/HCV+ vs PLWH Ref.	4.4953	15.7692	**0.0007**	2.2038	18.0607	**0.0058**
	W4: AUD+/HCV- vs PLWH Ref.	1.9627	11.0511	**0.0058**	0.1154	12.8984	**0.0440**
	W0-W4: AUD+/HCV+	-1.7105	12.1763	0.1368	-4.5331	14.9988	0.5612
	W0-W4: AUD+/HCV-	-4.9757	5.0308	0.9913	-7.0096	7.0647	1.0000
Classical Monocytes (CD14++CD16-) HLA-DR Geo Mean	W0: AUD+ vs PLWH Ref.	-271.9200	1919.0300	0.1379	-492.4500	2139.5600	0.2965
	W4: AUD+ vs PLWH Ref.	-659.0300	1560.3800	0.4199	-882.4200	1783.7600	0.6970
	W0-W4: AUD+ only	-761.1300	1506.9000	0.5133	-989.4200	1735.1800	0.7888
Non-classical Monocytes ($CD14^{dim}CD16+$)	W0: AUD+/HCV+ vs PLWH Ref.	-0.1094	2.5497	0.0713	-0.6499	3.0902	0.3628
	W0: AUD+/HCV- vs PLWH Ref.	0.2334	2.3283	**0.0174**	-0.1925	2.7542	0.1173
	W4: AUD+/HCV+ vs PLWH Ref.	-0.1437	2.5154	0.0794	-0.6842	3.0559	0.3918
	W4: AUD+/HCV- vs PLWH Ref.	1.7939	3.9376	**0.0000**	1.3582	4.3733	**0.0000**
	W0-W4: AUD+/HCV+	-1.6034	1.6720	0.9667	-2.2692	2.3378	1.0000
	W0-W4: AUD+/HCV-	-2.7650	-0.4048	**0.0094**	-3.2447	0.0749	0.0680

Non-classical Monocytes ($CD14^{dim}$CD16+) CCR2+	W0: AUD+ vs PLWH Ref.	-2.4630	13.4515	0.1724	-4.0649	15.0533	0.3573
	W4: AUD+ vs PLWH Ref.	-1.2047	14.9164	0.0941	-2.8273	16.5391	0.2132
	W0-W4: AUD+ only	-9.5988	6.8755	0.7421	-11.2570	8.5337	0.9416
Non-classical Monocytes ($CD14^{dim}$CD16+) CCR2 mids	W0: AUD+ vs PLWH Ref.	4.4221	21.6880	**0.0036**	2.6843	23.4259	**0.0101**
	W4: AUD+ vs PLWH Ref.	6.7717	24.2618	**0.0008**	5.0113	26.0222	**0.0021**
	W0-W4: AUD+ only	-11.3983	6.4750	0.5838	-13.1973	8.2740	0.8465
Non-classical Monocytes ($CD14^{dim}$CD16+) CCR2 bright	W0: AUD+ vs PLWH Ref.	-19.9167	4.7950	0.2258	-22.4040	7.2823	0.4440
	W4: AUD+ vs PLWH Ref.	-21.1771	3.8554	0.1715	-23.6967	6.3750	0.3557
	W0-W4: AUD+ only	-11.6905	13.8905	0.8640	-14.2653	16.4653	0.9838
Non-classical Monocytes ($CD14^{dim}$CD16+) CD163+	W0: AUD+ vs PLWH Ref.	-0.3584	14.3646	0.0619	-1.8403	15.8466	0.1467
	W4: AUD+ vs PLWH Ref.	3.3803	18.2945	**0.0051**	1.8791	19.7957	**0.0139**
	W0-W4: AUD+ only	-11.4548	3.7862	0.3183	-12.9888	5.3202	0.5759
Non-classical Monocytes ($CD14^{dim}$CD16+) PD-1+	W0: AUD+ vs PLWH Ref.	-1.9875	1.2351	0.6423	-2.3119	1.5595	0.8870
	W4: AUD+ vs PLWH Ref.	-1.6805	1.5840	0.9530	-2.0091	1.9125	0.9981
	W0-W4: AUD+ only	-1.9959	1.3401	0.6956	-2.3317	1.6759	0.9185
Non-classical Monocytes ($CD14^{dim}$CD16+) PD-1L+	W0: AUD+ vs PLWH Ref.	2.0753	11.5390	**0.0055**	1.1227	12.4916	**0.0151**
	W4: AUD+ vs PLWH Ref.	-0.7708	8.8158	0.0985	-1.7357	9.7807	0.2218
	W0-W4: AUD+ only	-2.1137	7.6830	0.2601	-3.0997	8.6690	0.4954
Non-classical Monocytes ($CD14^{dim}$CD16+) TLR4+	W0: AUD+/HCV+ vs PLWH Ref.	4.0188	29.7786	**0.0110**	-1.2170	35.0144	0.0787
	W0: AUD+/HCV- vs PLWH Ref.	-0.2414	20.0531	0.0555	-4.3664	24.1781	0.3013
	W4: AUD+/HCV+ vs PLWH Ref.	-1.8098	23.9500	0.0907	-7.0456	29.1859	0.4297

	W4: AUD+/HCV- vs PLWH Ref.	-0.2327	20.5334	0.0552	-4.4535	24.7542	0.2999
	W0-W4: AUD+/HCV+	-10.0364	21.6936	0.4651	-16.4857	28.1429	0.9473
	W0-W4: AUD+/HCV-	-11.6764	11.1874	0.9660	-16.3237	15.8347	1.0000
Non-classical Monocytes ($CD14^{dim}CD16+$) HLA-DR Geo Mean	W0: AUD+/HCV+ vs PLWH Ref.	718.9200	7116.1500	**0.0173**	-581.3600	8416.4300	0.1163
	W0: AUD+/HCV- vs PLWH Ref.	-109.8900	4930.1000	0.0605	-1134.3000	5954.5100	0.3214
	W4: AUD+/HCV+ vs PLWH Ref.	2695.7700	9093.0100	**0.0005**	1395.5000	10393.0000	**0.0044**
	W4: AUD+/HCV- vs PLWH Ref.	339.9700	5497.0500	**0.0272**	-708.2400	6545.2600	0.1710
	W0-W4: AUD+/HCV+	-5916.8000	1963.0800	0.3194	-7518.4300	3564.7200	0.8523
	W0-W4: AUD+/HCV-	-3347.4300	2330.6200	0.7213	-4501.5300	3484.7200	0.9964
CD3+ CD4+	W0: AUD+ vs PLWH Ref.	-7.168	8.443	0.8709	-8.738	10.013	0.9854
	W4: AUD+ vs PLWH Ref.	-6.364	9.248	0.7133	-7.933	10.817	0.9278
	W0-W4: AUD+ only	-8.697	7.088	0.8393	-10.283	8.674	0.9774
CD3+ CD4+ CCR5+	W0: AUD+ vs PLWH Ref.	-7.682	2.984	0.3821	-8.755	4.056	0.6548
	W4: AUD+ vs PLWH Ref.	-7.110	3.557	0.5081	-8.182	4.629	0.7843
	W0-W4: AUD+ only	-5.965	4.819	0.8326	-7.049	5.903	0.9755
CD3+ CD4+ CD28+	W0: AUD+ vs PLWH Ref.	-7.237	5.715	0.8151	-8.540	7.017	0.9701
	W4: AUD+ vs PLWH Ref.	-7.578	5.374	0.7350	-8.880	6.676	0.9384
	W0-W4: AUD+ only	-6.207	6.889	0.9175	-7.523	8.205	0.9941
CD3+ CD4+ CD38+	W0: AUD+ vs PLWH Ref.	-7.275	9.580	0.7856	-8.970	11.274	0.9597
	W4: AUD+ vs PLWH Ref.	-4.294	12.562	0.3308	-5.988	14.256	0.5921

	W0-W4: AUD+ only	-11.503	5.539	0.4870	-13.216	7.252	0.7649
CD3+ CD4+ HLA-DR+	W0: AUD+ vs PLWH Ref.	-6.653	1.668	0.2358	-7.489	2.505	0.4595
	W4: AUD+ vs PLWH Ref.	-6.129	2.192	0.3481	-6.965	3.028	0.6139
	W0-W4: AUD+ only	-4.730	3.683	0.8044	-5.576	4.528	0.9665
CD3+ CD4+ CD38+ HLA-DR+	W0: AUD+ vs PLWH Ref.	-3.428	0.096	0.0634	-3.782	0.450	0.1502
	W4: AUD+ vs PLWH Ref.	-2.880	0.645	0.2098	-3.234	0.999	0.4190
	W0-W4: AUD+ only	-2.330	1.233	0.5406	-2.688	1.591	0.8124
CD3+ CD4+ Lag3+	W0: AUD+ vs PLWH Ref.	-0.156	0.084	0.5503	-0.180	0.108	0.8203
	W4: AUD+ vs PLWH Ref.	-0.071	0.169	0.4210	-0.095	0.193	0.6983
	W0-W4: AUD+ only	-0.206	0.037	0.1678	-0.230	0.061	0.3495
CD3+ CD4+ PD-1+	W0: AUD+ vs PLWH Ref.	-10.771	3.481	0.3107	-12.204	4.914	0.5660
	W4: AUD+ vs PLWH Ref.	-11.412	2.841	0.2340	-12.845	4.273	0.4567
	W0-W4: AUD+ only	-6.564	7.846	0.8595	-8.013	9.294	0.9827
CD3+ CD4+ TEMRA (CCR7- CD45RA+)	W0: AUD+ vs PLWH Ref.	-0.844	1.229	0.7117	-1.053	1.438	0.9269
	W4: AUD+ vs PLWH Ref.	-0.721	1.353	0.5449	-0.929	1.561	0.8159
	W0-W4: AUD+ only	-1.171	0.925	0.8151	-1.382	1.136	0.9701
CD3+ CD4+ Effector Memory (CCR7- CD45RA-)	W0: AUD+ vs PLWH Ref.	-11.288	5.153	0.4588	-12.940	6.806	0.7375
	W4: AUD+ vs PLWH Ref.	-12.856	3.585	0.2642	-14.509	5.238	0.5014
	W0-W4: AUD+ only	-6.743	9.880	0.7075	-8.414	11.550	0.9248
CD3+ CD4+ Naive (CCR7+ CD45RA+)	W0: AUD+ vs PLWH Ref.	-1.167	10.474	0.1151	-2.337	11.644	0.2542
	W4: AUD+ vs PLWH Ref.	0.206	11.847	**0.0426**	-0.964	13.018	0.1045
	W0-W4: AUD+ only	-7.258	4.512	0.6427	-8.441	5.695	0.8873
CD3+ CD4+ Central Memory (CCR7+ CD45RA-)	W0: AUD+ vs PLWH Ref.	-8.092	4.555	0.5783	-9.363	5.826	0.8424
	W4: AUD+ vs PLWH Ref.	-8.010	4.637	0.5960	-9.281	5.908	0.8555
	W0-W4: AUD+ only	-6.475	6.311	0.9797	-7.760	7.597	0.9996

CD3+ CD4+ Central Memory (CD45RA- CCR7+) CD27+ Geo Mean	W0: AUD+ vs PLWH Ref.	413.250	2105.150	**0.0041**	243.180	2275.220	**0.0114**
	W4: AUD+ vs PLWH Ref.	301.250	1993.150	**0.0086**	131.180	2163.220	**0.0232**
	W0-W4: AUD+ only	-743.300	967.300	0.7945	-915.250	1139.250	0.9630
CD3+ CD4+ Naive (CD45RA+ CCR7+) CD27+ Geo Mean	W0: AUD+ vs PLWH Ref.	320.530	2101.680	**0.0085**	141.490	2280.720	**0.0227**
	W4: AUD+ vs PLWH Ref.	252.900	2034.040	**0.0127**	73.857	2213.080	**0.0335**
	W0-W4: AUD+ only	-832.780	968.050	0.8812	-1013.800	1149.070	0.9877
CD3+ CD4+ TEMRA (CD45RA+ CCR7-) CD27+ Geo Mean	W0: AUD+ vs PLWH Ref.	-622.340	1201.340	0.5282	-805.660	1384.660	0.8018
	W4: AUD+ vs PLWH Ref.	-978.700	844.980	0.8840	-1162.020	1028.300	0.9882
	W0-W4: AUD+ only	-565.550	1278.280	0.4428	-750.900	1463.620	0.7213
CD3+ CD4+ Effector Memory (CD45RA- CCR7-) CD27+ Geo Mean	W0: AUD+ vs PLWH Ref.	-314.770	803.610	0.3858	-427.180	916.030	0.6590
	W4: AUD+ vs PLWH Ref.	-390.670	727.700	0.5493	-503.090	840.120	0.8195
	W0-W4: AUD+ only	-489.460	641.270	0.7894	-603.120	754.940	0.9611
CD3+ CD4+ CD28- CD57+	W0: AUD+ vs PLWH Ref.	-5.600	7.096	0.8147	-6.876	8.372	0.9699
	W4: AUD+ vs PLWH Ref.	-5.292	7.404	0.7408	-6.568	8.680	0.9410
	W0-W4: AUD+ only	-6.726	6.110	0.9239	-8.017	7.400	0.9949
CD3+ CD8+	W0: AUD+ vs PLWH Ref.	-8.066	6.516	0.8325	-9.532	7.982	0.9755
	W4: AUD+ vs PLWH Ref.	-8.662	5.921	0.7085	-10.127	7.386	0.9253
	W0-W4: AUD+ only	-6.776	7.967	0.8723	-8.258	9.449	0.9857
CD3+ CD8+ CD28+	W0: AUD+ vs PLWH Ref.	-4.573	18.167	0.2368	-6.859	20.452	0.4610
	W4: AUD+ vs PLWH Ref.	-6.651	16.089	0.4101	-8.936	18.375	0.6864
	W0-W4: AUD+ only	-9.418	13.573	0.7193	-11.729	15.884	0.9308
CD3+ CD8+ CD38+	W0: AUD+ vs PLWH Ref.	-7.975	9.068	0.8984	-9.688	10.782	0.9910
	W4: AUD+ vs PLWH Ref.	-7.672	9.371	0.8428	-9.385	11.084	0.9784
	W0-W4: AUD+ only	-8.919	8.313	0.9443	-10.651	10.045	0.9973

CD3+ CD8+ HLA-DR+	W0: AUD+/HCV+ vs PLWH Ref.	-3.639	7.656	0.4797	-5.930	9.947	0.9532
	W0: AUD+/HCV- vs PLWH Ref.	-6.833	1.882	0.2604	-8.601	3.650	0.7869
	W4: AUD+/HCV+ vs PLWH Ref.	-0.447	10.847	0.0704	-2.739	13.139	0.3600
	W4: AUD+/HCV- vs PLWH Ref.	-6.854	1.861	0.2564	-8.622	3.629	0.7816
	W0-W4: AUD+/HCV+	-10.147	3.765	0.3625	-12.970	6.587	0.8891
	W0-W4: AUD+/HCV-	-4.731	4.773	0.9929	-6.659	6.701	1.0000
CD3+ CD8+ CD38+ HLA-DR +	W0: AUD+ vs PLWH Ref.	-4.334	0.225	0.0765	-4.792	0.684	0.1776
	W4: AUD+ vs PLWH Ref.	-4.261	0.298	0.0872	-4.720	0.756	0.1995
	W0-W4: AUD+ only	-2.377	2.232	0.9502	-2.840	2.696	0.9978
CD3+ CD8+ Lag3+	W0: AUD+ vs PLWH Ref.	-0.456	0.049	0.1119	-0.507	0.099	0.2481
	W4: AUD+ vs PLWH Ref.	-0.320	0.184	0.5915	-0.371	0.235	0.8522
	W0-W4: AUD+ only	-0.391	0.120	0.2928	-0.442	0.171	0.5418
CD3+ CD8+ PD-1+	W0: AUD+/HCV+ vs PLWH Ref.	-12.342	8.868	0.7444	-16.645	13.171	0.9974
	W0: AUD+/HCV- vs PLWH Ref.	-15.952	0.413	0.0623	-19.273	3.734	0.3291
	W4: AUD+/HCV+ vs PLWH Ref.	-5.485	15.725	0.3381	-9.788	20.028	0.8695
	W4: AUD+/HCV- vs PLWH Ref.	-16.532	-0.167	**0.0457**	-19.853	3.154	0.2596
	W0-W4: AUD+/HCV+	-19.920	6.205	0.2980	-25.220	11.506	0.8311

	W0-W4: AUD+/HCV-	-8.343	9.503	0.8970	-11.964	13.124	0.9999
CD3+ CD8+ TEMRA (CCR7- CD45RA+)	W0: AUD+ vs PLWH Ref.	-9.260	6.366	0.7126	-10.831	7.937	0.9274
	W4: AUD+ vs PLWH Ref.	-8.198	7.427	0.9218	-9.769	8.998	0.9947
	W0-W4: AUD+ only	-8.961	6.838	0.7892	-10.549	8.426	0.9611
CD3+ CD8+ Effector Memory (CCR7- CD45RA-)	W0: AUD+/HCV+ vs PLWH Ref.	-7.729	13.923	0.5694	-12.122	18.316	0.9787
	W0: AUD+/HCV- vs PLWH Ref.	-15.179	1.528	0.1075	-18.568	4.918	0.4821
	W4: AUD+/HCV+ vs PLWH Ref.	-7.386	14.265	0.5276	-11.779	18.658	0.9687
	W4: AUD+/HCV- vs PLWH Ref.	-17.392	-0.685	**0.0344**	-20.782	2.705	0.2075
	W0-W4: AUD+/HCV+	-13.678	12.992	0.9592	-19.089	18.403	1.0000
	W0-W4: AUD+/HCV-	-6.896	11.323	0.6288	-10.593	15.019	0.9884
CD3+ CD8+ Naïve (CCR7+ CD45RA+)	W0: AUD+ vs PLWH Ref.	-1.850	12.847	0.1399	-3.327	14.325	0.3002
	W4: AUD+ vs PLWH Ref.	-1.294	13.403	0.1047	-2.772	14.880	0.2341
	W0-W4: AUD+ only	-7.985	6.874	0.8817	-9.479	8.368	0.9878
CD3+ CD8+ Central memory (CCR7+ CD45RA-)	W0: AUD+ vs PLWH Ref.	-1.726	2.632	0.6794	-2.164	3.070	0.9095
	W4: AUD+ vs PLWH Ref.	-1.941	2.417	0.8281	-2.379	2.855	0.9742
	W0-W4: AUD+ only	-1.988	2.418	0.8460	-2.431	2.861	0.9793
CD3+ CD8+ Central memory (CD45RA- CCR7+) CD27+ Geo Mean	W0: AUD+ vs PLWH Ref.	180.090	1659.090	**0.0156**	31.418	1807.760	**0.0408**
	W4: AUD+ vs PLWH Ref.	8.906	1487.910	**0.0474**	-139.760	1636.580	0.1152
	W0-W4: AUD+ only	-576.490	918.860	0.6489	-726.810	1069.170	0.8913
CD3+ CD8+ Naive (CD45RA+ CCR7+) CD27+ Geo Mean	W0: AUD+ vs PLWH Ref.	863.370	2652.090	**0.0002**	683.570	2831.890	**0.0006**
	W4: AUD+ vs PLWH Ref.	916.960	2705.680	**0.0001**	737.160	2885.480	**0.0004**

	W0-W4: AUD+ only	-957.830	850.650	0.9061	-1139.620	1032.440	0.9923
CD3+ CD8+ TEMRA (CD45RA+ CCR7-) CD27+ Geo Mean	W0: AUD+ vs PLWH Ref.	-27.069	926.540	0.0641	-122.930	1022.400	0.1515
	W4: AUD+ vs PLWH Ref.	-137.570	816.040	0.1601	-233.430	911.900	0.3361
	W0-W4: AUD+ only	-371.570	592.570	0.6486	-468.490	689.490	0.8911
CD3+ CD8+ Effector memory (CD45RA- CCR7-) CD27+ Geo Mean	W0: AUD+ vs PLWH Ref.	-222.500	545.050	0.4043	-299.650	622.210	0.6800
	W4: AUD+ vs PLWH Ref.	-312.500	455.050	0.7118	-389.650	532.210	0.9270
	W0-W4: AUD+ only	-298.020	478.020	0.6447	-376.020	556.020	0.8886
CD3+ CD8+ CD28- CD57+	W0: AUD+ vs PLWH Ref.	-12.701	8.829	0.7206	-14.865	10.993	0.9314
	W4: AUD+ vs PLWH Ref.	-11.525	10.005	0.8883	-13.690	12.169	0.9891
	W0-W4: AUD+ only	-12.060	9.708	0.8298	-14.248	11.896	0.9747
**Soluble Mediators**				**Comparisons among cohorts**			
		**Lower 95% CI (unadjusted)**	**Upper 95% CI (unadjusted)**	**p-value (unadjusted)**	**Lower 95% CI (adjusted)**	**Upper 95% CI (adjusted)**	**p-value (adjusted)**
Log2_I_FABP	W0: AUD+/HCV+ vs PLWH Ref.	0.1013	1.1237	**0.0192**	-0.1020	1.3271	0.1301
	W0: AUD+/HCV- vs PLWH Ref.	-0.0693	0.7655	0.1014	-0.2354	0.9315	0.4691
	W4: AUD+/HCV+ vs PLWH Ref.	-0.1817	0.8407	0.2047	-0.3851	1.0440	0.7075
	W4: AUD+/HCV- vs PLWH Ref.	1.0440	0.3834	0.9053	-0.5949	0.5457	1.0000
	W0-W4: AUD+/HCV+	-0.3545	0.9206	0.3816	-0.6082	1.1743	0.9047
	W0-W4: AUD+/HCV-	-0.1012	0.8465	0.1222	-0.2897	1.0350	0.5289
Log2_LBP	W0: AUD+/HCV+ vs PLWH Ref.	0.1882	0.8988	**0.0030**	0.0470	1.0401	**0.0243**

	W0: AUD+/HCV- vs PLWH Ref.	0.0936	0.6595	**0.0095**	-0.0189	0.7720	0.0702
	W4: AUD+/HCV+ vs PLWH Ref.	0.1086	0.8192	**0.0109**	-0.0327	0.9605	0.0794
	W4: AUD+/HCV- vs PLWH Ref.	0.1580	0.7239	**0.0025**	0.0455	0.8364	**0.0206**
	W0-W4: AUD+/HCV+	-0.3652	0.5245	0.7239	-0.5421	0.7014	0.9966
	W0-W4: AUD+/HCV-	-0.3893	0.2605	0.6957	-0.5185	0.3896	0.9950
Log2_sCD14	W0: AUD+ vs PLWH Ref.	-0.0176	0.3815	0.0737	-0.0572	0.4210	0.1726
	W4: AUD+ vs PLWH Ref.	0.0143	0.4134	**0.0359**	-0.0253	0.4529	0.0898
	W0-W4: AUD+ only	-0.2426	0.1789	0.7654	-0.2844	0.2206	0.9519
Log2_sCD163	W0: AUD+/HCV+ vs PLWH Ref.	0.4855	1.2151	**0.0000**	0.3404	1.3601	**0.0001**
	W0: AUD+/HCV- vs PLWH Ref.	0.1306	0.7117	**0.0048**	0.0151	0.8272	**0.0379**
	W4: AUD+/HCV+ vs PLWH Ref.	0.7750	1.5046	**0.0000**	0.6300	1.6497	**0.0000**
	W4: AUD+/HCV- vs PLWH Ref.	0.2066	0.7877	**0.0009**	0.0911	0.9032	**0.0081**
	W0-W4: AUD+/HCV+	-0.7463	0.1672	0.2122	-0.9279	0.3488	0.7202
	W0-W4: AUD+/HCV-	-0.4095	0.2576	0.6533	-0.5422	0.3902	0.9914
sCD40L	W0: AUD+ vs PLWH Ref.	1.0832	3.5360	**0.0003**	0.8403	3.7789	**0.0008**
	W4: AUD+ vs PLWH Ref.	1.2419	3.7098	**0.0001**	0.9974	3.9542	**0.0003**
	W0-W4: AUD+ only	-1.4686	1.1361	0.8012	-1.7266	1.3941	0.9655

Log2_HEK Blue	W0: AUD+/HCV+ vs PLWH Ref.	0.7956	1.7511	**<.0001**	0.6052	1.9415	**<.0001**
	W0: AUD+/HCV- vs PLWH Ref.	0.3272	1.1075	**0.0004**	0.1718	1.2629	**0.0036**
	W4: AUD+/HCV+ vs PLWH Ref.	0.2939	1.2495	**0.0018**	0.1035	1.4398	**0.0149**
	W4: AUD+/HCV- vs PLWH Ref.	0.1121	0.8923	**0.0120**	-0.0434	1.0477	0.0868
	W0-W4: AUD+/HCV+	-0.0694	1.0727	0.0846	-0.2969	1.3003	0.4144
	W0-W4: AUD+/HCV-	-0.2019	0.6322	0.3092	-0.3681	0.7984	0.8453
**Plasma Cytokines**				**Comparisons among cohorts**			
		**Lower 95% CI (unadjusted)**	**Upper 95% CI (unadjusted)**	**p-value (unadjusted)**	**Lower 95% CI (adjusted)**	**Upper 95% CI (adjusted)**	**p-value (adjusted[1])**
IFNg	W0: AUD+/HCV+ vs PLWH Ref.	N/A*	N/A*	0.8555	N/A*	N/A*	0.9998
	W0: AUD+/HCV- vs PLWH Ref.	N/A*	N/A*	0.0660	N/A*	N/A*	0.3514
	W4: AUD+/HCV+ vs PLWH Ref.	N/A*	N/A*	**0.0089**	N/A*	N/A*	0.0672
	W4: AUD+/HCV- vs PLWH Ref.	N/A*	N/A*	1.0000	N/A*	N/A*	1.0000
	W0-W4: AUD+/HCV+	N/A*	N/A*	**0.0176**	N/A*	N/A*	0.1223
	W0-W4: AUD+/HCV-	N/A*	N/A*	0.1607	N/A*	N/A*	0.6259

IL29	W0: AUD+/HCV+ vs PLWH Ref.	N/A*	N/A*	**0.0249**	N/A*	N/A*	0.1641
	W0: AUD+/HCV- vs PLWH Ref.	N/A*	N/A*	0.6874	N/A*	N/A*	0.9945
	W4: AUD+/HCV+ vs PLWH Ref.	N/A*	N/A*	**0.0366**	N/A*	N/A*	0.2244
	W4: AUD+/HCV- vs PLWH Ref.	N/A*	N/A*	0.6204	N/A*	N/A*	0.9878
	W0-W4: AUD+/HCV+	N/A*	N/A*	0.5653	N/A*	N/A*	0.9787
	W0-W4: AUD+/HCV-	N/A*	N/A*	0.8844	N/A*	N/A*	0.9999
IL8	W0: AUD+/HCV+ vs PLWH Ref.	N/A*	N/A*	**0.0093**	N/A*	N/A*	0.0705
	W0: AUD+/HCV- vs PLWH Ref.	N/A*	N/A*	**0.0259**	N/A*	N/A*	0.1696
	W4: AUD+/HCV+ vs PLWH Ref.	N/A*	N/A*	0.1536	N/A*	N/A*	0.6101
	W4: AUD+/HCV- vs PLWH Ref.	N/A*	N/A*	**0.0203**	N/A*	N/A*	0.1384
	W0-W4: AUD+/HCV+	N/A*	N/A*	0.1797	N/A*	N/A*	0.6651
	W0-W4: AUD+/HCV-	N/A*	N/A*	0.9835	N/A*	N/A*	1.0000
IP10	W0: AUD+/HCV+ vs PLWH Ref.	N/A*	N/A*	0.1029	N/A*	N/A*	0.4775
	W0: AUD+/HCV- vs PLWH Ref.	N/A*	N/A*	0.1219	N/A*	N/A*	0.5319

	W4: AUD+/HCV+ vs PLWH Ref.	N/A*	N/A*	**0.0125**	N/A*	N/A*	0.0912
	W4: AUD+/HCV- vs PLWH Ref.	N/A*	N/A*	0.3864	N/A*	N/A*	0.9093
	W0-W4: AUD+/HCV+	N/A*	N/A*	0.8480	N/A*	N/A*	0.9997
	W0-W4: AUD+/HCV-	N/A*	N/A*	0.0842	N/A*	N/A*	0.4172
Fraktaline	W0: AUD+ vs PLWH Ref.	N/A*	N/A*	**0.0546**	N/A*	N/A*	0.1324
	W4: AUD+ vs PLWH Ref.	N/A*	N/A*	**0.0041**	N/A*	N/A*	**0.0115**
	W0-W4: AUD+ only	N/A*	N/A*	0.8176	N/A*	N/A*	0.9711
GMCSF	W0: AUD+ vs PLWH Ref.	N/A*	N/A*	0.6361	N/A*	N/A*	0.8839
	W4: AUD+ vs PLWH Ref.	N/A*	N/A*	0.6388	N/A*	N/A*	0.8856
	W0-W4: AUD+ only	N/A*	N/A*	0.3564	N/A*	N/A*	0.6262
IFNb	W0: AUD+ vs PLWH Ref.	N/A*	N/A*	0.3452	N/A*	N/A*	0.6124
	W4: AUD+ vs PLWH Ref.	N/A*	N/A*	0.3127	N/A*	N/A*	0.5708
	W0-W4: AUD+ only	N/A*	N/A*	0.9181	N/A*	N/A*	0.9942
IFNa	W0: AUD+ vs PLWH Ref.	N/A*	N/A*	0.5548	N/A*	N/A*	0.8252
	W4: AUD+ vs PLWH Ref.	N/A*	N/A*	0.4302	N/A*	N/A*	0.7099
	W0-W4: AUD+ only	N/A*	N/A*	0.8844	N/A*	N/A*	0.9884
IL-10	W0: AUD+ vs PLWH Ref.	N/A*	N/A*	0.7721	N/A*	N/A*	0.9548
	W4: AUD+ vs PLWH Ref.	N/A*	N/A*	0.4538	N/A*	N/A*	0.7342
	W0-W4: AUD+ only	N/A*	N/A*	0.6467	N/A*	N/A*	0.8907
IL-12p70	W0: AUD+ vs PLWH Ref.	N/A*	N/A*	0.5838	N/A*	N/A*	0.8476
	W4: AUD+ vs PLWH Ref.	N/A*	N/A*	**0.0071**	N/A*	N/A*	**0.0194**
	W0-W4: AUD+ only	N/A*	N/A*	0.0714	N/A*	N/A*	0.1686
IL-15	W0: AUD+ vs PLWH Ref.	N/A*	N/A*	0.0950	N/A*	N/A*	0.2169

	W4: AUD+ vs PLWH Ref.	N/A*	N/A*	0.4684	N/A*	N/A*	0.7487
	W0-W4: AUD+ only	N/A*	N/A*	0.1352	N/A*	N/A*	0.2938
IL-17a	W0: AUD+ vs PLWH Ref.	N/A*	N/A*	0.4321	N/A*	N/A*	0.7119
	W4: AUD+ vs PLWH Ref.	N/A*	N/A*	0.8480	N/A*	N/A*	0.9800
	W0-W4: AUD+ only	N/A*	N/A*	0.7122	N/A*	N/A*	0.9277
IL-18	W0: AUD+ vs PLWH Ref.	N/A*	N/A*	0.0734	N/A*	N/A*	0.1727
	W4: AUD+ vs PLWH Ref.	N/A*	N/A*	0.1767	N/A*	N/A*	0.3669
	W0-W4: AUD+ only	N/A*	N/A*	0.5604	N/A*	N/A*	0.8297
IL-1b	W0: AUD+ vs PLWH Ref.	N/A*	N/A*	**0.0090**	N/A*	N/A*	**0.0244**
	W4: AUD+ vs PLWH Ref.	N/A*	N/A*	0.1297	N/A*	N/A*	0.2837
	W0-W4: AUD+ only	N/A*	N/A*	0.1662	N/A*	N/A*	0.3490
IL-2	W0: AUD+ vs PLWH Ref.	N/A*	N/A*	0.1528	N/A*	N/A*	0.3255
	W4: AUD+ vs PLWH Ref.	N/A*	N/A*	0.0766	N/A*	N/A*	0.1795
	W0-W4: AUD+ only	N/A*	N/A*	0.6113	N/A*	N/A*	0.8674
IL-21	W0: AUD+ vs PLWH Ref.	N/A*	N/A*	0.7609	N/A*	N/A*	0.9502
	W4: AUD+ vs PLWH Ref.	N/A*	N/A*	0.9201	N/A*	N/A*	0.9945
	W0-W4: AUD+ only	N/A*	N/A*	0.9727	N/A*	N/A*	0.9994
IL-22	W0: AUD+ vs PLWH Ref.	N/A*	N/A*	0.6841	N/A*	N/A*	0.9128
	W4: AUD+ vs PLWH Ref.	N/A*	N/A*	0.7666	N/A*	N/A*	0.9526
	W0-W4: AUD+ only	N/A*	N/A*	0.6839	N/A*	N/A*	0.9127
IL-23	W0: AUD+ vs PLWH Ref.	N/A*	N/A*	0.5209	N/A*	N/A*	0.7969
	W4: AUD+ vs PLWH Ref.	N/A*	N/A*	0.3198	N/A*	N/A*	0.5800
	W0-W4: AUD+ only	N/A*	N/A*	0.1022	N/A*	N/A*	0.2311
IL-27	W0: AUD+ vs PLWH Ref.	N/A*	N/A*	0.8090	N/A*	N/A*	0.9683
	W4: AUD+ vs PLWH Ref.	N/A*	N/A*	0.6367	N/A*	N/A*	0.8843

	W0-W4: AUD+ only	N/A*	N/A*	0.4485	N/A*	N/A*	0.7288
IL-33	W0: AUD+ vs PLWH Ref.	N/A*	N/A*	0.1986	N/A*	N/A*	0.4033
	W4: AUD+ vs PLWH Ref.	N/A*	N/A*	0.2345	N/A*	N/A*	0.4599
	W0-W4: AUD+ only	N/A*	N/A*	0.7195	N/A*	N/A*	0.9314
IL-4	W0: AUD+ vs PLWH Ref.	N/A*	N/A*	**0.0426**	N/A*	N/A*	0.1056
	W4: AUD+ vs PLWH Ref.	N/A*	N/A*	0.6984	N/A*	N/A*	0.9206
	W0-W4: AUD+ only	N/A*	N/A*	0.1588	N/A*	N/A*	0.3362
IL-6	W0: AUD+ vs PLWH Ref.	N/A*	N/A*	0.1597	N/A*	N/A*	0.3377
	W4: AUD+ vs PLWH Ref.	N/A*	N/A*	0.1802	N/A*	N/A*	0.3729
	W0-W4: AUD+ only	N/A*	N/A*	0.9474	N/A*	N/A*	0.9976
IL-7	W0: AUD+ vs PLWH Ref.	N/A*	N/A*	0.1471	N/A*	N/A*	0.3153
	W4: AUD+ vs PLWH Ref.	N/A*	N/A*	0.7836	N/A*	N/A*	0.9593
	W0-W4: AUD+ only	N/A*	N/A*	0.1112	N/A*	N/A*	0.2487
IL-9	W0: AUD+ vs PLWH Ref.	N/A*	N/A*	**0.0362**	N/A*	N/A*	0.0909
	W4: AUD+ vs PLWH Ref.	N/A*	N/A*	0.1312	N/A*	N/A*	0.2865
	W0-W4: AUD+ only	N/A*	N/A*	0.6344	N/A*	N/A*	0.8828
ITAC	W0: AUD+ vs PLWH Ref.	N/A*	N/A*	0.0886	N/A*	N/A*	0.2042
	W4: AUD+ vs PLWH Ref.	N/A*	N/A*	0.2488	N/A*	N/A*	0.4812
	W0-W4: AUD+ only	N/A*	N/A*	0.7668	N/A*	N/A*	0.9527
MCP-3	W0: AUD+ vs PLWH Ref.	N/A*	N/A*	**0.0062**	N/A*	N/A*	**0.0171**
	W4: AUD+ vs PLWH Ref.	N/A*	N/A*	**0.0421**	N/A*	N/A*	0.1044
	W0-W4: AUD+ only	N/A*	N/A*	0.4606	N/A*	N/A*	0.7409
MIP-1a	W0: AUD+ vs PLWH Ref.	N/A*	N/A*	0.2227	N/A*	N/A*	0.4417
	W4: AUD+ vs PLWH Ref.	N/A*	N/A*	0.5752	N/A*	N/A*	0.8411
	W0-W4: AUD+ only	N/A*	N/A*	0.4226	N/A*	N/A*	0.7018

MIP-3a	W0: AUD+ vs PLWH Ref.	N/A*	N/A*	0.3228	N/A*	N/A*	0.5840
	W4: AUD+ vs PLWH Ref.	N/A*	N/A*	**0.0108**	N/A*	N/A*	**0.0291**
	W0-W4: AUD+ only	N/A*	N/A*	0.1597	N/A*	N/A*	0.3377
SDF-1a	W0: AUD+ vs PLWH Ref.	N/A*	N/A*	0.2312	N/A*	N/A*	0.4547
	W4: AUD+ vs PLWH Ref.	N/A*	N/A*	0.0634	N/A*	N/A*	0.1516
	W0-W4: AUD+ only	N/A*	N/A*	0.9387	N/A*	N/A*	0.9967
TGF-b1	W0: AUD+ vs PLWH Ref.	N/A*	N/A*	0.2264	N/A*	N/A*	0.4474
	W4: AUD+ vs PLWH Ref.	N/A*	N/A*	**0.0130**	N/A*	N/A*	**0.0348**
	W0-W4: AUD+ only	N/A*	N/A*	0.2024	N/A*	N/A*	0.4096
TGF-b2	W0: AUD+ vs PLWH Ref.	N/A*	N/A*	**0.0052**	N/A*	N/A*	**0.0144**
	W4: AUD+ vs PLWH Ref.	N/A*	N/A*	**0.0006**	N/A*	N/A*	**0.0016**
	W0-W4: AUD+ only	N/A*	N/A*	1.0000	N/A*	N/A*	1.0000
TGF-b3	W0: AUD+ vs PLWH Ref.	N/A*	N/A*	0.0726	N/A*	N/A*	0.1711
	W4: AUD+ vs PLWH Ref.	N/A*	N/A*	0.9098	N/A*	N/A*	0.9929
	W0-W4: AUD+ only	N/A*	N/A*	0.1105	N/A*	N/A*	0.2474
TNFa	W0: AUD+ vs PLWH Ref.	N/A*	N/A*	0.2720	N/A*	N/A*	0.5150
	W4: AUD+ vs PLWH Ref.	N/A*	N/A*	0.7333	N/A*	N/A*	0.9380
	W0-W4: AUD+ only	N/A*	N/A*	0.1627	N/A*	N/A*	0.3430

* confidence intervals not reported for nonparametric analyses

Supplemental Table 2. Descriptive statistics for plasma markers of intestinal damage, microbial burden, and systemic indicators of immune activation among CTN-055 CHOICES participants with and without hepatitis C virus (HCV), and PLWH Ref. group

Variable	Group	Seq*	N	Mean	Std Dev	Lower 95%	Upper 95%	Median	Lower Quartile	Upper Quartile
Log2_HEK_Blue	AUD+ HCV-W0	1	15	9.61	0.81	9.16	10.06	9.47	9.19	10.08
		2	15	9.61	0.81	9.17	10.06	9.38	9.11	10.14
	AUD+ HCV+W0	1	8	10.16	1.15	9.20	11.12	9.86	9.61	10.30
		2	8	10.18	1.22	9.16	11.20	9.88	9.65	10.33
	AUD+HCV-W4	1	15	9.37	0.61	9.03	9.70	9.37	8.84	9.91
		2	15	9.43	0.60	9.09	9.76	9.42	8.98	9.97
	AUD+HCV+ W4	1	8	9.66	0.79	9.00	10.32	9.49	9.18	9.86
		2	8	9.67	0.82	8.99	10.35	9.47	9.17	9.87
	PLWH Ref	1	20	8.82	0.87	8.42	9.23	8.75	8.41	9.25
		2	20	8.97	0.82	8.58	9.35	8.92	8.47	9.31
Log2_I_FABP	AUD+ HCV-W0	1	14	10.48	0.83	10.00	10.96	10.70	9.90	11.02
		2	14	10.53	0.82	10.05	11.00	10.70	9.98	11.08
	AUD+ HCV+W0	1	8	10.75	1.03	9.89	11.61	10.59	9.97	11.66
		2	8	10.79	0.97	9.97	11.60	10.62	10.06	11.66
	AUD+HCV-W4	1	15	10.11	0.84	9.65	10.58	9.82	9.73	10.88
		2	15	10.15	0.81	9.71	10.60	10.05	9.65	10.88
	AUD+HCV+ W4	1	8	10.48	0.90	9.73	11.24	10.63	9.72	10.98
		2	8	10.49	0.91	9.72	11.25	10.74	9.75	10.87
	PLWH Ref	1	27	10.16	1.01	9.76	10.56	10.23	9.47	10.92
		2	27	10.20	1.03	9.80	10.61	10.29	9.40	11.09
Log2_LBP	AUD+ HCV-W0	1	15	12.15	0.58	11.82	12.47	12.12	11.68	12.51
		2	15	12.18	0.58	11.86	12.50	12.16	11.75	12.59
	AUD+ HCV+W0	1	8	12.30	0.72	11.70	12.90	12.26	11.73	12.92
		2	8	12.36	0.78	11.71	13.01	12.39	11.72	12.95
	AUD+HCV-W4	1	15	12.24	0.43	12.00	12.48	12.15	12.02	12.63
		2	15	12.21	0.48	11.95	12.48	12.14	11.87	12.66
	AUD+HCV+ W4	1	8	12.32	0.82	11.63	13.01	12.40	11.51	12.97
		2	8	12.18	0.71	11.58	12.77	12.31	11.51	12.82

	PLWH Ref	1	28	11.85	0.74	11.56	12.14	11.78	11.45	12.22
		2	28	11.79	0.62	11.55	12.03	11.74	11.54	12.06
Log2_sCD14	AUD+ HCV-W0	1	15	20.49	0.59	20.17	20.82	20.60	19.98	20.98
		2	15	20.35	0.47	20.09	20.61	20.46	19.89	20.78
	AUD+ HCV+W0	1	8	20.39	0.48	19.99	20.80	20.36	19.97	20.86
		2	8	20.39	0.50	19.97	20.81	20.60	19.85	20.79
	AUD+HCV-W4	1	15	20.49	0.60	20.16	20.82	20.72	19.95	20.92
		2	15	20.46	0.55	20.16	20.77	20.59	20.11	20.86
	AUD+HCV+ W4	1	8	20.43	0.60	19.93	20.94	20.69	20.04	20.85
		2	8	20.34	0.60	19.84	20.83	20.30	19.86	20.78
	PLWH Ref	1	28	20.27	0.45	20.09	20.44	20.24	19.94	20.59
		2	28	20.21	0.51	20.01	20.40	20.25	19.89	20.54
Log2_sCD163	AUD+ HCV-W0	1	15	15.77	0.60	15.44	16.10	15.82	15.17	16.36
		2	15	15.67	0.62	15.32	16.01	15.70	15.18	16.20
	AUD+ HCV+W0	1	8	16.19	0.80	15.52	16.86	16.31	15.76	16.90
		2	8	16.10	0.81	15.43	16.78	16.23	15.60	16.66
	AUD+HCV-W4	1	15	15.85	0.57	15.54	16.17	15.73	15.32	16.28
		2	15	15.74	0.57	15.42	16.05	15.68	15.09	16.06
	AUD+HCV+ W4	1	8	16.59	0.60	16.09	17.09	16.45	16.19	16.93
		2	8	16.28	0.70	15.69	16.87	16.09	15.85	16.75
	PLWH Ref	1	28	15.42	0.69	15.15	15.68	15.50	14.91	15.72
		2	28	15.23	0.68	14.96	15.49	15.32	14.73	15.59
sCD40L	AUD+ HCV-W0	1	15	7.23	4.10	4.96	9.50	6.83	4.25	9.28
		2	15	8.67	4.76	6.03	11.30	8.47	5.37	10.62
	AUD+ HCV+W0	1	8	6.53	2.48	4.46	8.60	5.75	4.91	8.30
		2	8	7.14	2.08	5.40	8.88	7.09	5.32	8.90
	AUD+HCV-W4	1	15	7.72	3.69	5.68	9.77	6.71	5.86	9.57
		2	15	8.46	3.77	6.37	10.55	7.81	6.12	11.80
	AUD+HCV+ W4	1	8	6.92	2.65	4.71	9.14	6.96	4.52	9.22
		2	7	7.10	2.27	5.00	9.20	7.02	5.18	9.22
	PLWH Ref	1	28	5.05	2.30	4.16	5.95	5.07	3.71	6.30

		2	28	5.38	2.38	4.46	6.31	5.50	3.67	6.70

*Indicates values of individual experimental duplicates

Supplemental Table 3. Descriptive statistics for monocyte phenotypic markers among CTN-055 CHOICES participants with and without hepatitis C virus (HCV), and PLWH Ref. group

Variable	Group	N	Mean	Std Dev	Lower 95%	Upper 95%	Median	Lower Quartile	Upper Quartile
Total CD14+ CCR2 mid	AUD+ HCV-W0	14	5.51	1.73	4.51	6.50	5.22	4.82	5.83
Total CD14+ CCR2 mid	AUD+ HCV+W0	7	6.67	1.69	5.11	8.24	7.14	5.12	7.50
Total CD14+ CCR2 mid	AUD+ HCV-W4	13	9.62	8.75	4.34	14.91	6.46	5.70	9.16
Total CD14+ CCR2 mid	AUD+ HCV+W4	7	5.86	2.17	3.85	7.88	4.45	4.20	8.51
Total CD14+ CCR2 mid	PLHV Ref	22	5.37	3.35	3.88	6.85	4.62	3.23	6.24
Intermediate monocytes (CD14brightCD16+)	AUD+ HCV-W0	14	18.77	11.15	12.34	25.21	14.70	10.80	25.70
Intermediate monocytes (CD14brightCD16+)	AUD+ HCV+W0	7	7.69	3.85	4.13	11.25	7.21	4.47	10.50
Intermediate monocytes (CD14brightCD16+)	AUD+ HCV-W4	13	23.95	16.93	13.72	34.18	20.90	11.90	32.80
Intermediate monocytes (CD14brightCD16+)	AUD+ HCV+W4	7	19.55	7.89	12.25	26.85	17.30	15.70	25.60
Intermediate monocytes (CD14brightCD16+)	PLHV Ref	22	7.88	10.32	3.30	12.45	4.08	2.63	7.11
Intermediate monocytes CCR2+	AUD+ HCV-W0	14	99.64	0.38	99.42	99.86	99.75	99.50	100.00
Intermediate monocytes CCR2+	AUD+ HCV+W0	7	99.54	0.85	98.75	100.33	100.00	99.30	100.00
Intermediate monocytes CCR2+	AUD+ HCV-W4	13	99.44	0.75	98.99	99.89	99.70	99.50	99.90
Intermediate monocytes CCR2+	AUD+ HCV+W4	7	99.89	0.12	99.77	100.00	99.90	99.80	100.00
Intermediate monocytes CCR2+	PLHV Ref	22	98.84	1.87	98.01	99.67	99.65	98.90	100.00
Intermediate monocytes CCR2mid	AUD+ HCV-W0	14	6.05	3.59	3.97	8.12	4.73	3.56	8.29
Intermediate monocytes CCR2mid	AUD+ HCV+W0	7	9.15	7.38	2.33	15.98	7.92	1.67	15.80
Intermediate monocytes CCR2mid	AUD+ HCV-W4	13	12.79	16.00	3.12	22.45	8.35	4.79	11.30

Intermediate monocytes CCR2mid	AUD+ HCV+W4	7	5.58	4.12	1.77	9.39	4.05	1.77	8.84
Intermediate monocytes CCR2mid	PLHV Ref	22	9.28	7.15	6.11	12.45	8.51	3.90	16.80
Intermediate monocytes CCR2 bright	AUD+ HCV-W0	14	93.60	3.88	91.36	95.84	95.07	91.51	96.19
Intermediate monocytes CCR2 bright	AUD+ HCV+W0	7	90.39	8.14	82.87	97.91	92.08	83.50	98.33
Intermediate monocytes CCR2 bright	AUD+ HCV-W4	13	86.65	16.69	76.57	96.74	91.45	87.60	95.11
Intermediate monocytes CCR2 bright	AUD+ HCV+W4	7	94.31	4.24	90.39	98.22	95.85	90.96	98.23
Intermediate monocytes CCR2 bright	PLHV Ref	22	89.55	8.70	85.69	93.41	91.29	82.40	96.10
Intermediate monocytes CD163+	AUD+ HCV-W0	14	85.16	8.56	80.22	90.11	86.50	83.40	92.70
Intermediate monocytes CD163+	AUD+ HCV+W0	7	87.26	6.38	81.36	93.16	90.70	81.10	92.80
Intermediate monocytes CD163+	AUD+ HCV-W4	13	83.85	7.71	79.19	88.50	85.10	81.20	88.30
Intermediate monocytes CD163+	AUD+ HCV+W4	7	87.71	7.16	81.10	94.33	88.00	82.00	93.70
Intermediate monocytes CD163+	PLHV Ref	22	79.04	14.69	72.53	85.55	81.30	76.10	89.90
Intermediate monocytes PD-1+	AUD+ HCV-W0	14	3.41	1.91	2.30	4.51	2.76	1.93	4.53
Intermediate monocytes PD-1+	AUD+ HCV+W0	7	1.82	0.47	1.38	2.25	1.89	1.56	2.09
Intermediate monocytes PD-1+	AUD+ HCV-W4	13	2.46	1.87	1.33	3.59	1.53	1.35	3.10
Intermediate monocytes PD-1+	AUD+ HCV+W4	7	1.71	0.83	0.94	2.47	1.24	1.14	2.57
Intermediate monocytes PD-1+	PLHV Ref	22	1.95	1.47	1.30	2.60	2.07	0.66	2.66
Intermediate monocytes PD-1L+	AUD+ HCV-W0	14	15.75	7.95	11.16	20.34	13.35	8.60	21.60

Intermediate monocytes PD-1L+	AUD+ HCV+W0	7	9.86	6.28	4.05	15.66	8.11	6.33	11.40
Intermediate monocytes PD-1L+	AUD+ HCV-W4	13	13.50	6.36	9.65	17.34	13.20	7.98	18.70
Intermediate monocytes PD-1L+	AUD+ HCV+W4	7	7.74	3.72	4.30	11.18	6.68	4.47	12.40
Intermediate monocytes PD-1L+	PLHV Ref	22	15.26	8.96	11.28	19.23	14.45	7.01	22.50
Intermediate monocytes TLR4+	AUD+ HCV-W0	14	23.50	12.90	16.06	30.95	18.85	16.50	31.70
Intermediate monocytes TLR4+	AUD+ HCV+W0	7	33.56	12.31	22.18	44.94	30.40	27.40	42.30
Intermediate monocytes TLR4+	AUD+ HCV-W4	13	23.86	8.98	18.43	29.29	19.30	17.80	26.50
Intermediate monocytes TLR4+	AUD+ HCV+W4	7	26.49	11.71	15.65	37.32	22.40	19.70	32.30
Intermediate monocytes TLR4+	PLHV Ref	22	21.47	13.30	15.58	27.37	17.85	12.70	31.80
Intermediate monocytes CD163+ Geo Mean	AUD+ HCV-W0	14	2025.71	596.81	1681.13	2370.30	2231.00	1321.00	2513.00
Intermediate monocytes CD163+ Geo Mean	AUD+ HCV+W0	7	1967.43	874.31	1158.82	2776.03	1670.00	1186.00	2798.00
Intermediate monocytes CD163+ Geo Mean	AUD+ HCV-W4	13	1852.92	827.87	1352.64	2353.20	1814.00	1155.00	2159.00
Intermediate monocytes CD163+ Geo Mean	AUD+ HCV+W4	7	2229.14	1037.23	1269.87	3188.42	2345.00	1326.00	3230.00
Intermediate monocytes CD163+ Geo Mean	PLHV Ref	22	1511.09	609.23	1240.97	1781.21	1405.50	1123.00	1929.00
Intermediate monocytes HLA-DR+ Geo Mean	AUD+ HCV-W0	14	5653.93	2672.25	4111.02	7196.84	4805.00	3396.00	7459.00
Intermediate monocytes HLA-DR+ Geo Mean	AUD+ HCV+W0	7	7752.29	4055.27	4001.79	11502.78	5189.00	4241.00	12783.00
Intermediate monocytes HLA-DR+ Geo Mean	AUD+ HCV-W4	13	6381.92	3107.16	4504.28	8259.56	5745.00	4345.00	8063.00
Intermediate monocytes HLA-DR+ Geo Mean	AUD+ HCV+W4	7	6386.14	2063.62	4477.61	8294.68	6672.00	4233.00	7642.00

Intermediate monocytes HLA-DR+ Geo Mean	PLHV Ref	22	5452.27	1703.61	4696.93	6207.61	5085.50	4087.00	7008.00
Total CD14+ CCR2 bright	AUD+ HCV-W0	14	93.28	1.98	92.14	94.42	93.88	92.49	94.58
Total CD14+ CCR2 bright	AUD+ HCV+W0	7	92.18	2.33	90.02	94.34	92.10	91.19	93.77
Total CD14+ CCR2 bright	AUD+ HCV-W4	13	88.39	9.87	82.42	94.35	92.17	87.34	93.47
Total CD14+ CCR2 bright	AUD+ HCV+W4	7	93.36	2.59	90.97	95.76	94.90	90.39	95.34
Total CD14+ CCR2 bright	PLHV Ref	22	93.75	3.76	92.08	95.41	94.26	92.56	95.77
Classical Monocytes (CD14brightCD16-)	AUD+ HCV-W0	14	75.56	11.56	68.89	82.24	79.70	70.30	82.10
Classical Monocytes (CD14brightCD16-)	AUD+ HCV+W0	7	83.90	4.34	79.88	87.92	84.40	80.70	87.60
Classical Monocytes (CD14brightCD16-)	AUD+ HCV-W4	13	68.55	17.74	57.83	79.27	72.30	60.10	81.40
Classical Monocytes (CD14brightCD16-)	AUD+ HCV+W4	7	75.04	8.19	67.47	82.62	75.10	70.10	76.80
Classical Monocytes (CD14brightCD16-)	PLHV Ref	22	85.33	9.21	81.25	89.41	87.50	84.70	90.80
Classical Monocytes CCR2+	AUD+ HCV-W0	14	99.86	0.14	99.78	99.94	99.90	99.80	100.00
Classical Monocytes CCR2+	AUD+ HCV+W0	7	99.91	0.09	99.83	100.00	99.90	99.80	100.00
Classical Monocytes CCR2+	AUD+ HCV-W4	13	99.75	0.29	99.58	99.93	99.90	99.70	99.90
Classical Monocytes CCR2+	AUD+ HCV+W4	7	99.84	0.11	99.74	99.95	99.90	99.70	99.90
Classical Monocytes CCR2+	PLHV Ref	22	99.81	0.17	99.73	99.89	99.90	99.70	99.90
Classical Monocytes CCR2mids	AUD+ HCV-W0	14	3.59	2.27	2.28	4.89	2.98	1.77	4.77
Classical Monocytes CCR2mids	AUD+ HCV+W0	7	3.18	2.29	1.06	5.29	2.18	1.67	5.52
Classical Monocytes CCR2mids	AUD+ HCV-W4	13	4.58	2.95	2.79	6.36	3.86	2.09	6.27
Classical Monocytes CCR2mids	AUD+ HCV+W4	7	4.33	3.06	1.50	7.15	3.44	2.26	6.25

Classical Monocytes CCR2mids	PLHV Ref	22	4.01	3.16	2.61	5.41	3.17	2.02	4.85
Classical Monocytes CCR2 bright	AUD+ HCV-W0	14	96.27	2.33	94.93	97.62	96.93	95.03	98.16
Classical Monocytes CCR2 bright	AUD+ HCV+W0	7	96.74	2.36	94.55	98.93	97.82	94.28	98.33
Classical Monocytes CCR2 bright	AUD+ HCV-W4	13	95.18	3.19	93.25	97.10	96.04	93.63	97.81
Classical Monocytes CCR2 bright	AUD+ HCV+W4	7	95.51	3.14	92.61	98.42	96.46	93.45	97.57
Classical Monocytes CCR2 bright	PLHV Ref	22	95.80	3.30	94.34	97.26	96.68	94.75	97.79
Classical Monocytes CD163+	AUD+ HCV-W0	14	76.49	10.60	70.37	82.61	78.80	70.80	85.50
Classical Monocytes CD163+	AUD+ HCV+W0	7	73.37	10.07	64.06	82.69	77.30	66.90	78.60
Classical Monocytes CD163+	AUD+ HCV-W4	13	72.85	9.74	66.96	78.73	73.70	65.30	79.90
Classical Monocytes CD163+	AUD+ HCV+W4	7	73.77	13.79	61.02	86.52	75.70	61.40	85.60
Classical Monocytes CD163+	PLHV Ref	22	67.85	12.40	62.35	73.35	69.80	60.90	74.80
Classical Monocytes PD-1+	AUD+ HCV-W0	14	0.41	0.42	0.17	0.65	0.31	0.14	0.44
Classical Monocytes PD-1+	AUD+ HCV+W0	7	0.29	0.27	0.04	0.54	0.11	0.06	0.56
Classical Monocytes PD-1+	AUD+ HCV-W4	13	0.49	0.52	0.17	0.80	0.31	0.16	0.61
Classical Monocytes PD-1+	AUD+ HCV+W4	7	0.24	0.18	0.08	0.40	0.19	0.10	0.47
Classical Monocytes PD-1+	PLHV Ref	22	0.29	0.32	0.14	0.43	0.17	0.12	0.27
Classical Monocytes PD-1L+	AUD+ HCV-W0	14	3.64	1.75	2.63	4.65	4.11	2.30	5.36
Classical Monocytes PD-1L+	AUD+ HCV+W0	7	3.15	4.10	-0.65	6.94	1.34	1.19	2.95
Classical Monocytes PD-1L+	AUD+ HCV-W4	13	4.21	3.34	2.20	6.23	3.24	1.52	5.03

Classical Monocytes PD-1L+	AUD+ HCV+W4	7	1.90	1.37	0.64	3.16	1.36	0.64	2.96
Classical Monocytes PD-1L+	PLHV Ref	22	3.62	2.88	2.35	4.90	2.60	1.70	5.56
Classical Monocytes TLR4+	AUD+ HCV-W0	14	11.54	9.02	6.33	16.75	9.40	6.60	14.90
Classical Monocytes TLR4+	AUD+ HCV+W0	7	20.37	8.44	12.57	28.17	21.80	17.00	27.40
Classical Monocytes TLR4+	AUD+ HCV-W4	13	11.51	4.59	8.74	14.29	10.40	7.20	15.90
Classical Monocytes TLR4+	AUD+ HCV+W4	7	15.14	9.32	6.52	23.76	13.20	10.30	13.50
Classical Monocytes TLR4+	PLHV Ref	22	5.01	2.96	3.69	6.32	4.70	2.86	6.70
Classical Monocytes CD163+ Geo Mean	AUD+ HCV-W0	14	1586.43	597.19	1241.62	1931.23	1552.00	936.00	2084.00
Classical Monocytes CD163+ Geo Mean	AUD+ HCV+W0	7	1495.57	622.56	919.80	2071.35	1351.00	785.00	2010.00
Classical Monocytes CD163+ Geo Mean	AUD+ HCV-W4	13	1425.85	658.23	1028.08	1823.61	1230.00	932.00	1905.00
Classical Monocytes CD163+ Geo Mean	AUD+ HCV+W4	7	1583.14	785.46	856.71	2309.57	1647.00	833.00	2150.00
Classical Monocytes CD163+ Geo Mean	PLHV Ref	22	1106.68	438.16	912.41	1300.95	1032.00	832.00	1402.00
Classical Monocytes HLA-DR+ Geo Mean	AUD+ HCV-W0	14	4449.64	2583.87	2957.76	5941.52	3278.50	2326.00	5397.00
Classical Monocytes HLA-DR+ Geo Mean	AUD+ HCV+W0	7	5015.86	2704.25	2514.85	7516.87	4189.00	3334.00	6100.00
Classical Monocytes HLA-DR+ Geo Mean	AUD+ HCV-W4	13	4134.69	1421.41	3275.75	4993.64	4051.00	3023.00	5346.00
Classical Monocytes HLA-DR+ Geo Mean	AUD+ HCV+W4	7	4508.43	1549.70	3075.20	5941.66	4063.00	3366.00	4770.00
Classical Monocytes HLA-DR+ Geo Mean	PLHV Ref	22	3775.68	1169.87	3256.99	4294.37	3612.00	2884.00	4544.00
Total CD14+ CD163+	AUD+ HCV-W0	14	74.81	12.07	67.84	81.78	78.70	66.20	85.60
Total CD14+ CD163+	AUD+ HCV+W0	7	71.76	9.57	62.91	80.61	71.40	66.90	77.60
Total CD14+ CD163+	AUD+ HCV-W4	13	72.70	10.42	66.40	79.00	72.70	68.40	78.90
Total CD14+ CD163+	AUD+ HCV+W4	7	74.93	12.41	63.45	86.40	78.30	62.50	86.30
Total CD14+ CD163+	PLHV Ref	22	63.62	15.58	56.72	70.53	67.80	56.50	74.10

Non-Classical Monocytes ($CD14^{dim}CD16+$)	AUD+ HCV-W0	14	2.16	1.08	1.54	2.79	2.09	1.28	2.81
Non-Classical Monocytes ($CD14^{dim}CD16+$)	AUD+ HCV+W0	7	2.10	1.30	0.90	3.31	1.78	1.06	3.56
Non-Classical Monocytes ($CD14^{dim}CD16+$)	AUD+ HCV-W4	13	3.75	2.82	2.04	5.45	2.80	2.14	4.03
Non-Classical Monocytes ($CD14^{dim}CD16+$)	AUD+ HCV+W4	7	2.07	0.91	1.22	2.91	1.97	1.25	2.40
Non-Classical Monocytes ($CD14^{dim}CD16+$)	PLHV Ref	22	0.88	0.68	0.58	1.19	0.71	0.35	1.34
Non-Classical Monocytes CCR2+	AUD+ HCV-W0	14	84.01	11.37	77.44	90.57	88.85	80.80	91.80
Non-Classical Monocytes CCR2+	AUD+ HCV+W0	7	82.29	12.78	70.46	94.11	87.50	70.00	89.20
Non-Classical Monocytes CCR2+	AUD+ HCV-W4	13	81.32	11.19	74.55	88.08	83.20	73.80	91.20
Non-Classical Monocytes CCR2+	AUD+ HCV+W4	7	91.26	6.94	84.84	97.67	92.50	84.60	97.80
Non-Classical Monocytes CCR2+	PLHV Ref	22	78.35	16.33	71.12	85.59	79.55	67.90	93.00
Non-Classical Monocytes CCR2mids	AUD+ HCV-W0	14	56.14	7.96	51.55	60.74	56.00	51.00	60.30
Non-Classical Monocytes CCR2mids	AUD+ HCV+W0	7	51.01	17.85	34.51	67.52	47.50	42.00	60.00
Non-Classical Monocytes CCR2mids	AUD+ HCV-W4	13	59.94	9.25	54.35	65.53	58.30	56.70	66.10
Non-Classical Monocytes CCR2mids	AUD+ HCV+W4	7	51.24	20.19	32.57	69.91	50.70	31.20	71.20
Non-Classical Monocytes CCR2mids	PLHV Ref	22	41.09	16.57	33.74	48.44	43.20	27.90	48.40
Non-Classical Monocytes CCR2 brights	AUD+ HCV-W0	14	27.86	13.92	19.83	35.90	28.60	20.50	40.50
Non-Classical Monocytes CCR2 brights	AUD+ HCV+W0	7	31.27	20.28	12.51	50.03	30.40	13.80	45.70
Non-Classical Monocytes CCR2 brights	AUD+ HCV-W4	13	21.38	11.73	14.29	28.46	19.80	14.00	32.90
Non-Classical Monocytes CCR2 brights	AUD+ HCV+W4	7	40.01	21.62	20.02	60.01	34.40	20.90	61.30

Non-Classical Monocytes CCR2 brights	PLHV Ref	22	37.26	26.04	25.72	48.81	35.65	14.30	60.60
Non-Classical Monocytes CD163+	AUD+ HCV-W0	14	74.27	14.13	66.11	82.43	80.10	64.60	85.40
Non-Classical Monocytes CD163+	AUD+ HCV+W0	7	69.51	12.52	57.94	81.09	70.60	59.50	82.10
Non-Classical Monocytes CD163+	AUD+ HCV-W4	13	76.42	10.25	70.22	82.61	78.50	69.30	82.90
Non-Classical Monocytes CD163+	AUD+ HCV+W4	7	76.71	5.82	71.33	82.10	77.80	70.20	81.30
Non-Classical Monocytes CD163+	PLHV Ref	22	65.21	13.55	59.20	71.22	69.00	56.20	73.70
Non-Classical Monocytes PD-1+	AUD+ HCV-W0	14	2.15	1.38	1.35	2.94	2.44	1.27	3.30
Non-Classical Monocytes PD-1+	AUD+ HCV+W0	7	2.93	1.81	1.25	4.60	3.08	1.23	4.69
Non-Classical Monocytes PD-1+	AUD+ HCV-W4	13	2.18	2.26	0.82	3.54	1.35	0.41	3.45
Non-Classical Monocytes PD-1+	AUD+ HCV+W4	7	3.77	2.87	1.12	6.43	3.68	0.65	6.59
Non-Classical Monocytes PD-1+	PLHV Ref	22	2.87	3.53	1.30	4.44	2.27	0.00	3.23
Non-Classical Monocytes PD-1L+	AUD+ HCV-W0	14	17.06	7.51	12.72	21.39	16.95	11.50	19.80
Non-Classical Monocytes PD-1L+	AUD+ HCV+W0	7	16.37	10.70	6.47	26.27	12.20	9.88	24.10
Non-Classical Monocytes PD-1L+	AUD+ HCV-W4	13	14.93	7.57	10.36	19.50	14.30	11.30	15.70
Non-Classical Monocytes PD-1L+	AUD+ HCV+W4	7	12.39	5.93	6.91	17.88	11.80	7.46	13.70
Non-Classical Monocytes PD-1L+	PLHV Ref	22	9.73	8.03	6.16	13.29	7.57	3.54	14.30
Non-Classical Monocytes TLR4+	AUD+ HCV-W0	14	53.38	12.44	46.19	60.56	53.00	45.70	66.70
Non-Classical Monocytes TLR4+	AUD+ HCV+W0	7	60.37	14.57	46.90	73.84	65.40	46.90	73.00
Non-Classical Monocytes TLR4+	AUD+ HCV-W4	13	53.62	16.46	43.67	63.57	49.60	45.90	70.80

Non-Classical Monocytes TLR4+	AUD+ HCV+W4	7	54.54	9.46	45.79	63.29	54.30	47.80	60.20
Non-Classical Monocytes TLR4+	PLHV Ref	22	43.47	16.44	36.18	50.76	46.15	31.50	54.50
Non-Classical Monocytes CD163+ Geo Mean	AUD+ HCV-W0	14	1196.29	487.19	914.99	1477.58	1222.50	719.00	1672.00
Non-Classical Monocytes CD163+ Geo Mean	AUD+ HCV+W0	7	980.86	330.74	674.98	1286.74	986.00	790.00	1262.00
Non-Classical Monocytes CD163+ Geo Mean	AUD+ HCV-W4	13	1139.38	438.45	874.43	1404.34	1016.00	882.00	1452.00
Non-Classical Monocytes CD163+ Geo Mean	AUD+ HCV+W4	7	1350.00	335.58	1039.64	1660.36	1270.00	1099.00	1420.00
Non-Classical Monocytes CD163+ Geo Mean	PLHV Ref	22	929.64	418.24	744.20	1115.07	958.00	583.00	1089.00
Non-Classical Monocytes HLA-DR+ Geo Mean	AUD+ HCV-W0	14	8424.29	3380.68	6472.34	10376.23	7991.00	6291.00	11137.00
Non-Classical Monocytes HLA-DR+ Geo Mean	AUD+ HCV+W0	7	9931.71	5920.68	4456.00	15407.43	7648.00	6133.00	15409.00
Non-Classical Monocytes HLA-DR+ Geo Mean	AUD+ HCV-W4	13	8932.69	3509.19	6812.11	11053.27	8147.00	7162.00	9835.00
Non-Classical Monocytes HLA-DR+ Geo Mean	AUD+ HCV+W4	7	11908.57	5102.16	7189.86	16627.28	11663.00	7168.00	17339.00
Non-Classical Monocytes HLA-DR+ Geo Mean	PLHV Ref	22	6014.18	2425.83	4938.63	7089.74	5453.00	4271.00	6760.00
Total CD14+ PD-1+	AUD+ HCV-W0	14	0.46	0.38	0.24	0.68	0.36	0.22	0.77
Total CD14+ PD-1+	AUD+ HCV+W0	7	0.36	0.37	0.02	0.71	0.20	0.07	0.65
Total CD14+ PD-1+	AUD+ HCV-W4	13	0.42	0.39	0.19	0.66	0.28	0.14	0.56
Total CD14+ PD-1+	AUD+ HCV+W4	7	0.28	0.33	-0.03	0.58	0.14	0.06	0.48
Total CD14+ PD-1+	PLHV Ref	22	0.24	0.24	0.14	0.35	0.15	0.12	0.21
Total CD14+ PD-1L+	AUD+ HCV-W0	14	4.14	2.34	2.79	5.49	4.09	2.26	5.63
Total CD14+ PD-1L+	AUD+ HCV+W0	7	3.75	4.02	0.03	7.47	2.15	1.41	5.63
Total CD14+ PD-1L+	AUD+ HCV-W4	13	4.28	3.06	2.43	6.13	3.14	1.50	7.22
Total CD14+ PD-1L+	AUD+ HCV+W4	7	2.98	2.82	0.37	5.58	1.47	0.85	6.34
Total CD14+ PD-1L+	PLHV Ref	22	4.18	3.14	2.79	5.57	2.96	1.93	6.60
Total CD14+ TLR4+	AUD+ HCV-W0	14	10.11	7.13	6.00	14.23	8.28	6.57	13.00
Total CD14+ TLR4+	AUD+ HCV+W0	7	17.04	6.40	11.12	22.96	18.30	15.00	20.80

Total CD14+ TLR4+	AUD+ HCV-W4	13	9.81	3.60	7.64	11.98	9.88	6.46	11.70
Total CD14+ TLR4+	AUD+ HCV+W4	7	12.34	6.51	6.32	18.35	10.80	8.07	11.80
Total CD14+ TLR4+	PLHV Ref	22	3.96	2.57	2.82	5.10	3.05	2.21	5.75
Total CD14+| CD163+ Geo Mean	AUD+ HCV-W0	14	1643.50	572.81	1312.77	1974.23	1799.00	921.00	2023.00
Total CD14+| CD163+ Geo Mean	AUD+ HCV+W0	7	1509.14	652.86	905.35	2112.94	1265.00	827.00	2298.00
Total CD14+| CD163+ Geo Mean	AUD+ HCV-W4	13	1459.23	651.74	1065.39	1853.07	1240.00	961.00	1819.00
Total CD14+| CD163+ Geo Mean	AUD+ HCV+W4	7	1644.00	760.57	940.59	2347.41	1803.00	892.00	2215.00
Total CD14+| CD163+ Geo Mean	PLHV Ref	22	1090.73	420.46	904.31	1277.15	1103.50	804.00	1356.00
Total CD14+ HLA-DR+ Geo Mean	AUD+ HCV-W0	14	4691.00	2611.37	3183.24	6198.76	3534.00	2448.00	5671.00
Total CD14+ HLA-DR+ Geo Mean	AUD+ HCV+W0	7	5279.29	2918.86	2579.79	7978.78	4686.00	3388.00	6481.00
Total CD14+ HLA-DR+ Geo Mean	AUD+ HCV-W4	13	4504.62	1649.11	3508.07	5501.16	4174.00	3304.00	5882.00
Total CD14+ HLA-DR+ Geo Mean	AUD+ HCV+W4	7	4841.86	1705.77	3264.28	6419.43	4525.00	3664.00	5097.00
Total CD14+ HLA-DR+ Geo Mean	PLHV Ref	22	3777.55	1181.42	3253.73	4301.36	3744.00	2875.00	4240.00
Monocyte-Derived Suppressor Cells (CD14+CD11b+ HLA-DRmidCD15-)	AUD+ HCV-W0	14	0.88	0.91	0.36	1.41	0.55	0.38	1.01
Monocyte-Derived Suppressor Cells (CD14+CD11b+ HLA-DRmidCD15-)	AUD+ HCV+W0	7	2.09	2.27	-0.01	4.18	1.59	0.76	2.07
Monocyte-Derived Suppressor Cells (CD14+CD11b+ HLA-DRmidCD15-)	AUD+ HCV-W4	13	0.75	0.55	0.42	1.08	0.63	0.29	1.15
Monocyte-Derived Suppressor Cells (CD14+CD11b+ HLA-DRmidCD15-)	AUD+ HCV+W4	7	1.16	0.87	0.36	1.97	0.96	0.52	1.91
Monocyte-Derived Suppressor Cells (CD14+CD11b+ HLA-DRmidCD15-)	PLHV Ref	22	1.70	1.87	0.87	2.53	1.19	0.60	2.22

Supplemental Table 4. Spearman correlation analysis.

**Spearman Correlation Analysis**	**All cohorts**	
Comparison	Correlation (r_s)	p-value
TLR4 (%) vs I-FABP (log2 pg/mL)	0.13	0.3328
TLR4 (%) vs LBP (log2 ng/mL)	0.10	0.4454
TLR4 (%) vs HEK-Blue hTLR4 (log2 pg/mL)	0.20	0.1469
TLR4 (%) vs sCD14 (log2 pg/mL)	0.13	0.3075
TLR4 (%) vs sCD163 (log2 pg/mL)	0.37	**0.0028**
I-FABP (log2 pg/mL) vs LBP (log2 ng/mL)	0.07	0.5783
I-FABP (log2 pg/mL) vs HEK-Blue hTLR4 (log2 pg/mL)	0.12	0.3309
I-FABP (log2 pg/mL) vs sCD14 (log2 pg/mL)	0.36	**0.0019**
I-FABP (log2 pg/mL) vs sCD163 (log2 pg/mL)	0.12	0.3070
LBP (log2 ng/mL) vs HEK-Blue hTLR4 (log2 pg/mL)	0.43	**0.0003**
LBP (log2 ng/mL) vs sCD163 (log2 pg/mL)	0.17	0.1466
HEK-Blue hTLR4 (log2 pg/mL) vs sCD14 (log2 pg/mL)	0.16	0.2051
HEK-Blue hTLR4 (log2 pg/mL) vs sCD163 (log2 pg/mL)	0.18	0.1552
sCD14 (log2 pg/mL) vs sCD163 (log2 pg/mL)	0.08	0.5039

Supplemental Table 5. Descriptive statistics for T-cell phenotypic markers among CTN-055 CHOICES participants with and without hepatitis C virus (HCV), and PLWH Ref. group

Variable	Group	N	Mean	Std Dev	Lower 95%	Upper 95%	Median	Lower Quartile	Upper Quartile
CD3+CD4+	AUD+ HCV-W0	15	44.99	13.08	37.74	52.23	45.10	32.40	54.50
CD3+CD4+	AUD+ HCV+W0	7	43.66	12.60	32.01	55.31	42.60	34.70	49.00
CD3+CD4+	AUD+ HCV-W4	15	44.97	13.98	37.23	52.71	50.10	31.70	55.50
CD3+CD4+	AUD+ HCV+W4	7	46.21	9.10	37.79	54.63	44.30	38.90	52.50
CD3+CD4+	PLWH Ref	22	43.50	14.31	37.15	49.85	46.00	32.10	54.90
CD3+CD4+TEMRA CD45RA+CCR7- CD27+ Geo Mean	AUD+ HCV-W0	15	6267.60	1749.39	5298.82	7236.38	6709.00	4786.00	7920.00
CD3+CD4+TEMRA CD45RA+CCR7-CD27+ Geo Mean	AUD+ HCV+W0	7	6459.14	810.22	5709.81	7208.47	6753.00	5763.00	7179.00
CD3+CD4+TEMRA CD45RA+CCR7- CD27+ Geo Mean	AUD+ HCV-W4	15	5779.80	1609.23	4888.64	6670.96	5854.00	4057.00	7271.00
CD3+CD4+TEMRA CD45RA+CCR7- CD27+ Geo Mean	AUD+ HCV+W4	7	6384.43	1173.20	5299.40	7469.46	6169.00	5469.00	7180.00
CD3+CD4+TEMRA CD45RA+CCR7- CD27+ Geo Mean	PLWH Ref	22	6009.09	1636.74	5283.40	6734.78	6299.50	5095.00	6922.00
CD3+CD4+ EM CD45RA-CCR7-CD27+ Geo Mean	AUD+ HCV-W0	15	6103.13	928.69	5588.84	6617.42	5769.00	5348.00	6892.00
CD3+CD4+ EM CD45RA-CCR7-CD27+ Geo Mean	AUD+ HCV+W0	7	5969.71	1031.71	5015.55	6923.88	6041.00	5231.00	6567.00
CD3+CD4+ EM CD45RA-CCR7-CD27+ Geo Mean	AUD+ HCV-W4	15	5836.27	922.07	5325.64	6346.89	5414.00	5120.00	6658.00
CD3+CD4+ EM CD45RA-CCR7-CD27+ Geo Mean	AUD+ HCV+W4	7	6303.00	1231.84	5163.74	7442.26	6856.00	5241.00	7521.00
CD3+CD4+ EM CD45RA-CCR7-CD27+ Geo Mean	PLWH Ref	22	5820.23	866.94	5435.85	6204.61	5731.50	5231.00	6479.00
CD3+CD4+TEMRA CCR7-CD45RA+	AUD+ HCV-W0	15	1.83	1.97	0.74	2.92	1.08	0.51	3.25
CD3+CD4+TEMRA CCR7-CD45RA+	AUD+ HCV+W0	7	1.17	0.60	0.62	1.73	1.35	0.47	1.63

CD3+CD4+TEMRA CCR7-CD45RA+	AUD+ HCV-W4	15	1.97	2.47	0.60	3.33	1.00	0.43	2.07
CD3+CD4+TEMRA CCR7-CD45RA+	AUD+ HCV+W4	7	1.27	0.89	0.44	2.10	0.80	0.62	2.28
CD3+CD4+TEMRA CCR7-CD45RA+	PLWH Ref	22	1.47	1.41	0.84	2.09	0.87	0.36	2.52
CD3+CD4+ EM CCR7-CD45RA-	AUD+ HCV-W0	15	37.16	15.30	28.68	45.64	35.10	29.20	46.70
CD3+CD4+ EM CCR7-CD45RA-	AUD+ HCV+W0	7	43.43	13.28	31.15	55.71	39.90	35.90	48.00
CD3+CD4+ EM CCR7-CD45RA-	AUD+ HCV-W4	15	36.62	13.72	29.02	44.22	39.10	24.00	43.70
CD3+CD4+ EM -CD45RA-	AUD+ HCV+W4	7	39.66	13.55	27.13	52.19	36.10	33.00	55.40
CD3+CD4+ EM CCR7-CD45RA-	PLWH Ref	22	42.33	13.58	36.31	48.35	39.60	31.20	55.00
CD3+CD4+ N CCR7+CD45RA+	AUD+ HCV-W0	15	19.78	11.73	13.29	26.28	17.50	12.80	24.30
CD3+CD4+ N CCR7+CD45RA+	AUD+ HCV+W0	7	15.14	8.33	7.44	22.84	13.10	9.00	24.10
CD3+CD4+ N CCR7+CD45RA+	AUD+ HCV-W4	15	20.83	11.08	14.69	26.97	18.70	14.30	26.90
CD3+CD4+ N CCR7+CD45RA+	AUD+ HCV+W4	7	17.21	10.24	7.74	26.68	11.70	9.53	23.80
CD3+CD4+ N CCR7+CD45RA+	PLWH Ref	22	13.78	7.70	10.37	17.19	12.65	8.23	22.60
CD3+CD4+ CM CCR7+CD45RA-	AUD+ HCV-W0	15	41.23	10.70	35.31	47.16	41.60	36.70	46.60
CD3+CD4+ CM CCR7+CD45RA-	AUD+ HCV+W0	7	40.26	10.58	30.47	50.04	44.80	30.10	49.10
CD3+CD4+ CM CCR7+CD45RA-	AUD+ HCV-W4	15	40.61	10.03	35.05	46.16	41.00	36.50	43.90
CD3+CD4+ CM CCR7+CD45RA-	AUD+ HCV+W4	7	41.86	8.04	34.42	49.30	44.40	31.50	47.30
CD3+CD4+ CM CCR7+CD45RA-	PLWH Ref	22	42.41	12.13	37.04	47.79	43.40	31.80	53.60
CD3+CD4+ CD28-CD57+	AUD+ HCV-W0	15	9.71	12.91	2.56	16.86	5.11	0.66	11.30
CD3+CD4+ CD28-CD57+	AUD+ HCV+W0	7	5.41	4.69	1.07	9.74	4.81	1.30	8.91
CD3+CD4+ CD28-CD57+	AUD+ HCV-W4	15	10.53	14.52	2.48	18.57	4.38	0.98	12.20
CD3+CD4+ CD28-CD57+	AUD+ HCV+W4	7	4.62	3.83	1.07	8.16	4.28	0.81	8.63
CD3+CD4+ CD28-CD57+	PLWH Ref	22	7.92	8.30	4.24	11.61	7.00	1.97	11.60
CD3+CD4+ CD38+HLADR+	AUD+ HCV-W0	15	1.80	0.65	1.44	2.17	1.80	1.23	2.27
CD3+CD4+ CD38+HLADR+	AUD+ HCV+W0	7	2.42	1.90	0.67	4.18	1.83	1.54	2.33

CD3+CD4+ CD38+HLADR+	AUD+ HCV-W4	15	2.05	0.79	1.61	2.49	1.87	1.41	2.52
CD3+CD4+ CD38+HLADR+	AUD+ HCV+W4	7	3.62	3.45	0.43	6.81	2.23	1.62	5.05
CD3+CD4+ CD38+HLADR+	PLWH Ref	22	3.74	4.56	1.72	5.76	1.97	1.36	4.19
CD3+CD4+ LAG3+PD-1+	AUD+ HCV-W0	15	0.14	0.09	0.09	0.19	0.11	0.09	0.18
CD3+CD4+ LAG3+PD-1+	AUD+ HCV+W0	7	0.12	0.07	0.05	0.19	0.11	0.05	0.15
CD3+CD4+ LAG3+PD-1+	AUD+ HCV-W4	15	0.21	0.17	0.12	0.31	0.15	0.10	0.28
CD3+CD4+ LAG3+PD-1+	AUD+ HCV+W4	7	0.16	0.14	0.03	0.29	0.12	0.08	0.17
CD3+CD4+ LAG3+PD-1+	PLWH Ref	22	0.15	0.09	0.11	0.19	0.13	0.08	0.18
CD3+CD8+	AUD+ HCV-W0	15	50.95	12.24	44.17	57.72	50.00	42.80	62.00
CD3+CD8+	AUD+ HCV+W0	7	52.60	11.08	42.35	62.85	52.90	47.70	60.80
CD3+CD8+	AUD+ HCV-W4	15	51.17	13.37	43.76	58.57	46.30	40.50	64.50
CD3+CD8+	AUD+ HCV+W4	7	50.26	7.94	42.92	57.60	51.90	44.50	56.50
CD3+CD8+	PLWH Ref	22	52.66	13.40	46.72	58.60	51.35	41.30	65.00
CD3+CD4+ CCR5+	AUD+ HCV-W0	15	19.97	7.33	15.91	24.04	19.10	13.60	25.30
CD3+CD4+ CCR5+	AUD+ HCV+W0	7	21.43	7.18	14.79	28.07	20.30	15.90	28.30
CD3+CD4+ CCR5+	AUD+ HCV-W4	15	21.08	9.70	15.71	26.45	18.50	13.70	26.00
CD3+CD4+ CCR5+	AUD+ HCV+W4	7	20.86	11.11	10.58	31.13	16.30	13.70	28.60
CD3+CD4+ CCR5+	PLWH Ref	22	22.44	9.61	18.18	26.70	22.25	16.60	29.30
CD3+CD8+ CD28+	AUD+ HCV-W0	15	58.95	20.94	47.36	70.55	57.50	44.10	74.10
CD3+CD8+ CD28+	AUD+ HCV+W0	7	62.63	25.71	38.85	86.41	61.20	37.70	87.90
CD3+CD8+ CD28+	AUD+ HCV-W4	15	57.13	22.21	44.84	69.43	48.70	35.50	78.00
CD3+CD8+ CD28+	AUD+ HCV+W4	7	60.00	18.49	42.90	77.10	61.10	41.60	78.20
CD3+CD8+ CD28+	PLWH Ref	22	54.06	13.58	48.04	60.08	53.35	43.30	64.40
CD3+CD8+ CD38+	AUD+ HCV-W0	15	21.03	11.88	14.45	27.61	18.80	11.60	33.60
CD3+CD8+ CD38+	AUD+ HCV+W0	7	19.89	17.46	3.74	36.04	13.10	10.90	23.00
CD3+CD8+ CD38+	AUD+	15	21.48	12.33	14.66	28.31	18.20	10.50	27.60

	HCV-W4								
CD3+CD8+ CD38+	AUD+ HCV+W4	7	19.86	15.68	5.36	34.36	13.30	10.70	23.10
CD3+CD8+ CD38+	PLWH Ref	22	19.31	15.94	12.24	26.38	13.45	8.97	25.80
CD3+CD8+ HLADR+	AUD+ HCV-W0	15	6.14	3.39	4.27	8.02	5.14	2.93	9.41
CD3+CD8+ HLADR+	AUD+ HCV+W0	7	10.63	7.46	3.73	17.52	11.60	2.64	16.00
CD3+CD8+ HLADR+	AUD+ HCV-W4	15	6.12	3.32	4.28	7.96	5.74	2.99	8.62
CD3+CD8+ HLADR+	AUD+ HCV+W4	7	13.82	10.09	4.48	23.15	14.20	3.39	20.60
CD3+CD8+ HLADR+	PLWH Ref	22	8.62	7.94	5.10	12.14	5.84	3.71	7.66
CD3+CD8+ LAG3+	AUD+ HCV-W0	15	0.37	0.28	0.22	0.53	0.27	0.17	0.48
CD3+CD8+ LAG3+	AUD+ HCV+W0	7	0.29	0.12	0.18	0.40	0.24	0.21	0.36
CD3+CD8+ LAG3+	AUD+ HCV-W4	15	0.51	0.34	0.32	0.69	0.39	0.25	0.74
CD3+CD8+ LAG3+	AUD+ HCV+W4	7	0.44	0.22	0.23	0.64	0.36	0.29	0.67
CD3+CD8+ LAG3+	PLWH Ref	22	0.55	0.63	0.27	0.83	0.38	0.23	0.46
CD3+CD8+ PD1+	AUD+ HCV-W0	15	29.65	11.89	23.07	36.24	26.80	21.90	38.50
CD3+CD8+ PD1+	AUD+ HCV+W0	7	35.69	15.06	21.76	49.61	33.50	22.60	43.50
CD3+CD8+ PD1+	AUD+ HCV-W4	15	29.07	11.69	22.60	35.55	27.50	20.90	40.10
CD3+CD8+ PD1+	AUD+ HCV+W4	7	42.54	16.58	27.21	57.88	39.40	29.70	63.60
CD3+CD8+ PD1+	PLWH Ref	22	37.42	10.25	32.88	41.97	34.60	30.40	44.30
CD3+CD8+ CM CD45RA-CCR7+CD27+ Geo Mean	AUD+ HCV-W0	15	7304.87	1127.95	6680.23	7929.50	7211.00	6468.00	8468.00
CD3+CD8+ CM CD45RA-CCR7+CD27+ Geo Mean	AUD+ HCV+W0	7	7338.57	1569.81	5886.74	8790.40	7660.00	6066.00	8607.00
CD3+CD8+ CM CD45RA-CCR7+CD27+ Geo Mean	AUD+ HCV-W4	15	7106.07	1203.58	6439.55	7772.59	7239.00	6327.00	8173.00
CD3+CD8+ CM CD45RA-CCR7+CD27+ Geo Mean	AUD+ HCV+W4	7	7226.57	1721.29	5634.64	8818.50	8156.00	5110.00	8544.00
CD3+CD8+ CM CD45RA-CCR7+CD27+ Geo Mean	PLWH Ref	22	6385.23	1151.03	5874.89	6895.57	6500.00	5614.00	6954.00
CD3+CD8+ N	AUD+	15	8066.93	1405.35	7288.68	8845.19	7790.00	7375.00	9218.00

CD45RA+CCR7+ CD27+ Geo Mean	HCV-W0								
CD3+CD8+ N CD45RA+CCR7+ CD27+ Geo Mean	AUD+ HCV+W0	7	7746.00	1506.26	6352.95	9139.05	7362.00	6410.00	8800.00
CD3+CD8+ N CD45RA+CCR7+ CD27+ Geo Mean	AUD+ HCV-W4	15	8067.47	1490.40	7242.11	8892.82	7930.00	6989.00	9191.00
CD3+CD8+ N CD45RA+CCR7+ CD27+ Geo Mean	AUD+ HCV+W4	7	7913.29	1931.16	6127.26	9699.31	7714.00	6089.00	9134.00
CD3+CD8+ N CD45RA+CCR7+ CD27+ Geo Mean	PLWH Ref	22	6157.00	1505.91	5489.32	6824.68	5997.50	4985.00	7570.00
CD3+CD8+ TEMRA CD45RA+CCR7-CD27+ Geo Mean	AUD+ HCV-W0	15	4015.13	831.85	3554.47	4475.79	4323.00	3245.00	4581.00
CD3+CD8+ TEMRA CD45RA+CCR7-CD27+ Geo Mean	AUD+ HCV+W0	7	4369.71	1100.10	3352.29	5387.13	4829.00	3222.00	5152.00
CD3+CD8+ TEMRA CD45RA+CCR7-CD27+ Geo Mean	AUD+ HCV-W4	15	3937.60	878.93	3450.86	4424.34	3889.00	3057.00	4430.00
CD3+CD8+ TEMRA CD45RA+CCR7-CD27+ Geo Mean	AUD+ HCV+W4	7	4188.57	965.96	3295.20	5081.94	3939.00	3466.00	4664.00
CD3+CD8+ TEMRA CD45RA+CCR7-CD27+ Geo Mean	PLWH Ref	22	3687.91	564.16	3437.77	3938.04	3683.00	3363.00	3879.00
CD3+CD8+ EM CD45RA- CCR7- CD27+ Geo Mean	AUD+ HCV-W0	15	4380.47	674.56	4006.91	4754.03	4497.00	3762.00	5090.00
CD3+CD8+ EM CD45RA- CCR7- CD27+ Geo Mean	AUD+ HCV+W0	7	4351.86	615.38	3782.72	4920.99	4524.00	3981.00	4801.00
CD3+CD8+ EM CD45RA- CCR7- CD27+ Geo Mean	AUD+ HCV-W4	15	4210.20	680.23	3833.50	4586.90	4153.00	3666.00	4771.00
CD3+CD8+ EM CD45RA- CCR7- CD27+ Geo Mean	AUD+ HCV+W4	7	4433.86	671.97	3812.39	5055.32	4558.00	3649.00	4914.00
CD3+CD8+ EM CD45RA- CCR7- CD27+ Geo Mean	PLWH Ref	22	4251.73	601.91	3984.85	4518.60	4208.50	3771.00	4501.00
CD3+CD8+ TEMRA CCR7-CD45RA+	AUD+ HCV-W0	15	24.25	10.52	18.43	30.08	25.00	18.30	32.60
CD3+CD8+ TEMRA CCR7-CD45RA+	AUD+ HCV+W0	7	18.69	11.69	7.88	29.50	13.70	7.52	28.50
CD3+CD8+ TEMRA CCR7-CD45RA+	AUD+ HCV-W4	15	25.40	13.25	18.06	32.73	24.00	16.70	32.80

CD3+CD8+ TEMRA CCR7-CD45RA+	AUD+ HCV+W4	7	19.57	11.04	9.36	29.79	14.30	10.90	32.40
CD3+CD8+ TEMRA CCR7-CD45RA+	PLWH Ref	22	24.64	15.25	17.88	31.40	23.30	14.00	29.90
CD3+CD4+ CD28+	AUD+ HCV-W0	15	89.75	13.17	82.45	97.04	94.50	88.10	98.90
CD3+CD4+ CD28+	AUD+ HCV+W0	7	93.67	5.64	88.45	98.89	94.60	90.30	98.40
CD3+CD4+ CD28+	AUD+ HCV-W4	15	88.88	14.81	80.68	97.08	94.80	87.40	98.30
CD3+CD4+ CD28+	AUD+ HCV+W4	7	94.46	4.49	90.30	98.61	94.50	89.90	99.10
CD3+CD4+ CD28+	PLWH Ref	22	91.40	8.36	87.69	95.11	92.15	87.10	97.00
CD3+CD8+ EM CD45RA- CCR7- CD27+	AUD+ HCV-W0	15	47.81	13.06	40.57	55.04	52.80	38.10	53.50
CD3+CD8+ EM CD45RA- CCR7- CD27+	AUD+ HCV+W0	7	57.73	9.19	49.23	66.23	57.80	56.40	62.70
CD3+CD8+ EM CD45RA- CCR7- CD27+	AUD+ HCV-W4	15	45.59	13.13	38.32	52.87	45.00	38.90	57.60
CD3+CD8+ EM CD45RA- CCR7- CD27+	AUD+ HCV+W4	7	58.07	8.00	50.67	65.47	57.20	53.60	67.50
CD3+CD8+ EM CD45RA- CCR7- CD27+	PLWH Ref	22	54.63	13.45	48.67	60.60	54.20	47.40	66.60
CD3+CD8+ N CD45RA+CCR7+ CD27+	AUD+ HCV-W0	15	21.80	12.26	15.01	28.58	23.50	11.60	29.10
CD3+CD8+ N CD45RA+CCR7+ CD27+	AUD+ HCV+W0	7	17.22	14.29	4.00	30.44	17.80	4.31	25.80
CD3+CD8+ N CD45RA+CCR7+ CD27+	AUD+ HCV-W4	15	23.04	13.71	15.44	30.63	25.80	11.00	29.00
CD3+CD8+ N CD45RA+CCR7+ CD27+	AUD+ HCV+W4	7	16.31	12.61	4.64	27.97	14.50	4.24	27.80
CD3+CD8+ N CD45RA+CCR7+ CD27+	PLWH Ref	22	15.04	10.88	10.22	19.86	13.15	5.91	21.10
CD3+CD8+ CM CD45RA-CCR7+CD27+	AUD+ HCV-W0	15	6.12	3.90	3.96	8.28	4.66	3.50	8.92
CD3+CD8+ CM CD45RA-CCR7+CD27+	AUD+ HCV+W0	7	6.35	4.54	2.15	10.55	4.42	3.32	8.88
CD3+CD8+ CM CD45RA-CCR7+CD27+	AUD+ HCV-W4	15	5.96	4.03	3.72	8.19	4.75	3.55	8.35
CD3+CD8+ CM CD45RA-CCR7+CD27+	AUD+ HCV+W4	7	6.03	3.47	2.82	9.23	5.78	2.10	8.34
CD3+CD8+ CM CD45RA-CCR7+CD27+	PLWH Ref	22	5.69	3.22	4.26	7.12	4.65	3.03	8.39
CD3+CD8+ CD28-CD57+	AUD+	15	34.71	19.42	23.95	45.46	38.80	18.80	52.40

	HCV-W0								
CD3+CD8+ CD28-CD57+	AUD+ HCV+W0	7	30.86	23.14	9.46	52.26	26.00	7.34	55.50
CD3+CD8+ CD28-CD57+	AUD+ HCV-W4	15	35.81	20.64	24.38	47.24	35.50	19.10	50.70
CD3+CD8+ CD28-CD57+	AUD+ HCV+W4	7	32.19	18.45	15.12	49.26	33.00	11.30	50.70
CD3+CD8+ CD28-CD57+	PLWH Ref	22	34.30	13.14	28.47	40.12	35.55	23.80	42.70
CD3+CD8+ CD38+HLADR+	AUD+ HCV-W0	15	2.31	1.26	1.61	3.01	2.13	1.23	3.38
CD3+CD8+ CD38+HLADR+	AUD+ HCV+W0	7	4.24	4.31	0.25	8.23	2.96	1.23	4.52
CD3+CD8+ CD38+HLADR+	AUD+ HCV-W4	15	2.49	1.23	1.81	3.17	2.48	1.43	3.17
CD3+CD8+ CD38+HLADR+	AUD+ HCV+W4	7	4.08	2.53	1.74	6.42	3.71	1.91	5.71
CD3+CD8+ CD38+HLADR+	PLWH Ref	22	4.99	5.83	2.41	7.57	2.74	1.30	4.17
CD3+CD8+ LAG3+PD1+	AUD+ HCV-W0	15	0.21	0.14	0.13	0.29	0.20	0.09	0.32
CD3+CD8+ LAG3+PD1+	AUD+ HCV+W0	7	0.17	0.07	0.10	0.23	0.14	0.10	0.24
CD3+CD8+ LAG3+PD1+	AUD+ HCV-W4	15	0.27	0.16	0.18	0.36	0.24	0.15	0.36
CD3+CD8+ LAG3+PD1+	AUD+ HCV+W4	7	0.30	0.22	0.10	0.50	0.22	0.12	0.38
CD3+CD8+ LAG3+PD1+	PLWH Ref	22	0.37	0.52	0.14	0.61	0.22	0.13	0.31
CD3+CD14/19/56+ NKT-LIKE CELLS	AUD+ HCV-W0	15	4.45	7.19	0.47	8.44	2.30	1.24	3.52
CD3+CD14/19/56+ NKT-LIKE CELLS	AUD+ HCV+W0	7	4.27	2.60	1.86	6.68	4.55	1.82	6.25
CD3+CD14/19/56+ NKT-LIKE CELLS	AUD+ HCV-W4	15	3.46	3.34	1.61	5.31	2.41	1.31	4.86
CD3+CD14/19/56+ NKT-LIKE CELLS	AUD+ HCV+W4	7	4.16	2.56	1.80	6.53	5.69	1.27	5.87
CD3+CD14/19/56+ NKT-LIKE CELLS	PLWH Ref	22	4.48	3.86	2.77	6.19	3.47	2.10	5.26
CD3+CD4+ CD38+	AUD+ HCV-W0	15	47.53	14.44	39.53	55.53	44.10	35.90	55.40
CD3+CD4+ CD38+	AUD+ HCV+W0	7	41.47	13.80	28.71	54.23	40.50	33.90	53.40
CD3+CD4+ CD38+	AUD+ HCV-W4	15	50.14	15.04	41.81	58.47	51.20	33.40	58.40
CD3+CD4+ CD38+	AUD+ HCV+W4	7	45.24	12.78	33.42	57.06	43.10	35.10	58.80
CD3+CD4+ CD38+	PLWH Ref	22	44.68	14.28	38.35	51.01	43.40	34.80	53.60
CD3+CD4+ HLADR+	AUD+ HCV-W0	15	5.61	2.17	4.41	6.82	5.93	3.61	7.64

CD3+CD4+ HLADR+	AUD+ HCV+W0	7	8.56	6.61	2.45	14.66	6.47	4.08	9.68
CD3+CD4+ HLADR+	AUD+ HCV-W4	15	5.72	2.07	4.58	6.87	5.04	3.93	7.77
CD3+CD4+ HLADR+	AUD+ HCV+W4	7	9.97	8.78	1.85	18.09	6.42	5.41	11.20
CD3+CD4+ HLADR+	PLWH Ref	22	9.22	10.06	4.76	13.69	5.99	4.21	9.47
CD3+CD4+ LAG3+	AUD+ HCV-W0	15	0.25	0.19	0.15	0.36	0.18	0.12	0.33
CD3+CD4+ LAG3+	AUD+ HCV+W0	7	0.20	0.13	0.09	0.32	0.18	0.10	0.27
CD3+CD4+ LAG3+	AUD+ HCV-W4	15	0.35	0.27	0.20	0.50	0.23	0.16	0.70
CD3+CD4+ LAG3+	AUD+ HCV+W4	7	0.27	0.15	0.13	0.41	0.22	0.18	0.33
CD3+CD4+ LAG3+	PLWH Ref	22	0.25	0.17	0.18	0.33	0.22	0.15	0.27
CD3+CD4+ PD1+	AUD+ HCV-W0	15	30.45	11.54	24.06	36.84	32.30	20.10	37.20
CD3+CD4+ PD1+	AUD+ HCV+W0	7	30.50	8.14	22.97	38.03	29.20	25.80	33.00
CD3+CD4+ PD1+	AUD+ HCV-W4	15	30.21	13.67	22.64	37.77	22.80	21.00	37.10
CD3+CD4+ PD1+	AUD+ HCV+W4	7	29.01	9.59	20.15	37.88	26.40	23.30	29.30
CD3+CD4+ PD1+	PLWH Ref	22	34.39	13.25	28.52	40.26	30.95	28.10	34.80
CD3+CD4+ CM CCR7+CD45RA- Geo Mean	AUD+ HCV-W0	15	9331.20	1156.34	8690.84	9971.56	9326.00	8391.00	9900.00
CD3+CD4+ CM CCR7+CD45RA- Geo Mean	AUD+ HCV+W0	7	9263.14	1669.67	7718.95	10807.33	10216.00	7959.00	10430.00
CD3+CD4+ CM CCR7+CD45RA- Geo Mean	AUD+ HCV-W4	15	9098.93	1361.22	8345.11	9852.75	9249.00	7954.00	9978.00
CD3+CD4+ CM CCR7+CD45RA- Geo Mean	AUD+ HCV+W4	7	9408.86	1650.58	7882.33	10935.39	10268.00	7440.00	10325.00
CD3+CD4+ CM CCR7+CD45RA- Geo Mean	PLWH Ref	22	8013.14	1545.00	7328.12	8698.15	8051.00	6502.00	8907.00
CD3+CD4+ N CCR7+CD45RA Geo Mean	AUD+ HCV-W0	15	8948.73	1475.40	8131.68	9765.78	9078.00	7789.00	9882.00
CD3+CD4+ N CCR7+CD45RA+	AUD+ HCV+W0	7	8800.14	1236.56	7656.51	9943.77	8607.00	7880.00	9844.00
CD3+CD4+ N CCR7+CD45RA+	AUD+ HCV-W4	15	8776.67	1543.01	7922.18	9631.16	9211.00	7440.00	10119.00

Geo Mean									
CD3+CD4+ N CCR7+CD45RA+ Geo Mean	AUD+ HCV+W4	7	8956.29	1830.80	7263.08	10649.49	9114.00	7259.00	9437.00
CD3+CD4+ N CCR7+CD45RA+ Geo Mean	PLWH Ref	22	7622.50	1501.33	6956.85	8288.15	7609.00	6438.00	8859.00

All data reported as frequencies unless indicated as Geometric (Geo) Mean.

Abbreviations: TEMRA (effector memory T-cells re-expresses CD45RA); N (naïve); CM (central memory); EM (effector memory); NKT-like (natural-killer T-cell-like)

Supplemental Table 6. Spearman correlation analysis between sCD40L and TGF-β

	**CTN-055**				**PLWH**	
	AUD+C+		AUD+C-		AUD-C-	
Comparison	Correlation (r_s)	p-value	Correlation (r_s)	p-value	Correlation (r_s)	p-value
TGF- β1 (pg/mL) vs TGF-β2 (pg/mL)	0.4198	0.1351	0.6174	**0.0005**	0.7737	**0.0001**
TGF-β1 (pg/mL) vs sCD40L (ng/mL)	0.6352	**0.0147**	0.6951	<**0.0001**	0.5737	**0.0102**
TGF-β2 (pg/mL) vs sCD40L (ng/mL)	0.2000	0.4930	0.4904	**0.0081**	0.4965	**0.0306**

Supplemental Table 7. Plasma cytokine values among CTN-055 CHOICES participants with and without hepatitis C virus (HCV), and PLWH Ref. group

Variable	Group	N	Mean	Std Dev	Lower 95%	Upper 95%	Median	Lower Quartile	Upper Quartile
Fractalkine	AUD+ HCV-W0	15	4085.96	2820.45	2524.04	5647.88	3432.37	2478.90	4432.43
Fractalkine	AUD+ HCV+W0	7	3971.46	1778.84	2326.31	5616.61	4479.56	2200.80	5652.72
Fractalkine	AUD+ HCV-W4	15	3951.66	2638.19	2490.68	5412.64	3377.35	2538.46	4351.94
Fractalkine	AUD+ HCV+W4	7	4240.13	752.54	3544.14	4936.11	4116.47	3948.76	4746.88
Fractalkine	PLWH Ref	22	3331.67	2585.41	2185.36	4477.98	2899.34	2400.77	3235.06
GMCSF	AUD+ HCV-W0	15	0.12	0.40	-0.10	0.35	0.02	0.00	0.04
GMCSF	AUD+ HCV+W0	7	0.00	0.01	0.00	0.01	0.00	0.00	0.00
GMCSF	AUD+ HCV-W4	15	0.24	0.91	-0.26	0.75	0.00	0.00	0.03
GMCSF	AUD+ HCV+W4	7	0.00	0.01	0.00	0.01	0.00	0.00	0.00
GMCSF	PLWH Ref	22	0.01	0.02	0.00	0.02	0.00	0.00	0.03
IFNα	AUD+ HCV-W0	15	12.03	21.19	0.30	23.76	1.97	0.00	10.86
IFNα	AUD+ HCV+W0	7	1.60	1.18	0.51	2.69	1.98	0.00	2.13
IFNα	AUD+ HCV-W4	15	3.97	10.17	-1.67	9.60	1.93	0.00	2.08
IFNα	AUD+ HCV+W4	7	1.84	1.34	0.60	3.08	2.26	0.00	2.82
IFNα	PLWH Ref	22	5.78	13.25	-0.09	11.66	2.06	0.00	2.85
IFNβ	AUD+ HCV-W0	15	14.44	44.08	-9.97	38.85	0.00	0.00	0.41
IFNβ	AUD+ HCV+W0	7	4.63	10.01	-4.63	13.88	0.00	0.00	5.54
IFNβ	AUD+ HCV-W4	15	23.52	91.10	-26.93	73.97	0.00	0.00	0.00
IFNβ	AUD+ HCV+W4	7	14.42	20.99	-4.99	33.83	0.00	0.00	29.80
IFNβ	PLWH Ref	22	139.14	428.59	-50.88	329.17	0.00	0.00	14.04
IFNγ	AUD+ HCV-W0	15	60.30	146.74	-20.96	141.56	9.25	6.63	15.38
IFNγ	AUD+ HCV+W0	7	5.56	6.07	-0.05	11.18	5.17	0.00	9.58

IFNγ	AUD+ HCV-W4	15	47.56	159.53	-40.79	135.90	4.75	0.00	15.94
IFNγ	AUD+ HCV+W4	7	87.20	148.15	-49.81	224.21	34.75	11.02	77.62
IFNγ	PLWH Ref	22	9.48	15.41	2.64	16.31	5.04	0.00	9.17
IL10	AUD+ HCV-W0	15	1.00	2.10	-0.17	2.16	0.10	0.00	0.88
IL10	AUD+ HCV+W0	7	0.39	0.53	-0.10	0.88	0.29	0.00	0.41
IL10	AUD+ HCV-W4	15	0.17	0.20	0.06	0.28	0.10	0.00	0.29
IL10	AUD+ HCV+W4	7	1.94	4.82	-2.52	6.40	0.15	0.00	0.36
IL10	PLWH Ref	22	0.91	2.10	-0.03	1.84	0.14	0.00	0.48
IL12p70	AUD+ HCV-W0	15	2.47	6.27	-1.00	5.95	0.05	0.00	0.30
IL12p70	AUD+ HCV+W0	7	0.05	0.06	0.00	0.11	0.04	0.00	0.10
IL12p70	AUD+ HCV-W4	15	1.66	5.62	-1.45	4.77	0.16	0.07	0.41
IL12p70	AUD+ HCV+W4	7	0.18	0.15	0.04	0.31	0.13	0.08	0.32
IL12p70	PLWH Ref	22	0.13	0.24	0.02	0.23	0.06	0.00	0.10
IL15	AUD+ HCV-W0	15	16.97	22.98	4.24	29.69	4.45	1.45	24.94
IL15	AUD+ HCV+W0	7	7.72	6.89	1.35	14.10	2.99	2.25	13.29
IL15	AUD+ HCV-W4	15	6.35	11.48	-0.01	12.71	2.29	1.43	3.50
IL15	AUD+ HCV+W4	7	3.49	3.27	0.47	6.52	2.50	2.02	4.29
IL15	PLWH Ref	22	6.12	14.10	-0.14	12.37	1.77	1.45	4.86
IL17A	AUD+ HCV-W0	15	113.83	321.62	-64.27	291.94	1.02	0.00	2.47
IL17A	AUD+ HCV+W0	7	82.45	110.34	-19.61	184.50	32.71	0.42	231.53
IL17A	AUD+ HCV-W4	15	34.58	129.46	-37.11	106.27	0.70	0.00	2.71
IL17A	AUD+ HCV+W4	7	69.01	170.72	-88.88	226.90	2.10	0.00	21.61
IL17A	PLWH Ref	22	55.03	236.18	-49.68	159.75	0.45	0.00	3.66
IL18	AUD+ HCV-W0	15	456.32	156.38	369.71	542.92	494.01	366.67	565.71
IL18	AUD+	7	484.18	115.07	377.76	590.61	436.30	420.92	628.57

	HCV+W0								
IL18	AUD+ HCV-W4	15	433.20	144.28	353.31	513.10	428.10	316.64	582.24
IL18	AUD+ HCV+W4	7	483.91	58.79	429.54	538.28	500.39	406.29	536.57
IL18	PLWH Ref	22	414.34	147.70	348.85	479.83	385.55	301.51	509.88
IL1β	AUD+ HCV-W0	15	2.90	5.46	-0.12	5.92	0.49	0.11	2.41
IL1β	AUD+ HCV+W0	7	2.54	3.98	-1.14	6.22	1.26	0.29	2.67
IL1β	AUD+ HCV-W4	15	1.02	1.39	0.25	1.78	0.30	0.04	1.83
IL1β	AUD+ HCV+W4	7	2.05	4.65	-2.25	6.35	0.23	0.16	0.92
IL1β	PLWH Ref	22	1.74	5.77	-0.82	4.30	0.09	0.00	0.66
IL2	AUD+ HCV-W0	15	0.10	0.40	-0.12	0.32	0.00	0.00	0.00
IL2	AUD+ HCV+W0	7	0.01	0.02	-0.01	0.03	0.00	0.00	0.00
IL2	AUD+ HCV-W4	15	0.03	0.12	-0.04	0.10	0.00	0.00	0.00
IL2	AUD+ HCV+W4	7	4.00	10.58	-5.79	13.78	0.00	0.00	0.00
IL2	PLWH Ref	22	0.00	0.00	.	.	0.00	0.00	0.00
IL21	AUD+ HCV-W0	15	575.73	965.71	40.93	1110.52	29.92	0.00	1429.09
IL21	AUD+ HCV+W0	7	6.17	16.32	-8.93	21.26	0.00	0.00	0.00
IL21	AUD+ HCV-W4	15	217.07	545.57	-85.06	519.19	7.45	0.00	46.73
IL21	AUD+ HCV+W4	7	20.33	24.18	-2.03	42.69	14.97	0.00	26.54
IL21	PLWH Ref	22	198.43	694.36	-109.43	506.30	5.34	0.00	25.43
IL22	AUD+ HCV-W0	15	13.20	22.04	0.99	25.40	1.73	0.55	31.14
IL22	AUD+ HCV+W0	7	3.23	5.51	-1.86	8.33	0.92	0.49	4.08
IL22	AUD+ HCV-W4	15	3.28	7.59	-0.92	7.49	1.05	0.71	1.40
IL22	AUD+ HCV+W4	7	1.80	1.96	-0.01	3.62	0.84	0.55	2.85
IL22	PLWH Ref	22	4.96	12.03	-0.37	10.29	0.75	0.47	2.14
IL23	AUD+ HCV-W0	15	0.74	1.31	0.01	1.46	0.00	0.00	0.75

IL23	AUD+ HCV+W0	7	0.34	0.34	0.02	0.65	0.34	0.00	0.71
IL23	AUD+ HCV-W4	15	2.23	5.79	-0.97	5.44	0.50	0.00	1.84
IL23	AUD+ HCV+W4	7	1.32	1.16	0.25	2.39	0.71	0.50	2.13
IL23	PLWH Ref	22	0.98	2.06	0.07	1.90	0.25	0.00	0.93
IL27	AUD+ HCV-W0	15	517.39	722.35	117.37	917.41	125.36	93.08	1228.45
IL27	AUD+ HCV+W0	7	198.54	113.75	93.34	303.74	180.89	144.48	245.76
IL27	AUD+ HCV-W4	15	272.56	485.35	3.78	541.34	116.92	76.54	218.01
IL27	AUD+ HCV+W4	7	106.38	35.33	73.71	139.05	111.05	87.81	142.20
IL27	PLWH Ref	22	296.66	670.90	-0.80	594.12	131.53	80.93	210.58
IL29	AUD+ HCV-W0	15	3.50	3.57	1.53	5.48	2.70	1.37	4.44
IL29	AUD+ HCV+W0	7	11.73	4.89	7.20	16.25	11.84	7.97	16.25
IL29	AUD+ HCV-W4	15	3.83	4.59	1.29	6.37	2.72	0.98	4.50
IL29	AUD+ HCV+W4	7	10.10	5.44	5.07	15.14	8.69	6.21	14.23
IL29	PLWH Ref	22	7.13	10.31	2.55	11.70	3.00	0.74	8.56
IL33	AUD+ HCV-W0	15	1.40	3.09	-0.32	3.11	0.57	0.00	1.06
IL33	AUD+ HCV+W0	7	0.47	0.37	0.13	0.82	0.53	0.00	0.65
IL33	AUD+ HCV-W4	15	1.13	2.85	-0.45	2.71	0.55	0.00	0.60
IL33	AUD+ HCV+W4	7	0.58	0.50	0.12	1.04	0.56	0.00	1.11
IL33	PLWH Ref	22	3.72	10.35	-0.87	8.30	0.59	0.00	1.04
IL4	AUD+ HCV-W0	15	7.10	19.38	-3.63	17.84	0.17	0.00	1.90
IL4	AUD+ HCV+W0	7	3.23	5.71	-2.05	8.51	0.36	0.07	4.31
IL4	AUD+ HCV-W4	15	2.67	9.53	-2.61	7.94	0.00	0.00	0.64
IL4	AUD+ HCV+W4	7	4.53	10.04	-4.76	13.82	0.13	0.00	3.01
IL4	PLWH Ref	22	2.38	8.80	-1.52	6.29	0.00	0.00	0.21
IL6	AUD+	15	13.05	31.08	-4.17	30.26	1.06	0.65	4.18

	HCV-W0								
IL6	AUD+ HCV+W0	7	7.49	10.28	-2.01	16.99	2.18	0.44	16.48
IL6	AUD+ HCV-W4	15	11.79	18.76	1.40	22.18	0.92	0.56	24.46
IL6	AUD+ HCV+W4	7	7.94	15.94	-6.80	22.68	1.92	0.39	3.83
IL6	PLWH Ref	22	7.37	20.89	-1.89	16.63	0.60	0.30	2.08
IL7	AUD+ HCV-W0	15	49.38	66.80	12.38	86.37	14.62	6.19	75.06
IL7	AUD+ HCV+W0	7	21.18	21.73	1.08	41.27	10.77	8.92	30.15
IL7	AUD+ HCV-W4	15	17.07	23.59	4.01	30.13	10.35	5.59	12.95
IL7	AUD+ HCV+W4	7	7.84	2.87	5.18	10.50	7.87	4.60	10.35
IL7	PLWH Ref	22	14.93	21.41	5.43	24.42	7.02	4.83	14.72
IL8	AUD+ HCV-W0	15	312.75	721.49	-86.79	712.30	33.05	12.92	208.43
IL8	AUD+ HCV+W0	7	541.68	810.36	-207.78	1291.13	322.25	30.28	683.23
IL8	AUD+ HCV-W4	15	195.39	489.69	-75.79	466.57	28.34	22.48	66.71
IL8	AUD+ HCV+W4	7	180.35	399.70	-189.32	550.01	37.08	9.15	62.06
IL8	PLWH Ref	22	89.53	255.78	-23.87	202.94	10.14	7.88	35.39
IL9	AUD+ HCV-W0	15	7.29	15.43	-1.25	15.83	0.41	0.07	4.18
IL9	AUD+ HCV+W0	7	5.22	8.32	-2.48	12.92	0.83	0.00	13.69
IL9	AUD+ HCV-W4	15	7.10	12.21	0.34	13.86	0.23	0.00	16.02
IL9	AUD+ HCV+W4	7	3.55	8.95	-4.73	11.82	0.08	0.00	0.70
IL9	PLWH Ref	22	1.28	4.72	-0.81	3.37	0.07	0.00	0.28
IP10	AUD+ HCV-W0	15	1143.45	473.07	881.48	1405.43	1199.51	666.88	1617.02
IP10	AUD+ HCV+W0	7	1988.30	1564.81	541.10	3435.51	1893.42	587.10	2318.04
IP10	AUD+ HCV-W4	15	1071.18	502.20	793.07	1349.28	1081.47	596.62	1440.49
IP10	AUD+ HCV+W4	7	1932.29	1049.66	961.52	2903.06	1854.53	969.75	2778.44
IP10	PLWH Ref	22	1679.10	3867.07	-35.46	3393.66	871.70	586.52	1101.93

ITAC	AUD+ HCV-W0	15	81.13	45.52	55.92	106.33	72.52	55.69	89.09
ITAC	AUD+ HCV+W0	7	52.85	13.77	40.12	65.59	56.90	46.36	63.21
ITAC	AUD+ HCV-W4	15	81.11	61.31	47.15	115.06	71.80	48.76	88.48
ITAC	AUD+ HCV+W4	7	50.75	20.47	31.82	69.68	47.83	37.67	71.53
ITAC	PLWH Ref	22	61.00	55.70	36.31	85.70	42.61	35.05	60.42
MCP3	AUD+ HCV-W0	15	12.74	32.15	-5.07	30.54	1.94	1.30	7.30
MCP3	AUD+ HCV+W0	7	18.86	18.87	1.41	36.31	20.36	0.82	40.46
MCP3	AUD+ HCV-W4	15	7.36	15.32	-1.13	15.84	1.38	0.00	2.16
MCP3	AUD+ HCV+W4	7	11.16	20.47	-7.78	30.09	2.10	1.88	7.16
MCP3	PLWH Ref	22	1.38	1.50	0.71	2.05	0.96	0.83	1.50
MIP1α	AUD+ HCV-W0	15	22.91	23.81	9.72	36.10	15.20	10.54	23.39
MIP1α	AUD+ HCV+W0	7	18.99	8.94	10.72	27.26	18.46	9.46	25.54
MIP1α	AUD+ HCV-W4	15	26.62	34.34	7.60	45.64	12.04	7.05	35.09
MIP1α	AUD+ HCV+W4	7	12.93	2.93	10.22	15.65	13.09	10.95	14.67
MIP1α	PLWH Ref	22	22.14	42.61	3.24	41.03	10.84	7.43	16.08
MIP3α	AUD+ HCV-W0	15	41.08	70.39	2.10	80.06	15.68	10.43	22.93
MIP3α	AUD+ HCV+W0	7	11.41	9.15	2.94	19.87	8.74	4.63	21.14
MIP3α	AUD+ HCV-W4	15	35.36	74.10	-5.67	76.40	18.20	9.29	24.89
MIP3α	AUD+ HCV+W4	7	40.23	29.22	13.20	67.26	26.91	16.73	71.02
MIP3α	PLWH Ref	22	13.26	11.04	8.36	18.16	10.80	7.36	14.82
SDF1α	AUD+ HCV-W0	15	461.27	373.11	254.65	667.89	296.99	210.11	639.18
SDF1α	AUD+ HCV+W0	7	438.23	200.02	253.24	623.22	440.49	237.99	577.93
SDF1α	AUD+ HCV-W4	15	426.36	202.59	314.17	538.55	397.27	271.41	469.07
SDF1α	AUD+ HCV+W4	7	377.08	161.71	227.52	526.63	325.38	271.17	450.34
SDF1α	PLWH	22	301.81	157.25	232.09	371.53	283.63	222.33	353.00

	Ref								
TGFβ1	AUD+ HCV-W0	15	19235.13	9741.91	13840.24	24630.02	20476.56	14057.74	27775.71
TGFβ1	AUD+ HCV+W0	7	19334.37	12176.49	8072.99	30595.74	21943.67	10251.56	28753.96
TGFβ1	AUD+ HCV-W4	15	27142.73	11959.54	20519.76	33765.71	21012.91	17845.05	39541.95
TGFβ1	AUD+ HCV+W4	7	19380.35	11727.75	8533.99	30226.71	18277.67	14004.16	30207.63
TGFβ1	PLWH Ref	22	16176.29	8142.55	12566.09	19786.50	17117.57	13850.27	20187.32
TGFβ2	AUD+ HCV-W0	15	106.97	71.41	67.43	146.52	84.90	51.29	161.82
TGFβ2	AUD+ HCV+W0	7	75.56	49.60	29.69	121.43	85.41	32.31	121.36
TGFβ2	AUD+ HCV-W4	15	125.90	95.48	73.03	178.77	90.93	66.25	158.57
TGFβ2	AUD+ HCV+W4	7	63.37	45.77	21.04	105.70	62.82	34.98	118.53
TGFβ2	PLWH Ref	22	45.50	32.88	30.93	60.08	36.03	30.85	52.65
TGFβ3	AUD+ HCV-W0	15	0.53	0.53	0.24	0.82	0.44	0.10	0.75
TGFβ3	AUD+ HCV+W0	7	0.23	0.18	0.07	0.39	0.24	0.07	0.31
TGFβ3	AUD+ HCV-W4	15	0.97	2.70	-0.52	2.47	0.23	0.00	0.66
TGFβ3	AUD+ HCV+W4	7	0.10	0.12	-0.02	0.21	0.00	0.00	0.23
TGFβ3	PLWH Ref	22	0.21	0.24	0.11	0.32	0.19	0.00	0.42
TNFα	AUD+ HCV-W0	15	28.19	63.87	-7.18	63.56	1.45	0.53	13.53
TNFα	AUD+ HCV+W0	7	25.42	40.96	-12.47	63.30	3.14	0.31	64.71
TNFα	AUD+ HCV-W4	15	21.55	38.59	0.18	42.92	0.73	0.11	34.01
TNFα	AUD+ HCV+W4	7	11.73	30.05	-16.06	39.52	0.45	0.00	0.81
TNFα	PLWH Ref	22	11.83	40.33	-6.05	29.71	0.55	0.25	5.49
